# Peripheral Myelin Protein-22 and Its Prominence in Charcot-Marie-Tooth Disease

**DOI:** 10.1021/acs.chemrev.6c00096

**Published:** 2026-05-20

**Authors:** Charles R. Sanders, Bruce D. Carter, Mason C. Wilkinson, Geoffrey C. Li, Katherine M. Stefanski

**Affiliations:** † Department of Biochemistry, 12327 Vanderbilt University School of Medicine − Basic Sciences, Nashville, Tennessee 37240, United States; ‡ Vanderbilt Center for Structural Biology, Vanderbilt University, Nashville, Tennessee 37240, United States; ⊥ Vanderbilt Institute for Chemical Biology, Vanderbilt University, Nashville, Tennessee 37240, United States; § Vanderbilt Brain Institute, Nashville, Tennessee 37240, United States

## Abstract

Charcot-Marie-Tooth disease (CMT) is an often-debilitating peripheral neuropathy that is a top-10 most prevalent human genetic disorder. However, there is currently no effective treatment. Over half of diagnosed CMT cases in western populations are caused by genetic variations that alter the expression levels or sequence of peripheral myelin protein 22 (PMP22). PMP22 is a tetraspan membrane glycoprotein that is most highly expressed in Schwann cells (SCs) of the peripheral nervous system (PNS) under conditions of myelination, where it plays multiple important roles. These functions are reduced in humans with only a single *PMP22* allele, resulting in a common and usually mild form of CMT, hereditary neuropathy with liability to pressure palsies (HNPP). The rare type 1E CMT (CMT1E) is caused by amino acid variations in PMP22. The most common form of CMT (CMT1A) is caused by a third wild type (WT) allele of PMP22. The disease mechanisms of CMT1A are still incompletely understood, but there is much evidence that a major driver is proteostasis stress caused by WT PMP22 overexpression upon induction of myelination in SCs, possibly compounded by gain-of-function effects. Here, we explore PMP22’s structure, functions, trafficking, role in CMT, and prospects for successful therapeutic intervention.

## Graphical abstract

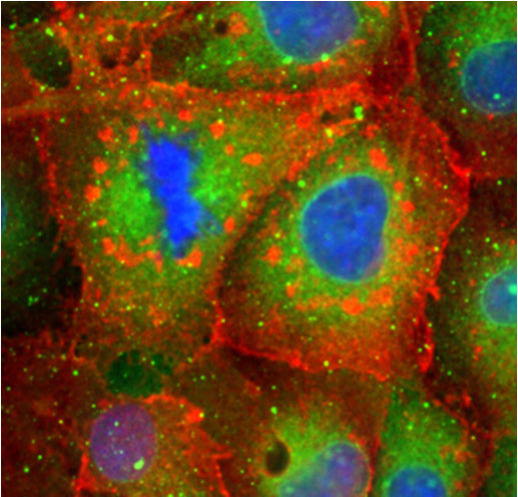

## 1 Introduction to the PMP22 Protein and to Charcot-Marie-Tooth Disease (CMT)

Peripheral myelin protein 22 (PMP22, once referred to as Gas3, SR13, or PASII) is a 160-residue tetraspan membrane glycoprotein that plays important but enigmatic roles in the myelination of axons in the peripheral nervous system (PNS). Overexpression, underexpression, or amino acid variations in the human PMP22 protein can result in Charcot-Marie-Tooth disease (CMT).^1–15^ The authors of this review are approaching the PMP22 protein and its related biology and pathobiology from the standpoints of biochemistry, chemical biology, and biophysics, with the goals of reviewing what is currently known regarding the molecular basis for both the healthy functions of this protein and how it plays central roles in CMT. Over half of diagnosed CMT cases are caused by gene variations that affect the expression levels or sequence of the PMP22 protein.^16–18^ More specifically, type 1A CMT (CMT1A) is caused by the presence of a third allele encoding PMP22, while loss of a PMP22 allele results in hereditary neuropathy with liability to pressure palsies (HNPP), which is regarded as a form of CMT in this Review. More rare are type 1E CMT (CMT1E) variants that alter, frameshift, or truncate the open reading frame of *PMP22* or that alter regulation of *PMP22* transcription, translation, or RNA splicing.^18–22^

CMT is an inherited disease and, as such, is classified as a rare disorder.^1–4,6,8,12,14,15,23–25^ However, among the thousands of known rare inherited disorders, CMT is in the top 10 in terms of disease prevalence,^26^ afflicting on the order of 1:5000 to 1:2500 humans.^27–32^ CMT is a peripheral neuropathy that typically becomes evident in childhood or adolescence and then progresses with age.^1,2,4,6,8,12–15^ It is only rarely fatal, and many patients live normal lifespans.^32^ Disease symptoms and severity vary based on which gene variation is causative.^1–4,6,8,12,14,15,17,23–25,32^ Moreover, patient-specific disease-modifying factors can result in dramatic variability in age of onset and/or disease severity, even within patient groups who share the same CMT gene variant.^33–36^

There are no approved drugs for treating CMT. It can be conservatively estimated that an effective drug to treat the most common form of the disease (CMT1A) would have a worldwide market of roughly $1 billion/year (based on assuming 1:2500 people have CMT, that 50% of them have CMT1A, that 25% of patients have access to the drug, that the drug would need to be taken over the full lifespan, and that the cost of the drug is $2500/year per patient).

CMT is considered a “length dependent” neuropathy, meaning that symptoms usually start at the furthest parts of the body from the central nervous system (CNS) and peripheral nervous system (PNS) junctions along the spinal cord, almost always the feet.^1–4,6,8,12–15,23–25,32^ As the disease progresses, the lower legs, hands, and forearms are impacted. For the PMP22-linked forms of CMT typical symptoms are very high foot arches, distorted toes (“hammer toes”), very thin calves, distorted hand and fingers, distal muscle atrophy, and weakness. In certain forms of CMT patients experience pain, numbness, tremors, problems speaking, problems with balance, and/or deafness. As of early-2026 there is no cure or therapy for CMT beyond orthotics and physical therapy,^32^ although gene therapy is being pursued and there are also other therapeutic modalities in clinical trials.^37^ Beyond the population of diagnosed CMT patients, it is suspected that there are many people who suffer from mild forms of CMT but have never been diagnosed (c.f., refs 38–40 ).

While the focus of this review is on the PMP22 protein and its roles in health and disease, we first provide two background introductory sections important for understanding PMP22 and the forms of CMT it causes. Section 2 gives a brief overview of the process of myelination of axons in the peripheral nervous system, in which PMP22 plays its most important physiological roles. Section 3 provides an overview of the genetics of CMT, where the relationship of the PMP22-linked forms of CMT to other forms of this disease is briefly summarized.

## 2 Myelination in the Peripheral Nervous System

Axons serve as conduits for the action potential, connecting neuron cell bodies to neuromuscular junctions (synapses) or sensory endings.^41,42^ The physiological defects that cause CMT are generally localized to myelinated axons of the peripheral nervous system. Not all axons are myelinated. Small caliber (<1 μM) axons of all kinds are usually not myelinated, which includes many of the axons responsible for transmitting sensation of heat, pain, and certain forms of touch, plus postganglionic autonomic axons.^41^ Motor neuron axons and preganglionic autonomic fibers with high calibers (>1–2 μm in diameter) are myelinated, which enables the rapid “saltatory conduction” of the action potential. The pathology of PMP22-linked forms of CMT is most closely related to demyelination and the resulting degeneration and loss of motor neuron axons (Figure 1).^43^ The outermost protective layer of the master cable is the epineurium. Within it is a bundle of individual cables, each surrounded by its own protective layer (the perineurium) and containing many individual axons.

**Figure 1:**
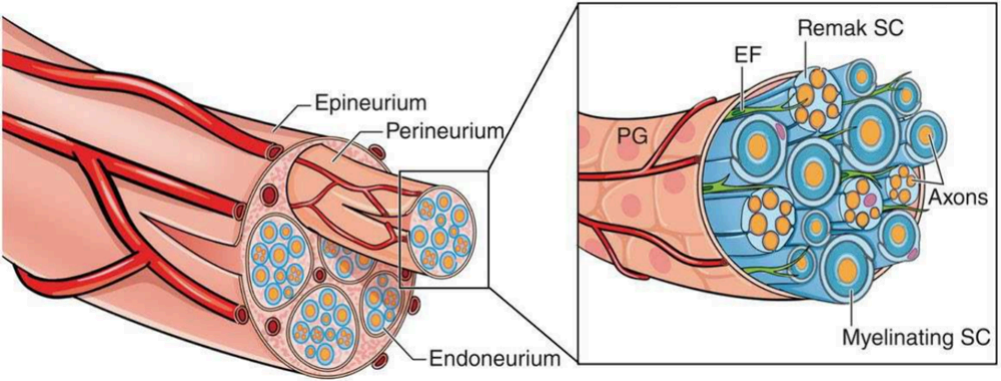
Superstructure of a peripheral nerve cable. The perineurium surrounds the endoneurium, which (right) contains large axons that are myelinated by Schwann cells (SCs) (dark blue), smaller axons that are engaged by SCs to form Remak bundles, and endoneural fibroblasts (EFs) (green). These fascicles are joined together by the epineurium. The vasculature is also shown (red). This caption is adapted and figure is reproduced with permission from Zotter et al. 2022 (Figure 1A) and used with permission.^44^ Copyright 2022 Society for Neuroscience.

The smaller, nonmyelinated axons are not naked, but are engulfed by “Remak” Schwann cells, each of which encases multiple small caliber axons.^45^ The larger axons are myelinated. Unlike the central nervous system (CNS), where oligodendrocyte cells generate myelin, it is Schwann cells that generate myelin in the PNS.^46^ Also, while a single CNS oligodendrocyte generates many segments of myelin and does so for multiple axons, each myelinating Schwann cell pairs with only a single axon to generate a single myelinated segment. Schwann cells are therefore lined up side-by-side along each PNS axon to provide the electrical insulation—myelin—required for rapid conduction of the action potential through the axon (Figure 2A). Between each myelin segment along the axon is a short unmyelinated segment, the node of Ranvier, which we henceforth will refer to as “the node” (Figure 2B). The axonal membrane at the node is the location of the voltage-activated sodium channels that sense the initial transmembrane voltage jump of the action potential, resulting in channel opening that amplifies electrical transmembrane depolarization as the action potential passes along the axon to the next node. Potassium channels restore the axonal membrane to a resting transmembrane voltage to complete the action potential and are located in the juxtaparanodal regions of myelinated axonal membranes. The potassium channels in the juxtaparanodes are kept separated from the nodal sodium channels by the paranodes, where the leaflet loops of myelin contact the axon and form paranodal junctions that act as membrane diffusion barriers (Figure 2C).

**Figure 2:**
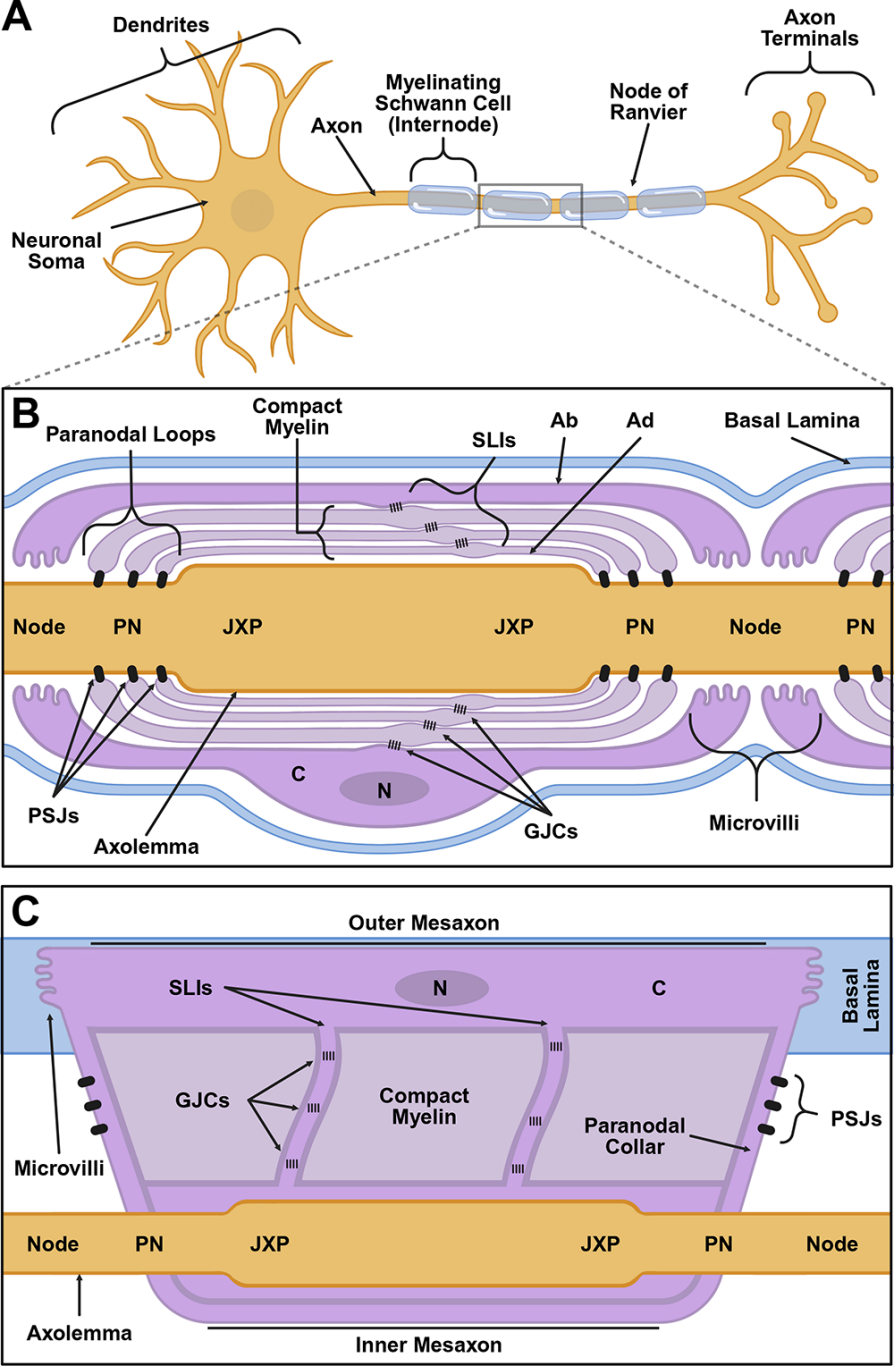
Schematic of a myelinated peripheral nerve. (A) Schwann cells spirally wrap their plasma membranes around the axon to form the insulating myelin layer. Myelinating Schwann cells comprise the internodal region of the nerve—interspersed between them are exposed axonal segments called the nodes of Ranvier, which contain sodium ion channels that facilitate the saltatory conduction of the action potential. (B) Longitudinal cross section of a myelinating Schwann cell. The Schwann cell nucleus (N) is situated outside of the compacted myelin sheath. The cytosolic interiors of each double membrane of the myelin membrane tongues are continuous with the cytosol (C) of the SC body via spiraling and also via a series of connected inclusions known as Schmidt–Lanterman incisures (SLIs). Gap junction complexes (GJCs) form channels through SLI membranes to facilitate direct cytoplasmic access from the main body of the Schwann cell to the adaxonal membrane, which directly contacts the axon membrane (axolemma). Myelin begins to decompact at distal regions known as the juxtaparanode (JXP), from which cytoplasmic loops further extend to contact the axon at the paranode (PN) adjacent to the node of Ranvier. Paranodal septate junction complexes (PSJs) form a tight seal at the interface of the paranodal loops and the axolemma, insulating the node of Ranvier from the central internode. Microvilli extend further past the paranode to encapsulate the node of Ranvier itself. Schwann cells additionally attach their outermost membrane, the abaxonal membrane, to the basal lamina supporting an extracellular matrix that encases both the node and the internode. (C) Unwrapped view of a myelinating Schwann cell. The extended SLIs more clearly demonstrate the direct linkage of the Schwann cell cytoplasm and the periaxonal space. The paranodal collar at the boundaries of the Schwann cell similarly provides a cytoplasmic path to the paranodal loops. Lastly, the mesaxons indicate edge-to-edge contacts between the Schwann cell plasma membrane regions: the inner and outer mesaxons are formed at the interface between the myelin sheath with innermost (adaxonal) or outermost (abaxonal) Schwann cell plasma membrane layers, respectively. Created in BioRender. (2026) https://BioRender.com/37bsctn.

Myelin is generated following “radial sorting” in which a bundle of axons and Schwann cell precursors codevelop either into Remak SCs with engulfed small-caliber axons or into promyelinating SCs, wherein each new SC locates itself next to an large-caliber axon and extends a tongue-like lamellipodia that initially encircles the axon by a single (loose) turn, generating the abaxonal membrane (Figure 3).^41,42,45,47–49^ The promyelinating SCs then generate a protective basal lamina that encases both the cell and its associated juxtaposed axonal segment.^50^

**Figure 3:**
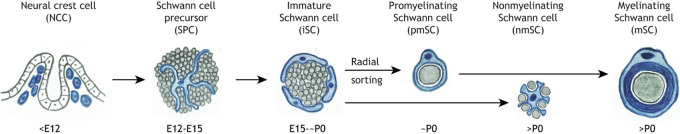
Timeline of Schwann cell maturation and differentiation in mice. Approximate time windows for each stage are shown below their corresponding images. “E” stands for days before birth. “P” stands for days after birth. Following radial sorting, immature Schwann cells can differentiate into either myelinating or nonmyelinating Schwann cells. Caption adapted and figure reproduced with permission from Fledrich et al. 2019 (Figure 2).^48^ Copyright 2019 The Company of Biologists Ltd.

In response to signals from the now encased axonal segment,^41,51^ the promyelinating SC then becomes a myelinating SC, entering a period of “radial growth”. During radial growth the tip of the lamellipodia tongue of the roughly tubular promyelinating SC maintains axonal contact as the tongue extends and repetitively spirals around the axon. During radial growth the SC body, anchored to the extracellular matrix, undergoes no radial migration but supplies the membrane that enables the tongue to continue to extend its radial extension until formation of the axon segment-encompassing myelin unit is complete (Figure 4).

**Figure 4:**
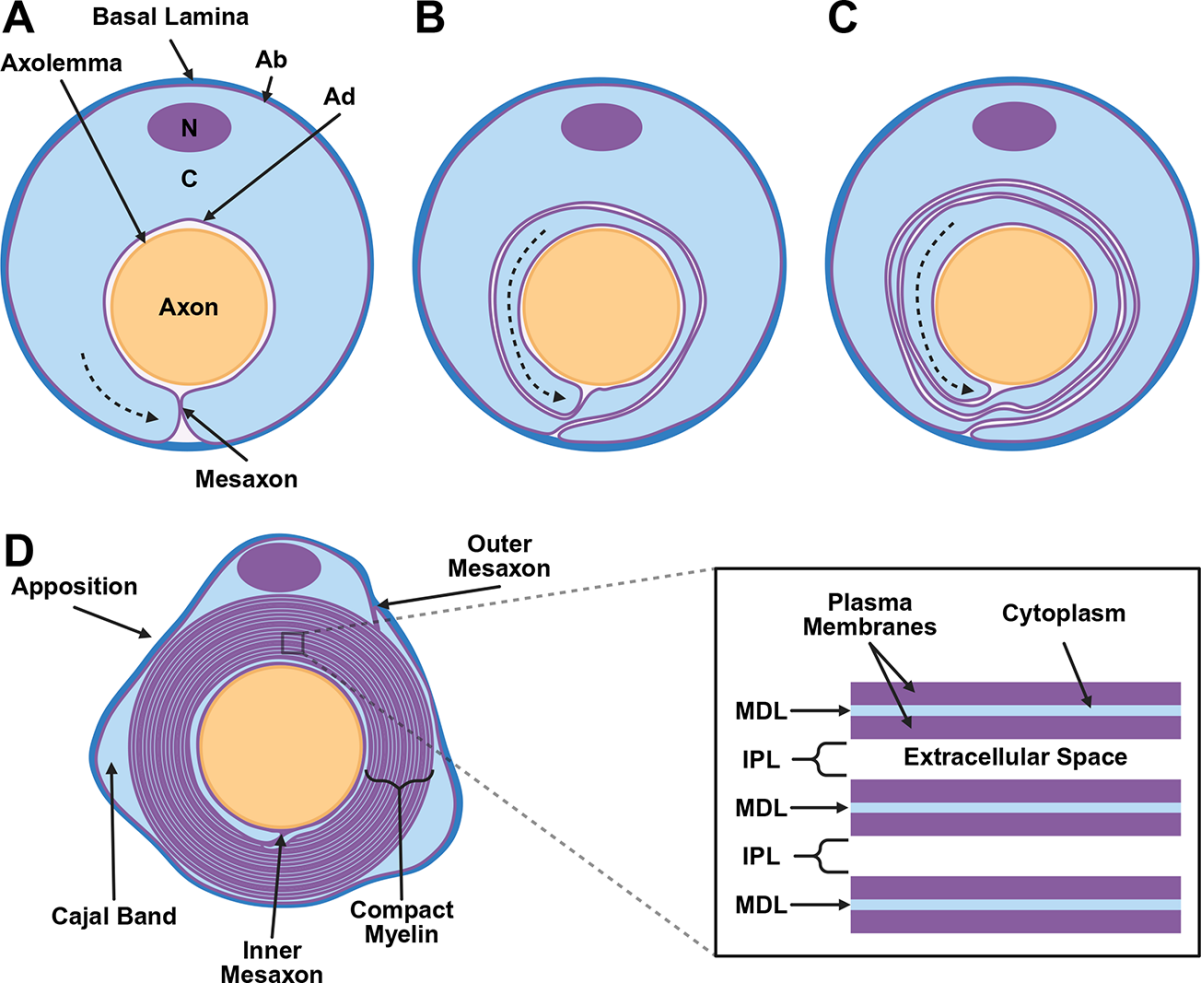
Formation of compact myelin by Schwann cells. Panel (A) represents the starting point for myelination, which is after radial sorting where a SC wraps around an axonal segment and forms a basal lamina. The mesaxon is formed when the Schwann cell plasma membranes from either side of the axon contact. (B) Myelination begins. One of the mesaxonal membranes displaces the other, extending beneath it to grow adaxonally (flush with the axolemma). The dashed arrow indicates the direction of growth. The immature basal lamina retained from radial sorting also begins to mature during this time. (C) The subducted membrane extends around the axon many more times (radial expansion), continually displacing, compactifying, and adding new layers to the growing myelin sheath. (D) The myelinating Schwann cell has completed the myelin sheath and its basal lamina has fully matured. Cytoplasmic inclusions and exclusions known as Cajal bands and appositions, respectively, can be visualized. The inset of compact myelin shows the alternating pattern of the major dense line (MDL) and the intraperiod line. The MDL is a 3 nm layer formed from the apposition of cytoplasmic membrane leaflets, while the IPL is a 4–5 nm layer correspondingly formed from the apposition of extracellular membrane leaflets. Created in BioRender. (2026) https://BioRender.com/ks3tvjn.

The inside of the membrane-bounded tongue is contiguous with the parent SC cytosol, appearing as the “major dense line” in electron micrographs of myelinated axon section (Figure 5). The space outside the tongue is contiguous with the extracellular space and appears as the “intraperiod line” in micrographs. Myelinating SCs are considered to be a type of polarized cells, with multiple parallels to the organization and biology of epithelial cells.^42,52,53^ Adhesive points of contact are formed between the axonal membrane and both the innermost spiral from the Schwann cell (the “adaxonal membrane”) and the tips of the paranodal loops located between compact myelin and the node (Figure 2B).^54^

**Figure 5:**
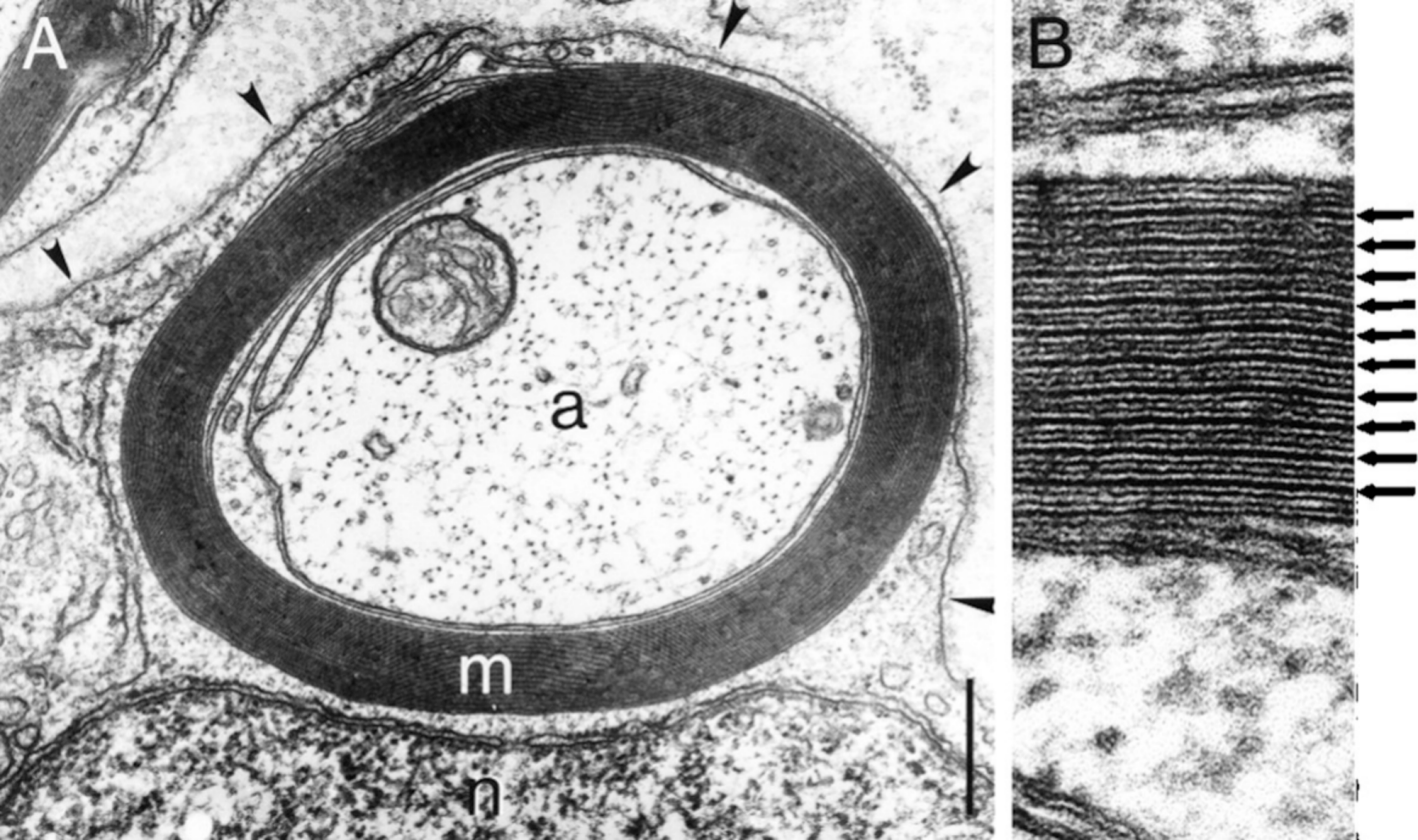
(A) Electron micrograph of a myelinated fiber from a sciatic nerve of a five-day old mouse showing the axon (a), myelin (m), Schwann cell nucleus (n), and basal lamina (arrowheads). Scale bar, 0.5 mm; (B) Electron micrograph showing compact myelin made up of alternating major dense lines (arrows) and intraperiod lines. Caption adapted and figure reprinted with permission from Scherer (1997) (Figure 1).^55^ Copyright 1997 Elsevier.

The central domain of the sheath is composed mainly of tightly wound layers “compact myelin”, while the paranodal domains are composed of noncompacted membrane loops: the paranodal loops. The Schwann cells also extend microvilli that project over the node but do not adhere to the axon. Between compact myelin and the juxtaparanodal domain is the paranodal region, under which—on the axonal membrane—is the location of the voltage-based potassium channels involved in saltatory conduction.

A healthy large caliber PNS myelinated segment usually contains 40 or more layers of membrane, alternatively separated by extracellular space and cytosol from membrane layer to layer^41^ (Figure 5) of wound myelin. The total caliber of the axon plus its sheath is typically in the range of 2–20 μm, where the g ratio (axon diameter/total diameter) is on the order of 0.6.^41^ The longitudinal “internodal” length of a myelinated segment in human and mice is typically on the order of 1–2 mm, bordered on each side by 1–2 μm nodes of Ranvier. A single myelin sheath can contain 20 mm^2^ of membrane.^41^ Generation of a mature myelin sheath by a Schwann cell therefore involves a prodigious expansion of the total membrane bilayer surface area relative to the premyelinating Schwann cell, estimated to increase by a factor of several thousand.^56^ The energy requirements for synthesizing the lipids and proteins required for such a huge membrane expansion are massive. Indeed, SCs seem to have specially adapted mitochondria to support the production of unusually large amounts of ATP.^57^

The role of myelinating Schwann cells in supporting axons is more than merely conferring mechanical stability to myelin segments and serving as a biological form of an electrical tape. For example, myelinating Schwann cells serve to deliver energy to axons.^58,59^ Closely associated with this function, myelin includes physical channels called Schmidt–Lanterman incisures (SLIs) that span the abaxonal to adaxonal membrane^60^ in series (Figures 2B, 2C, and 6). Closely associated with the SLIs are junctions (tight, adherens, and gap junctions) that play adhesive, boundary, and passive transport functions.^61,62^ Gap junctions provide molecular tunnels between the layers of myelin^62,63^ that allow nutrients and other small molecules to pass directly from the abaxonal myelin layers to the innermost layers and even to the axon, bypassing the need to spirally diffuse around each layer of myelin to reach the axon. These nutrients are important not just for SC and myelin homeostasis but are also used to locally fuel axonal segments, which are often far away from the nerve cell body.^48,51,64^ Some tight junctions also form paracellular channels that allow certain solutes (usually ions) to pass reversibly between the apical extracellular space on one side of a tight junction and the basolateral extracellular space on the other side.^65,66^ The tight and adherens junctions are usually proximal, with the adherens junctions being associated with a well-organized bundled actomyosin belt and the tight junctions being associated with a less well-ordered belt composed of a branched actin/myosin mesh (Figure 6).

**Figure 6:**
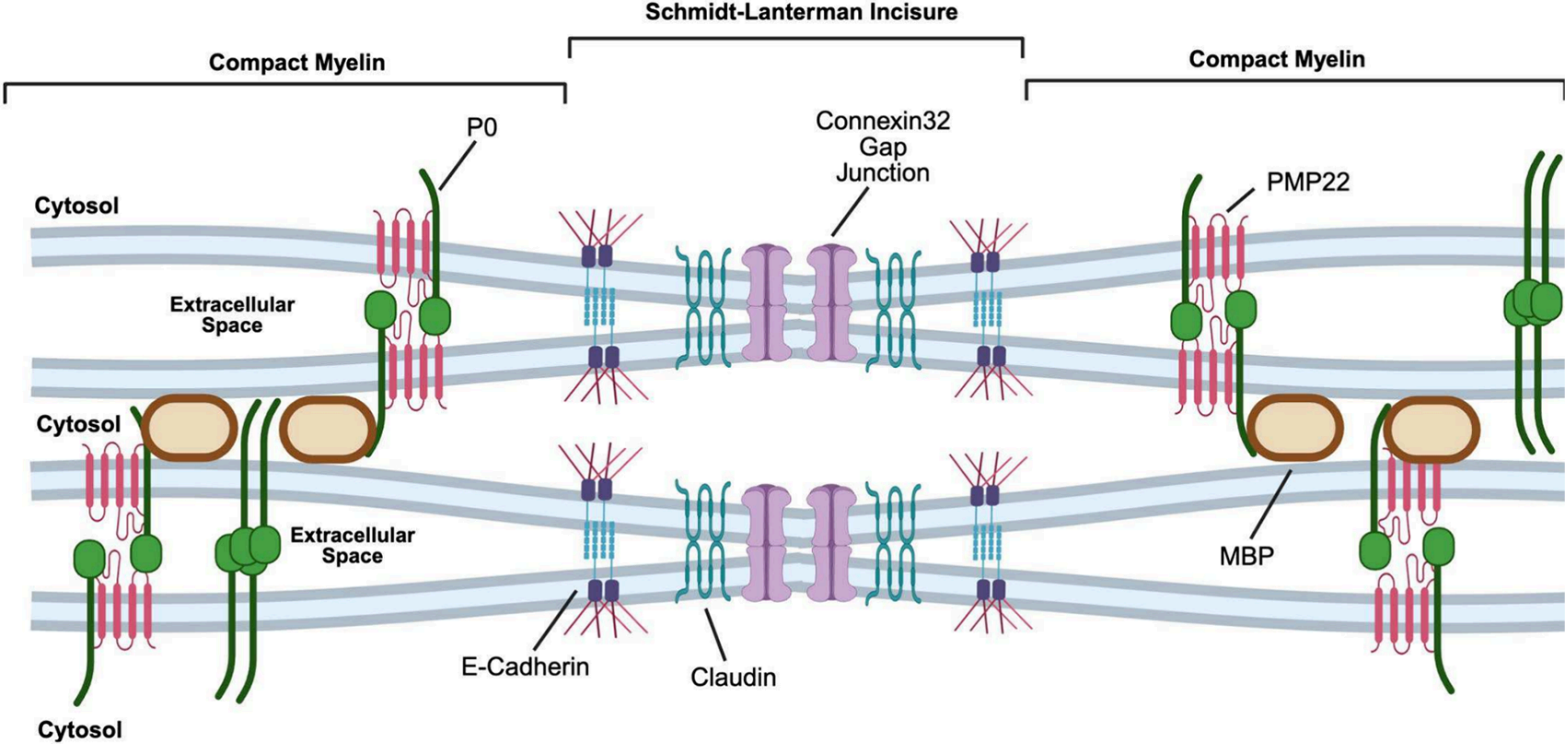
Schematic of Schmidt–Lanterman incisure (SLI) with adherens (E-cadherin-based), tight (claudin-based) and gap (connexin-based) junctions, flanked on both sides by compact myelin. PMP22 may play a role in these junctions, but exactly how is unclear. The interactions depicted between P_o_, MBP, and PMP22 that results in adhesion and proper spacing in compact myelin is emphasized here, but the modes and stoichiometries of interaction depicted are hypothetical and there are other proteins involved. The stoichiometry of homomerization and heteromerization should not be taken to be as depicted. See ref 67. Created in BioRender. Stefanski, K. (2026) https://BioRender.com/xeezj5g.

There is extensive molecular signaling crosstalk between SCs and axons,^48,51,68,69^ which is critical for initiating and controlling myelination, as well as also to help maintain and alter healthy axonal properties (regulating axon caliber, for example). Indeed, while the two major classes of CMT are demyelinating CMT and axon-based CMT, Schwann cells and myelinated axons to some degree function as a unit. Therefore, anything that significantly perturbs a SC or its associated myelin sheath may impact the organization and function of axons, and vice versa. Particularly prominent in promoting SC survival, proliferation, and myelination is signaling by axon-generated neuregulin-1 (NRG-1) and other growth factors to receptors located on the Schwann cell surface. These receptors include the ErbB2/3 receptor tyrosine kinases—members of the epidermal growth factor receptor family,^48,70–73^ and the p75 neurotrophic receptor—a tumor necrosis receptor family member. Within SCs, the Egr2/Krox-20, Oct6/Pou3f1, and SOX10 transcription factors are among those prominent in activating the various pathways required for myelination.^74–76^

The total Schwann cell membrane area for a mature myelinated segment is dominated by compact myelin, whose composition largely defines the total protein and lipid compositions of myelinated segments. The most abundant protein (as high as 50% of all myelin protein) is the single span membrane glycoprotein—myelin protein zero (P_0_), which forms oligomers (at least dimers of tetramers^19,77–80^ ) and is believed to provide *trans* adhesion across the extracellular space (the intraperiod line in electron micrographs) between juxtaposed bilayer surfaces (Figure 6).^79,81,82^ The second most common protein is the myelin basic protein (MBP), a weakly adhesive peripheral membrane protein that works with P_0_ to enforce membrane-to-membrane spacing spanning the cytoplasm (the major dense line) between the cytosolic leaflets of juxtaposed bilayers.^67,83–85^ The focus of this review, peripheral myelin protein 22 (PMP22), is generally cited in the literature as being much less abundant than MBP or P_0_, typically as 2–5% of total SC protein. While likely present elsewhere in myelinated segments, PMP22 is well documented to be found in compact myelin,^86–89^ where it may contribute to adhesion. Indeed, an important recent publication^78^ confirms early studies^90,91^ that PMP22 and P_0_ undergo *cis* binding in the same bilayer (Figure 7) and likely work together in *trans* to generate the adhesion between juxtaposed bilayers across extracellular space in compact myelin (Figure 6). This suggests that the levels of PMP22 might normally be tuned to be roughly stoichiometric with respect to P_0_ (although probably not 1:1). If so, this would imply that the levels of PMP22 could be higher than the oft-quoted 2–5% of total SC protein, as originally reported roughly 30 years ago. This is not inconceivable. A focused literature search by the corresponding author suggests that the widely quoted “2–5% of total protein” derives from interpretation by Snipes et al.^89^ of the densitometric profile of a single Coomassie stained gel from SDS-PAGE tube electrophoresis of PNS myelin extract, as published by Uremura and Kitamura in 1991.^92^ Without jumping to a conclusion, it is probably fair to say that that quantitation of PMP22 in myelin or myelinating SCs may be worth experimentally revisiting. PMP22 is hard to extract from myelin membranes,^93,94^ has the same N-glycoside as P_0_ and some other myelin proteins,^95^ yields no electrophoretic band if boiled in SDS-containing buffer prior to SDS-PAGE, is relatively difficult to detect immunologically, and does not stain with silver stain.

**Figure 7:**
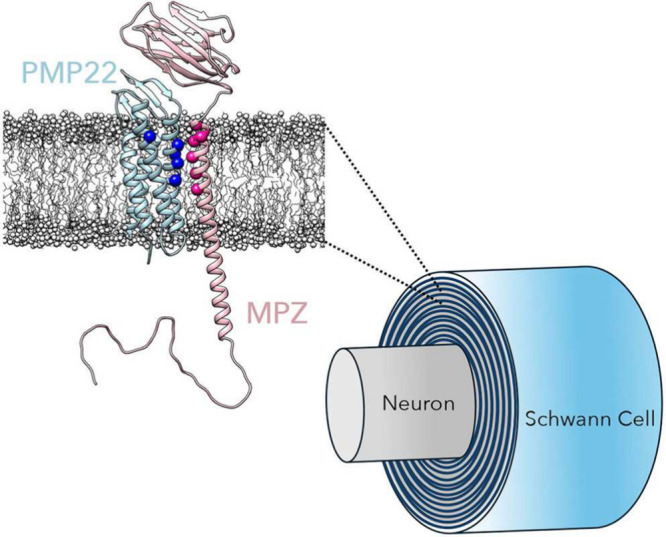
Model of the P_0_-PMP22 complex developed by Piper and colleagues based on experimental data. This figure is reproduced from Pashkova et al. 2024.^78^ Available under a CC BY 4.0 license. Copyright 2024 Natalya Pashkova, Tabitha A Peterson, Christopher P Ptak, Stanley C Winistorfer, Debbie Guerrero-Given, Naomi Kamasawa, Christopher A Ahern, Michael E Shy, Robert C Piper.

Myelin membranes have a higher level of lipid (70% by weight) than most other membranes, as befits their role in providing electrical insulation to axons. PNS myelin membranes are rich in cholesterol (37–40 mol % of lipid) and sphingolipids (24–27 mol %).^96^ Also common are sphingolipids with very long (typically C24) acyl chains, often saturated, and, for some sphingolipids, hydroxylated.^96^ Cholesterol is essential to PNS myelination.^97,98^ The burden on myelinating Schwann cells of having to make such large amounts of cholesterol and other lipids to supply their >1000-fold membrane expansion is high. SCs must make most of their own cholesterol because cholesterol in blood does not cross the blood-nerve barrier.^98^ Synthesis by SCs of each cholesterol molecule consumes 25 reducing equivalents and 18 molecules of ATP.^98^

Given how cholesterol- and sphingolipid-rich myelin membranes are, it is reasonable to suspect that certain membrane domains of myelin, such as compact myelin, may be uniformly raft-like. However, there is at least one report that the translational diffusion coefficient of P_0_ in myelin is fairly high,^99^ consistent with this protein having, at the very least, ready access to disordered phase membranes, inconsistent with the membranes of compact myelin being uniformly in the raft-like ordered phase.

Finally, the timeline for myelination in humans begs a short summary. Myelination mainly starts soon after birth and continues until PNS nerve axons are fully myelinated, typically in adolescence.^100,101^ For a given PNS axon, the number associated SC/myelin units is essentially fixed at all stages of life. If undamaged, a given SC-myelin unit is meant to persist for a human lifetime.^102–104^ As a child continues growth into adolescence, additional SC/myelin units are not generated. Rather, as PNS axons grow the already-established SC/myelin units increase their internodal lengths, requiring the production of additional myelin membrane in existing myelin segments.^105–107^ This myelin growth process continues to take place until physical adulthood, at which point the scope of the PNS is essentially fixed. At this stage the myelinating SCs are quiescent. However, PNS myelin and axons can be damaged by laceration, contusion, inflammation, toxic agents, and other factors. Damaged SCs have the virtue of being able to dedifferentiate into repair SCs, enabling damaged myelin units to be repaired or replaced, followed by reconversion into myelinating SCs (Figure 8).^47,48,104,108–110^ However, the repaired SC/myelin/axon units are not as healthy as the original tissue, even under non-CMT conditions.^104,107^ Moreover, it is thought that the regenerative capacity of SCs—as well as myelin health in general—declines with aging.^111–115^ This helps to explain the slowly progressive nature of CMT.

**Figure 8:**
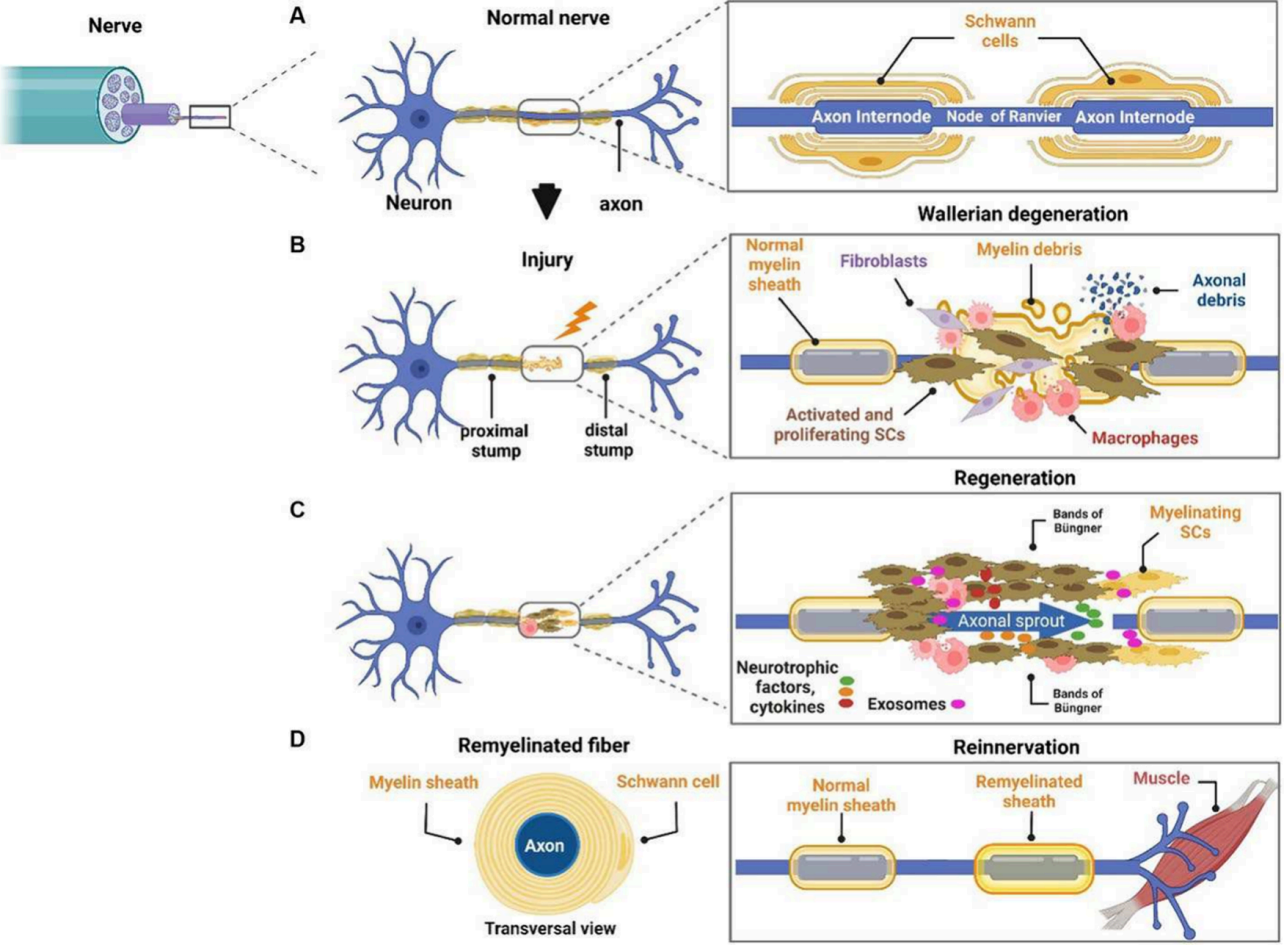
Repair of damaged myelin on peripheral nerves. (A) The normal myelin is composed of Schwann cells wrapped around the axon of a neuron. (B) When the myelin is exposed to an injury, Schwann cells dedifferentiate into repair Schwann cells with the aid of macrophages. (C) The newly differentiated Schwann cells form bands of Bungner that will promote the remyelination of axon. (D) After the repair, the axon is remyelinated by the SCs. Not illustrated in this figure is the fact that myelin decompaction and access by macrophages is facilitated by transdifferentiaion of some SCs into inflammatory demyelinating SCs.^116^ Reproduced from Figure 1 of Oliveira et al. 2023.^51^ Available under a CC BY 4.0 license. Copyright 2023 Julia Teixeira Oliveira, Christopher Yanick, Nicolas Wein, Cintia Elisabeth Gomez Limia.

## 3 Genetics of Charcot-Marie-Tooth Disease

CMT is a family of heterogeneous hereditary and sensory neuropathies. Exact classification of different genotypes and phenotypes has evolved with time. For the purposes of this Review, we use a convention where type 1 CMT (CMT1) refers to autosomal dominant demyelinating forms of the disease. This includes the most common form of CMT: CMT1A, which is caused by the presence of a third WT allele of *PMP22*. Also common is hereditary neuropathy HNPP, which is caused by loss of a *PMP22* allele. HNPP is usually a relatively mild disorder. Much less common is CMT1E, which is caused by dominant variations in *PMP22* that encode missense variations, truncations, or frameshifts in *PMP22* or that alter regulation of its transcription, translation, or splicing.

NCVs in healthy patients are 50–60 m/sec. Patients with demyelinating forms of CMT exhibit slowed motor nerve conduction velocities (NCVs) of <25–38 m/sec. Particularly severe and early onset cases of demyelinating CMT are sometimes referred to as Dejerine-Sottas syndrome (DSS), which exhibit very low NCVs of <15 m/sec. DSS can be regarded as a severe form of CMT1E. Conversely, patients with mild CMT1E are sometimes classified as having HNPP. Demyelinating CMT that is autosomal recessive is termed type-4 CMT. Although type 1 CMT and most forms of type 4 primarily affect myelin in their early disease stages, there is also associated axon degeneration, highlighting the intimate interdependence of the Schwann cell and the neuron. While the etiology of CMT1 is driven by dysmyelination and demyelination, disease symptoms are driven by the resulting degeneration and loss of demyelinated axons.^117^

Axonal forms of CMT are caused by gene variations that adversely alter axon structure, health, and function, which exert secondary impacts on myelination. Both dominant and recessive forms are termed type 2 CMT, where patient NCVs are on the order of >35–45 m/sec, approaching normal. Type X CMT is caused by dominant and recessive X-linked variations, of which by far the most common form (CMT1X) is demyelinating and caused by gene variations in *GJB1* encoding the connexin-32 gap junction channel protein. For all forms of CMT, phenotypes that exhibit NCVs in the range 25–45 m/sec are sometimes clinically classified as being “intermediate” forms of the disease: CMTi.^18^

Other peripheral neuropathies are sometimes grouped with CMT or viewed as an umbrella that includes CMT. These include hereditary motor neuron disease (HMN), hereditary sensory neuron disease, (HSN), sensory ataxic neuropathy (SAN), and Roussy-Lévy syndrome (RLS). Excellent summaries of the clinical phenotypes of the various forms of CMT, which often vary subtly from subtype to subtype, can be found in Burns et al. (2026),^32^ Pisciotta and Shy (2018)^12^ and Fridman and Reilly (2015).^118^

There has been phenomenal progress in medical genetics during the past 15 years, such that the Online Mendelian Inheritance in Man (OMIM) database^119^ now lists roughly 60 genes that are linked to CMT1/2/4/X (https://www.omim.org/). The Human Gene Mutation Database (HGMD Professional version 2024.4) lists 175 genes that have been proposed to be associated with various forms of CMT.^120^ Reilly and co-workers recently analyzed the genotypes for 1500 peripheral neuropathy patients in the UK and documented representation in this population of about 45 CMT1/2/4/X genes.^18^ This same study estimated that the overall successful diagnosis and genotyping rate for CMT patients, including those suffering from HMN, HSN, and SAN, is 77%, and the authors also found that the genotypic diagnostic success rate for patients with demyelinating CMT is an impressive 97%. In contrast, the usually rare axonal (type 2) forms of CMT remain less well genotyped, with a successful diagnosis of <50%.

Demyelinating CMT represents roughly 75% of all cases of genetically diagnosed CMT (excluding HMN, HSN, SAN, and RLS). Table 1 lists the 6 most common CMT-causing genes, as based on three large clinical genetic studies in mainly European and North American populations;^16–18^ see also ref.^34^ It can be seen that CMT1A caused by PMP22 gene duplication (resulting in a third wild-type allele) is the most common cause of CMT, causing over 50% of all diagnosed cases. However, this trend does not seem to hold in Japan, where 23% of CMT cases are CMT1A,^121^ and may not hold in other parts of Asia and wider world, where genetic data for CMT patients is, in many cases, not yet well developed.^32^

**Table 1:** Genetic Causes of the 7 Most Common Diagnosed Forms of CMT from Three Large-Population Patient Studies^a^

Gene/form of CMT	DiVincenzo et al.^17^	Record et al.^18^	Fridman et al.^16^
*PMP22* (3rd allele)/CMT1A	**57%**	**55%**	**61.6%**
*PMP22* (loss of allele) HNPP	**22%**	**6.1%**	**3.2%**
*GJB1* variants/CMT1X	**6.7%**	**15%**	**11%**
*MPZ* variants/CMT1B	**5.3%**	**4.2%**	**6.9%**
*MFN2* variants/CMT2A	**4.3%**	**4.8%**	**7.2%**
*PMP22* variants/CMT1E	**0.9%**	**1.0%**	**1.4%**
*SH3TC2* variants/CMT4B1	**0.8%**	**2.8%**	**1.4%**

a The percentages are based on total numbers of diagnosed cases of CMT in three different large (>1000) patient populations and exclude patients diagnosed with HMN, NSN, and SAN.

The *PMP22 gene* duplication event is the consequence of chromosomal fragment recombination that results (in the germline cells of a founder patient) in either duplication or elimination of that *PMP22*-containing fragment (Figure 9). Roughly 10%–20% of all cases of CMT1A are believed to occur due to this causative recombination event occurring in a germline cell of a founder,^12,122^ in which case this state of trisomy is then dominantly heritable from generation to generation.

**Figure 9:**
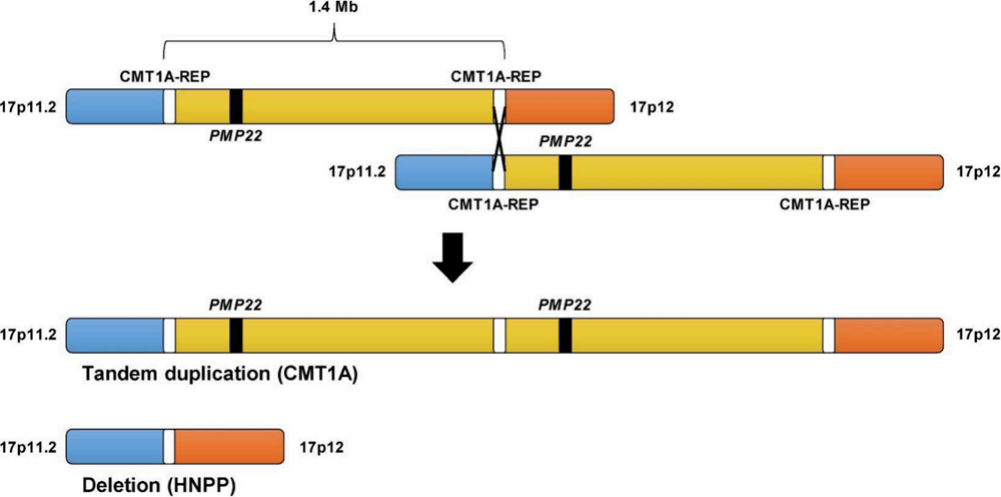
Chromosomal fragment recombination resulting in duplication or deletion of the 1.4 Mb segment containing the *PMP22* gene. When this occurs in a germline cell (egg or sperm), fertilized eggs that have the chromosome with duplicated *PMP22* will result in CMT1A, while the fertilized eggs with the deletion will result in hereditary neuropathy with liability to pressure palsy (HNPP). Reproduced with permission from Figure 1 of Pantera al. 2020.^123^ Copyright 2020 Elsevier.

The germline recombination event in a founder patient that results in trisomy of *PMP22* also results in gene deletion, leaving the affected patient with only a single remaining copy of *PMP22*. This leads to HNPP, a usually mild genetically dominant disease that is grouped with CMT.^34,124,125^

Both CMT1A and HNPP arise from nonallelic homologous recombination during meiosis, mediated by misalignment of flanking low-copy repeats. Thus, crossover in one direction leads to duplication while in the other direction deletion. Therefore, de novo mutation rates should theoretically be symmetric, suggesting a similar prevalence, although there could be modifiers, such as reproductive fitness. As illustrated in Table 1, the milder HNPP seems to be much less prevalent than CMT1A, as was also reported in a recent analysis of over 4000 CMT1A, CMT1E, and HNPP cases.^34^ A likely explanation is that HNPP patients often experience such mild symptoms that the disorder is diagnosed only later in life or not at all.^38,40^ Indeed, the variability in reported prevalence seen for HNPP among the three studies summarized in Table 1 likely reflects, in part, different efficiencies of diagnosis of HNPP within different patient populations.

For both HNPP and CMT1A, the age of clinical onset, disease severity, and progression can vary significantly among patients.^33–35,117^

One objective of this Review is to explore what is known about the role of PMP22 in both health and in CMT1A, CMT1E, and HNPP. But first, an introduction to the structure, possible functions, and interactions of the PMP22 protein is necessary.

## 4 The PMP22 Protein Is a Member of an Evolutionarily Related Family of Membrane Proteins

It has been known for 25 years that PMP22 is highly homologous to the epithelial membrane proteins (EMP1, EMP2, and EMP3).^126,127^ Homology is also clear (Figure 10) to MP20/LIM2—the lens fiber intrinsic membrane protein,^128^ to PERP—the “p53 apoptosis effector related to PMP22,^129,130^ to the claudin family of tight junction proteins, and to the TARPs: the transmembrane AMPA receptor regulatory proteins, which include stargazin (CACNG2) and are also known as “calcium channel γ-subunits” because some modulate voltage-gated calcium channels. All these proteins share the same tetraspan membrane topology as PMP22, with four transmembrane helices, a single intracellular loop, cytosolically disposed N- and C- termini, and two extracellular loops.

**Figure 10:**
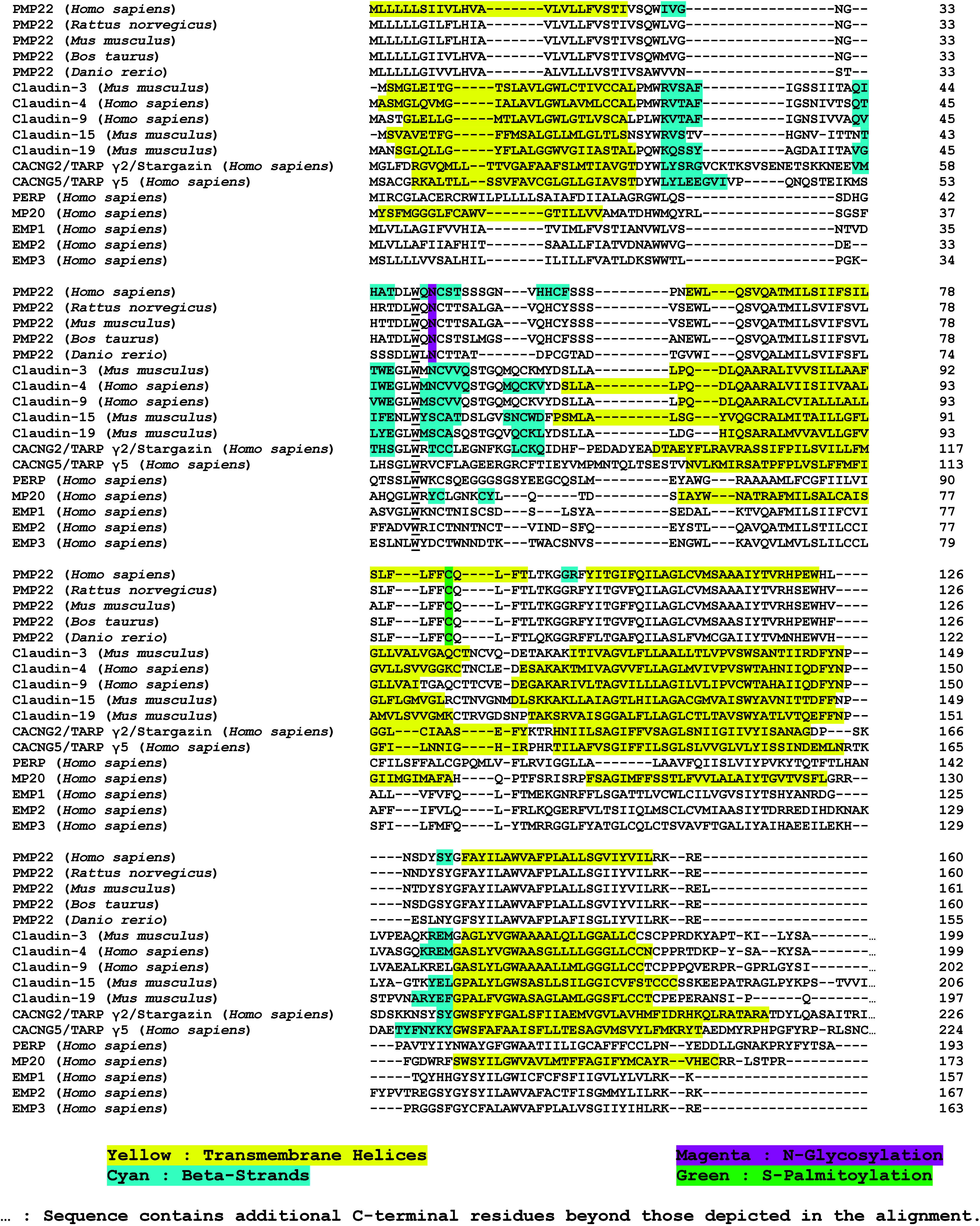
Sequence alignment (manual) for PMP22 and selected homologues, all of which share the claudin fold. Residues and segments are colored coded only if they are supported by experimental data.

There is currently no experimental 3D structure of PMP22. The first experimental structure for a PMP22 homologue was the X-ray crystal structure of claudin-15 determined by the Fujiyoshi lab in 2014 (Figure 11).^131^ This was followed both by additional experimental claudin structures,^132^ as well as by experimental TARP structures, sometimes in complex with AMPAR.^133–139^ These structures share two defining structural features that appear to be conserved in PMP22 and other homologues. Namely, the transmembrane domain is composed of a left-handed 4-helix bundle that sets up the organization of an extracellular 5-strand antiparallel β-pleated sheet domain. Four strands are contributed by extracellular loop 1 (ECL1), the first strand of which emerges following a short segment connecting it to the end of transmembrane helix 1 (TM1). The fifth strand is contributed by the much shorter ECL2 and flanks the first strand from ECL1. We refer here to this two-domain structure as the “claudin fold”. All claudin and TARP structures also share a conserved disulfide bond connecting beta-strands 3 and 4 near the end of ECL1. Based on the alignments shown in Figure 10 it appears that all the PMP22 homologues listed there include this disulfide bond, although the exact location and interresidue spacing can vary.

**Figure 11:**
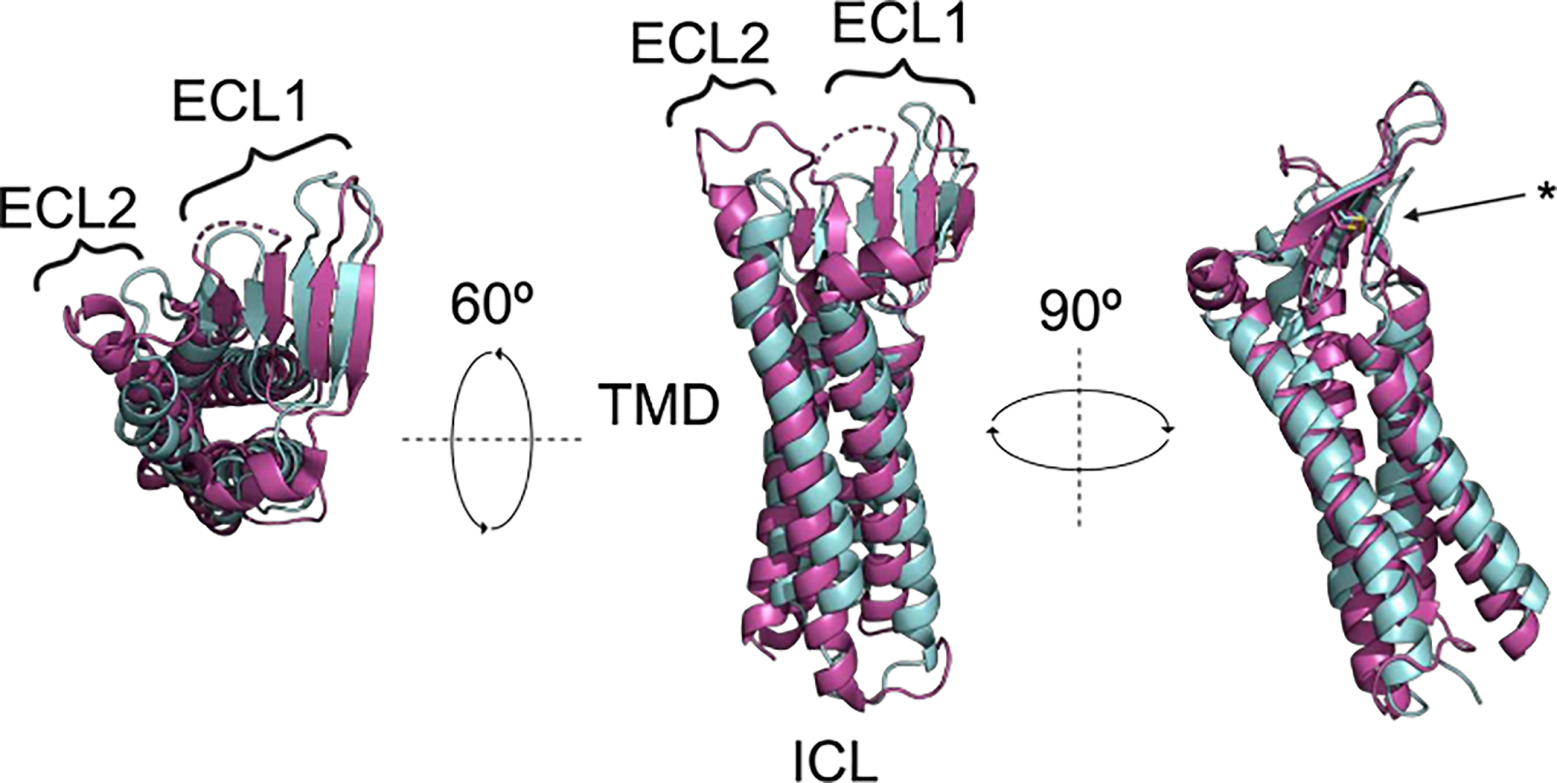
Superimposition of the crystal structure of Claudin-15 (PDB 4P79; Magenta)^131^ and the AlphaFold model of PMP22 (AF-Q01453-F1-v6; Cyan) (RMSD = 5.985 Å). Both proteins form a tetraspan α-helical transmembrane bundle. Two extracellular loops between transmembrane helices 1–2 and 3–4 form a β sheet structure composed of 4–5 strands. The conserved disulfide bond in ECL1 is modeled with sticks and indicated with an asterisk. Dashed strands indicate unresolved regions in the Claudin-15 crystal structure. Not depicted are the intracellular N- and C-termini of Claudin-15.Abbreviations: ECL, extracellular loop; TMD, transmembrane domain; ICL, intracellular loop.

Based on the claudin structures available in the mid-2010s, a hybrid homology/ROSETTA model of PMP22 was developed^140^ (Figure 12). This model has been useful for explaining experimental results for PMP22. When AlphaFold emerged a decade later,^141^ it predicted a structure for PMP22 very similar to the homology/ROSETTA model. The RMSD between them is about 1.5 Å, with the superimposed structures depicted in Figure 12.

**Figure 12:**
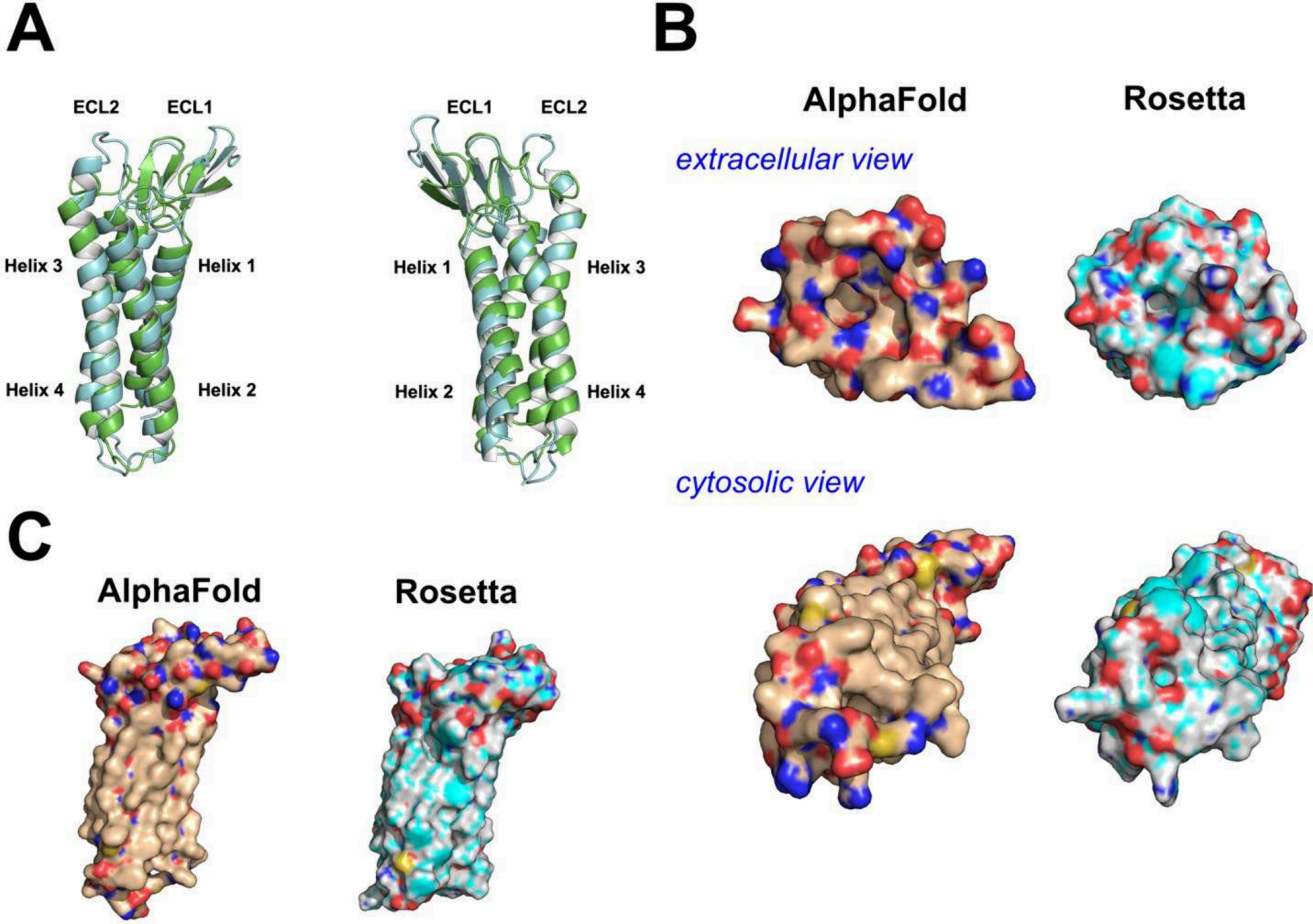
Homology/Rosetta vs AlphaFold Models for PMP22. (A) Comparison between the AlphaFold model (cyan, AF-Q01453-F1-v6) and 2014 Rosetta homology model (green) of PMP22.^140^ Both models largely agree (RMSD = 1.822 Å), differing primarily in the predicted lengths of transmembrane helix 3, positioning of the extracellular loops, and size of the inner cavity visible in the space filling models from the extracellular perspective.

Some other proteins are in the gray zone in terms of whether they are PMP22 homologues or not, but (among these) the experimental structures of AMPAR-regulating GSG1 family members^135,142^ are uniform in supporting the notion that most do indeed include the same central fold for the transmembrane and extracellular 5-strand β sheet domains. Additional proteins that are predicted by AlphaFold to share the claudin fold with PMP22 include the CLP-24/TMEM204 adherens junction protein, Clarin/USH3A, and BCMP1/TMEM47, which plays a role in assembly of tight and adherens junctions. Two other proteins, the TMEM202 protein expressed in male germ line stem cells, and NKG7 (natural killer cell protein 7) are predicted by AlphaFold to share a TM domain similar to that of PMP22 and are predicted to have an extracellular β-domain, but are not predicted to maintain all five strands of the canonical claudin fold. Examination of the structures of the various claudin fold-containing proteins in juxtaposition with what is known about their functions suggests that the 2-domain claudin fold serves as a scaffold upon which considerable structural variety can be engineered by nature (Figure 13).

**Figure 13:**
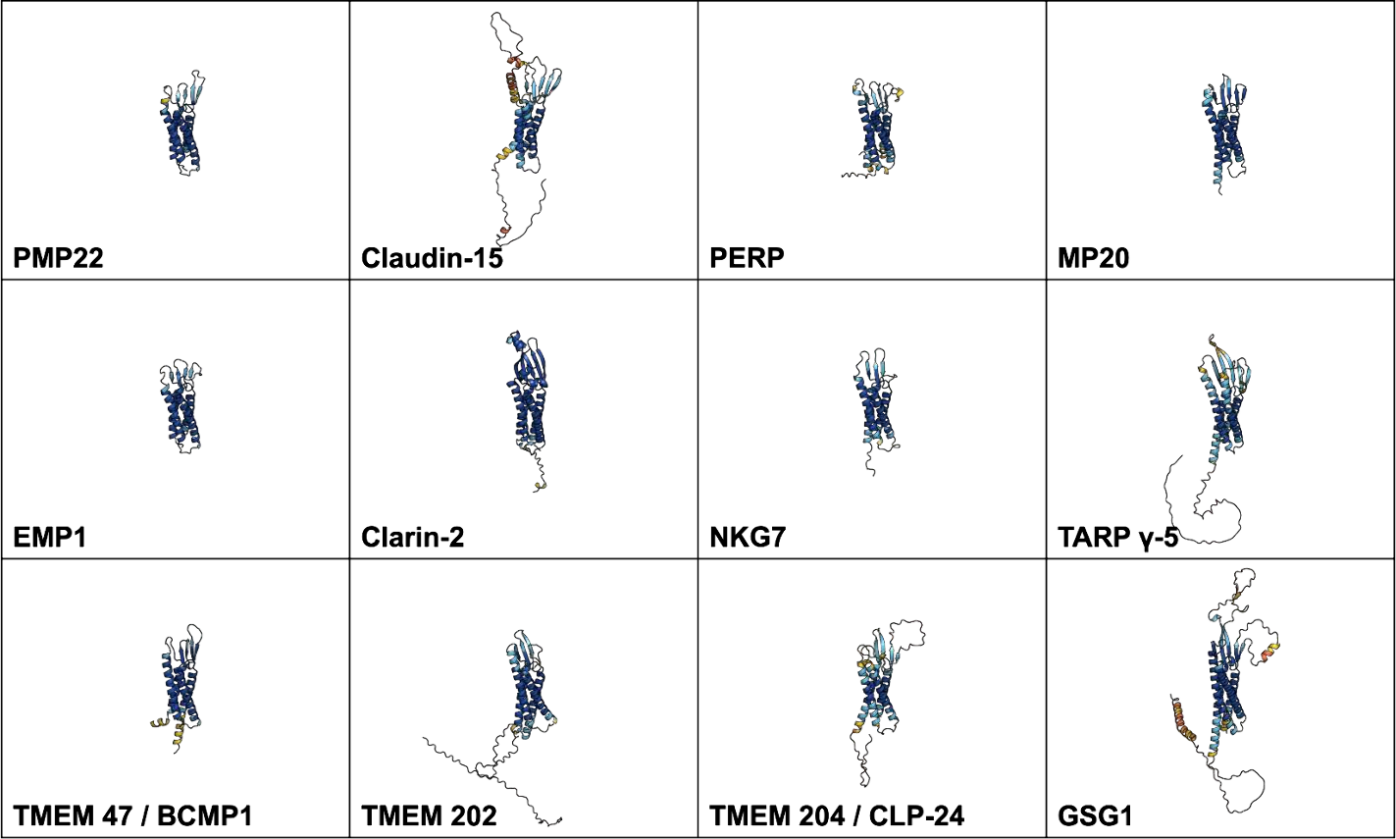
AlphaFold models^141,143^ of PMP22 and related tetraspan helical membrane proteins. All depicted proteins share the core tetraspan helical bundle and extracellular β sheet domain but exhibit substantial variability in the length and structure of their intracellular extensions and extracellular loops. Residues are colored according to predicted local distance difference test (pLDDT) values calculated by AlphaFold (Confidence Levels: Very High, Dark Blue; High, Light Blue; Low, Yellow; Very Low, Orange). AlphaFold models used: PMP22, AF-Q01453-F1-v6; Claudin-15, AF-A0AAA9SFU3-F1-v6; PERP, AF-Q96FX8-F1-v6; MP20, AF–P55344-F1-v6; EMP1, AF–P54849-F1-v6; Clarin-2, AF-A0PK11-F1-v6; NKG7 AF-Q16617-F1-v6; TARP γ-5, AF-Q9UF02-F1-v6; TMEM 47/BCMP1, AF-Q9BQJ4-F1-v6; TMEM 202, AF-A6NGA9-F1-v6; TMEM 204/CLP-24, AF-Q9BSN7-F1-v6; GSG1, AF-Q2KHT4-F1-v6.

The orientation of the beta domain with respect to the bilayer plane and normal is seen to be variable. Also, while the β-domain of PMP22 is predicted to be relatively flat, for some of other proteins in this family the domain is highly curved or irregular. The β-domain itself can serve as an anchor point for extended extracellular loops, as in the case of the TARPs and GSG1 proteins, which bind laterally to the AMPAR in the membrane, with large loops extending from the ends of β-strand pairs, contacting the receptor to profoundly modulate its function.^135^ In the case of PMP22, residue Asn41, located on the second β-strand of ECL1, serves as the attachment point for an N-linked oligosaccharide. The TMD helices are also seen to be dramatically extended for some claudin domain proteins, both into the extracellular space and into the intracellular space (Figure 13).

Tetraspan membrane proteins that are not believed to be related to PMP22 by homology or structure include the connexins, the tetraspannin family proteins, the MAL protein, occludin, MPV-17, peroxisomal membrane protein 22, ductin, plasmolipin, cornichons, and the proteolipid protein (PLP). The apparent lack of relationship between PLP and PMP22 is particularly interesting as PLP is the major adhesive protein in compact myelin of the CNS, where it is believed to play the central adhesive role that P_0_ plays in the PNS.^144^ PMP22 is expressed at only low levels in the CNS and PLP is expressed only at low levels in the PNS. In addition to the fact that PLP has a different conformation than PMP22 and lacks sequence homology, PLP is not glycosylated but is heavily lipid-modified.^144^

## 5 PMP22 Protein: Structure

The first six residues of human PMP22, MLLLLL-, represent the start of transmembrane segment 1 (TM1) (Figure 14). Even for a membrane protein, it is unusual to see such a uniformly hydrophobic N-terminus, a trait that PMP22 shares with the epithelial membrane proteins (EMPs) (Figure 10), but not with other claudin fold proteins. Seemingly, only the N-terminal amino group is responsible for anchoring the start of PMP22 at the hydrated membrane surface. EMPs also share the compact extracellular domain of PMP22, which is composed almost entirely of the 5-antiparallel strand β-domain with only short strand-connecting loops. As noted, the β-domain is fairly flat with its plane only moderately tilted with respect to the plane of the membrane. While not yet experimentally verified, the β-domain almost certainly contains a disulfide bond between Cys42 on the third β-strand and Cys53 on the fourth β-strain, (both in ECL1).

**Figure 14:**
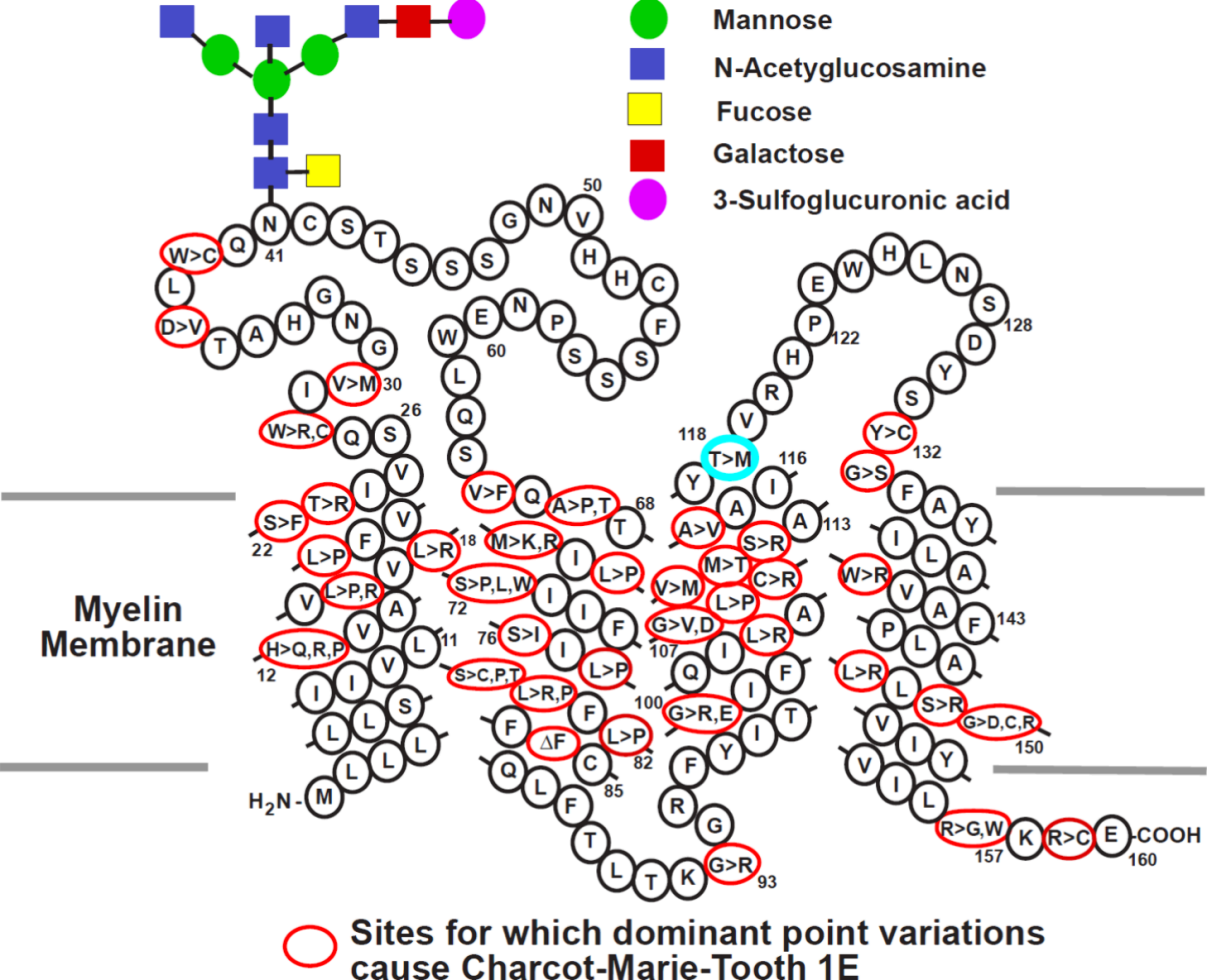
Topology diagram of human PMP22, providing the locations and identities of reported CMT1E variants based on the compilation provided in the 2024 version of the Human Gene Mutation Database (HGMD Professional version 2024.4).^120^ The site of the T118M variation that is found in roughly 1:100 of humans is highlighted in cyan.

Cys 42 is immediately adjacent to the site of N-glycosylation in PMP22, Asn41. Mature PMP22 that reaches the cell surface has, as its major (mature) glycoform, a branched oligosaccharide that serves as an HNK-1/L2 epitope.^145–148^ Canonically, it contains 11 pyranoside units with 3-sulfoglucuronic acid located at the end of its longest chain (7 sugars) starting from the Asn41 attachment point (Figure 14). In a fully extended conformation this anionic terminal pyranoside would extend roughly 35 Å from its attachment point on the β-domain of ECL2. However, some very preliminary modeling suggests that the dianionic terminal glycoside (3-sulfoglucuronic acid) may undergo favorable electrostatic interactions with positively charged residues in the PMP22 extracellular domain (ECD), such that this N-glycoside is oriented to form more of a cap to the bowl-like ECD rather than extending away from the membrane plane, antenna-like (Figure 15). It should be emphasized that this oligosaccharide, which was painstakingly isolated and analyzed by Kitamura et al.,^147^ was proposed to be the major oligosaccharide attached to PMP22 in myelin, but that there are many alternative (but as-yet-uncharacterized) glycoforms attached to Asn41.

**Figure 15:**
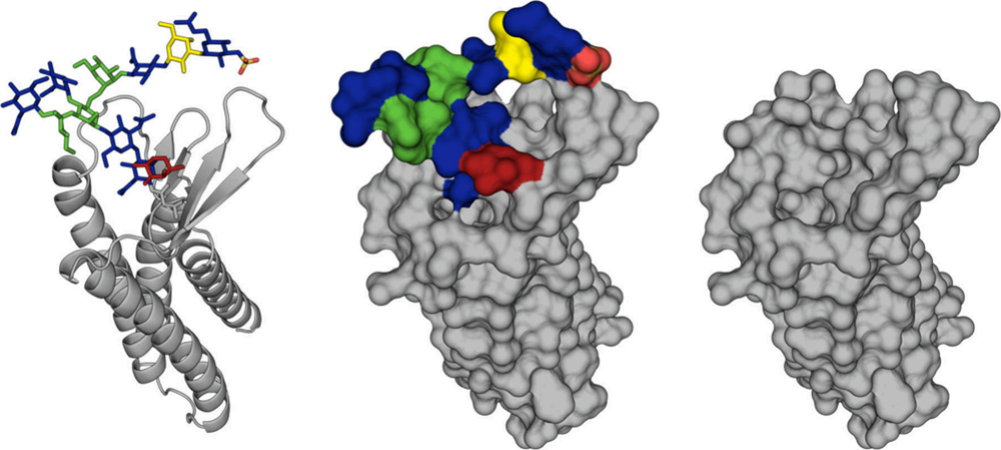
Complex HNK-1/L2 N-glycan modeled into the PMP22 AlphaFold model at residue N41. (Left) The complex HNK-1 glycan (HSO3(−3)­GlcA­(β1–3)­Gal­(β1–4)­GlcNAc­(β1–2)­Man­(α1–6)­[GlcNAc­(β1–2)­Man­(α1–3)]­[GlcNAc­(β1–4)]­Man­(β1–4)­GlcNAc­(β1–4)­[Fuc­(α1–6)]­GlcNAc) is modeled as sticks. The space filling model of PMP22 is also shown both with (Center) and without (Right) the glycan. Monosaccharides within HNK-1 glycan are colored according to the Symbol Nomenclature for Glycans (SNFG): Blue, d-Glucopyranose (Glc) and d-Glucopyranuronic acid (GlcA); Red, L-fucopyranose (Fuc); Green, D-Mannopyranose (Man); Yellow, D-Galactopyranose (Gal). The HNK-1 glycan was modeled onto the PMP22 AlphaFold structure using GLYCAM-Web.^149^

ECL1 contains 34 residues, while ECL2—connecting TM3 and TM4—contains only 9 residues, which includes the short fifth beta strand that pairs with strand 1 from ECL1. The extracellular domain of PMP22 contains 4 histidines and 2 acidic residues. It is therefore not surprising that purified recombinant PMP22 has been shown to bind transition metal cations Zn­(II) or Cu­(II) with fairly high affinity, promoting higher conformational stability to PMP22 relative to the apoprotein, as assessed using purified recombinant PMP22 in model membranes.^150,151^ Whether such metal ion coordination is physiologically relevant and exactly which residues are involved in coordinating these ions is unclear. It is interesting but of unclear relevance that dietary deficiency of Zn­(II) in animals causes peripheral neuropathy.^152,153^

The short ECL2 loop provides the insertion site (between residues His125 and Leu126) for the 10-residue Myc epitope (-**EQKLISEEDEL**-) that is frequently used in conjunction with commercial anti-Myc antibodies in studies of PMP22 in model cells lines. This provides a convenient way of immunologically detecting PMP22. Insertion of this epitope at this ECL2 site appears to be reliably nondisruptive of the native structure, trafficking, or interactions of PMP22.^154,155^ Attempts to employ alternative epitopes or reporter proteins fused to either or both intracellular termini have led to mixed results.^154,156–161^ At least for fusions to the C-terminus, this may not be surprising since a variant form of PMP22 that extends its C-terminus into the cytosol by 9 residues causes an HNPP-like form of CMT.^162^ A welcome development is a recently developed synthetic antibody fragment, “COP-1”, that binds to human PMP22 with a K_D_ of 100 nM, forming a complex with a half-life of 5.8 min.^163^ This reagent also binds to the claudins and other PMP22 homologues.

The cytosolic loop that connects TM2 and TM3 is only 7 residues long and contains single lysine and arginine residues. Just prior to this loop in the lower end of TM2 is Cys85, which has been reported to be palmitoylated,^164^ a post-translational modification that alters PMP22-expressing epithelial cell morphology and motility.^164^ PMP22 also contains a CRAC motif at its C-terminus, which is sometimes associated with cholesterol binding.^165^ Indeed, Notterpek and colleagues showed that mutations in this motif disrupt cholesterol homeostasis in Schwann cells.^166^ TM4 is abruptly terminated by PMP22’s C-terminal residues -RKRE.

Interactive viewing of either the AlphaFold or homology models of PMP22 reveals two cavities. There is a shallow bowl located at the extracellular face of the protein, with hole in the bowl leading to an “inner crevice”, which is larger in the AlphaFold model than in the homology model (Figure 12B). Near the cytosolic water-membrane interface is another crevice (hard to visualize in a snapshot). In a small molecule screen,^167^ we have found compounds that bind to both sites.

There are reports in the literature that PMP22 may form hexamers or octamers in cells.^168^ In working over the past 20 years with purified protein following heterologous expression in *E. coli*, Sf9 cells, or HEK suspension cultures, we have not observed a tendency of PMP22 to form oligomers higher than dimers. Of course, this does not rule out the possibility that higher oligomers form under native conditions. In any case, a modest tendency of PMP22 to form dimers has been shown, even for purified protein,^155,169–172^ but the location of the dimer interface is not known. Perhaps surprisingly, there is data showing that the tendency of purified PMP22 to dimerize may be reduced when the protein is reconstituted into more native-like lipid compositions relative to detergent micelles.^172^ It has also been seen that certain disease mutants seem to be more prone to dimerization than WT.^172^

PMP22 was first purified by Kitamura, Suzuki, and Uyemura in 1976 from PNS myelin following organic solvent extraction from myelin membranes and then purified in SDS micelles.^173^ A method for purifying PMP22 from native myelin into detergent micelles was later reported by Sedzik and colleagues,^93^ with methods for recombinant (*E. coli)* expression and micellar purification of the protein later being reported much later, involving DPC micelles^171^ or alkyl glycosides such as β-dodecylmaltoside.^174,175^

## 6 The PMP22 Protein Has an Affinity for Lipid Raft Domains

PMP22 was among the first multispan membrane protein that was quantitatively shown to preferentially partition into ordered membrane domains (colloquially known as “lipid rafts), as determined in giant plasma membrane-derived vesicles (GPMVs).^176^ GPMVs are derived by blebbing liposomes from mammalian cells, which are then cooled to below 37°C to induce a transition in the derived vesicles from containing only very small and dynamic ordered domains to large and stable raft-like domains that can be stained and visualized via confocal fluorescence microscopy. It was seen that WT PMP22 partitions into such raft domains with a partitioning coefficient of 0.78 (meaning that only 22% is seen in the surrounding disordered phase) in GPMVs from either HeLa or Schwann cells expressing PMP22. It was observed that this pronounced phase domain preference was not dependent on N-glycosylation or palmitoylation. On the other hand, several CMT1E variant forms of PMP22 known to destabilize the 3D structure of the protein reversed its phase preference to favor partitioning in the disordered phase.^176^ This suggests that the phase preference of PMP22 is embodied in its native conformation, although what structural traits set folded PMP22 apart from multi-span membrane proteins that prefer the disordered phase (even when folded) are not known. Given that much of the PMP22 produced during myelination appears to be destined for cholesterol and sphingolipid-rich compact myelin membranes, it is perhaps unsurprising that WT PMP22 prefers raft-like membrane domains. Possibly related to this fact are proteomic observations from HEK and Schwann cells that PMP22 interacts with a number of enzymes of cholesterol and sphingolipid biosynthesis, respectively.^177,178^

## 7 Introduction to the Possible Normal Functions of PMP22

No single function of PMP22 is well-understood. However, there is much evidence that PMP22, despite its small size and compact structure, is a multifunctional protein. We summarize its known slate of possible functions below, noting that some may overlap.

### 7.1 Possible Normal Functions of PMP22: Adhesive Role in Compact Myelin

The best-documented location of PMP22 in mature Schwann cell/myelin units is in compact myelin,^86–89^ where the other major proteins are P_0_ and (in the myelin membrane) and MBP^84^ (in the cytosol). While P_0_ is believed to be the major adhesive protein of compact myelin, it is very likely that PMP22 also plays an important supporting role (Figure 6). It is known^78,90,91^ that PMP22 and P_0_ undergo *cis* binding in the same bilayer and also likely work together to generate the *trans* adhesion between juxtaposed bilayers across extracellular space in compact myelin, although as yet the exact stoichiometry of this supercomplex has not been determined. While knockout of PMP22 does not obviate myelin formation, there is much evidence that the structural proteins of myelin—especially P_0_ and PMP22—exhibit some degree of functional redundancy in terms of supporting myelin organization and adhesion.^179–181^

Human PMP22 shares the same N-oligosaccharide moiety, the HNK-1/L2 epitope, as both P_o_ and the myelin-associated glycoprotein (MAG) (Figure 14).^95,145,147,148,182–184^ Moreover, there is evidence that PMP22 and P_0_ bind to each other in both *cis* (within the same bilayer) and *trans* (kissing interaction spanning juxtaposed bilayers in the myelin layers) modes.^78,90,91,185^ It seems possible that the HNK-1 oligosaccharide could play a Velcro-fuzz-like adhesive role, with the Velcro-hook-like countersurface being provided by itself, by P_0_, and/or by MAG to form trans heterocomplexes that span the extracellular space between layers of myelin membranes.^95,182,186^ There are also glycolipids found in SCs that have HNK-1 oligosaccharide headgroups, and it cannot be ruled out that they could also play an adhesive role.^187,188^ Indeed, some evidence that HNK-1 plays a role in P_0_-P_0_ interactions has been presented,^189^ although for the case of PMP22-P_0_ coimmunoprecipitation experiments do not support an avid HNK-1-dependent interaction.^90^ Future studies are required to determine the extent to which the PMP22 N-glycoside is involved in adhesion or other functions. These studies may be aided by the recent chemoenzymatic syntheses of the HNK-1 oligosaccharide^190,191^ and by compounds suggested to mimic this glycoform.^192,193^

Additional evidence that PMP22 plays an adhesive and possibly organizing role in compact myelin is provided by Mittendorf et al.,^194^ who purified human PMP22 following *E. coli* expression and reconstituted the protein into lipid bilayers. While classical spherical multilamellar vesicles were generated in lipid-only control experiments, it was seen by negative stain EM, cryo-EM, and cryo-electron tomography (cryo-ET) that PMP22-containing mixtures spontaneously formed assemblies that are in some regards strikingly myelin-like. The PMP22-containing vesicles flatten and stack (Figure 16). The stack then wraps, hotdog bun-like, around a central tubular vesicle to generate “myelin-like assemblies” (MLAs). This intrinsic ability of PMP22, even without its native N-glycoside, to induce flattening and stacking of vesicles is consistent with an adhesive role for the protein in myelin. Unstable CMT1E mutant forms of PMP22 were unable to support MLA formation. It is interesting to note that the MLAs described in this work closely resemble primitive nonspiraled forms of myelin seen in copepods, tiny crustaceans.^195^

**Figure 16:**
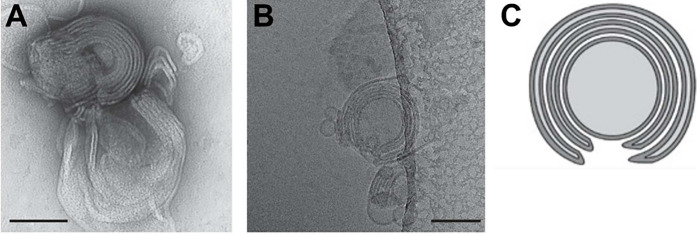
Purified recombinant PMP22 forms myelin-like assemblies when reconstituted in 4:1 POPC/ESM vesicles. The myelin-like assemblies were visualized by (A) negative stain and (B) cryo-EM. Scale bars (all panels), 100 nm. Based on data such as seen in these panels and cryo-electron tomography, a model (C) for the “myelin-like assemblies was proposed. Reproduced with permission from Figures 1B, 2D, and 3F of Mittendorf et al. 2017.^194^ Copyright 2017 The American Association for the Advancement of Science.

Further evidence that PMP22 may play an adhesive role in myelin formation is provided by observations of “intracellular myelin-like figures” IMLFs when PMP22 is overexpressed in model cell lines (Figure 17).^159,196,197^ These sometimes appear in electron micrographs as membrane whorls that form in the cytosol.

**Figure 17:**
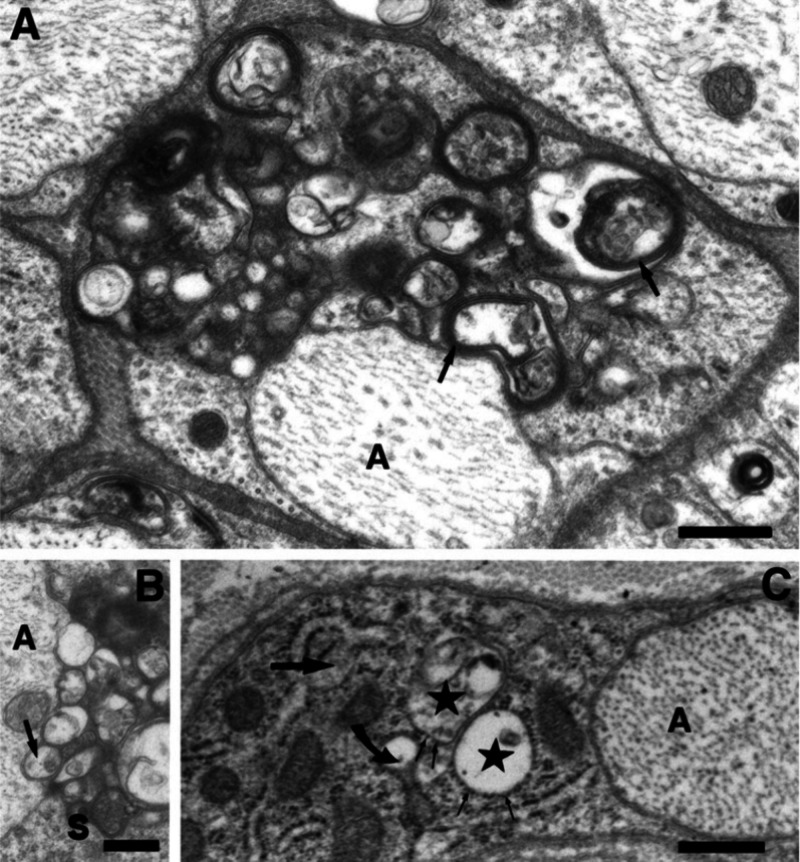
Electron micrograph of dysmyelinated nerves from C3 rats showing (A) intracellular myelin-like figures (IMLFs, arrows) that are not associated with the axon-A. (B) Vesicular structures (arrow) that are associated with adaxonal surface, and (C) abnormalities in the ER. Scale bars (all panels), 0.5 μm. Caption adapted and figure reprinted with permission from Niemann et al, 2000 (Figure 2).^197^ Copyright 2000 Society for Neuroscience.

Much work remains to elucidate interactions of PMP22 with itself and with P_0_ in compact myelin, but there remain good reasons to suspect that PMP22 plays an important role in organizing and adhering the spiral double membrane layers of compact myelin.

### 7.2 Possible Normal Functions of PMP22: Formation of Junctions in PNS Myelin

As noted earlier, the Schmidt–Lantermann incisures (SLIs) of PNS myelin include a series of gap junction channels through the surrounding spiraled layers of myelin and that are also the sites of tight junctions and adherens junctions (Figures 2B and 6).^60,62^ Gap junction channels are formed when pairs of connexin hexamers on apposed membrane bilayers form a 12-mer with a pore that spans both bilayers to connect the cytosols on each end of the pore. Connexin-32 is particularly important in PNS myelin, with variations impacting its sequence being the cause of CMT1X.

PMP22 is homologous to the classic tight junction-forming family of proteins, the claudins. Through *trans* (kissing) interactions between claudins on adjacent membranes, tight junctions (TJs) serve both to provide adhesion between adjacent epithelial cells (or adjacent layer of myelin) and to provide a fence-like barrier between apical and basolateral membrane domains.^132,198,199^ They may also be organized to form “paracellular channels” that enable selective diffusion of ions or other small molecules, not across the membrane, but rather between the apical extracellular space and the basolateral extracellular space.^65,66^ Notterpek and co-workers have shown that exogenously expressed PMP22 can be found at the tight junctions of model epithelial cell lines.^200,201^ While not yet directly visualized in SLIs or associated TJs, it is possible that PMP22 plays a role in forming and/or stabilizing tight junctions between spiraled layers of membranes in myelin. This is particularly the case in light of a recent study by Moss and co-workers of CMT1A and HNPP mice showing that, especially for the former, there is pronounced structural disruption of the SLIs.^202^

Adhesion junctions to some degree resemble tight junctions, serving as adhesive junctions on the apical membrane domain, parallel to and not far from the tight junctions. While the proteins, including E-cadherin, involved are mostly different from those for TJs, their fence-like organization and adhesive paracellular barrier functions are reminiscent. As for the case of tight junctions in PNS myelin, PMP22 has not yet been directly observed in myelin adherens junctions. However, Moss et al. have provided evidence for a possible role of PMP22 in their structure and/or function.^202^ This may be related to an observation by the same laboratory that the juxtaparanodal and paranodal domains of myelin in C3 (CMT1A model) mice are disorganized, resulting in defective axon/Schwann cell interactions.^202^

Finally, Guo et al., 2014 ^203^ showed that PMP22 deficiency in HNPP mouse model disrupts multiple cell–cell junction complexes in peripheral myelin, leading to increased myelin permeability and impaired nerve conduction without gross demyelination.

### 7.3 Possible Normal Functions of PMP22: Cholesterol Transport and Homeostasis

Schwann cells expand their membrane surface area during myelination by a factor of several thousand,^56^ with cholesterol representing a little over 1/3 of its membrane lipids.^96^ While cholesterol is essential for SC function,^97,98^ dietary cholesterol cannot cross the blood-nerve barrier. Some of the cholesterol used by SCs is recycled from damaged SCs or other PNS tissue, but most cholesterol is synthesized in SCs and then trafficked through the secretory pathway to the plasma membrane and hence into myelin.^98^ PMP22 knockout mice and TrJ mice exhibit intracellular retention of cholesterol,^166^ while in CMT1A SCs, PMP22 and cholesterol tend to accumulate in endosomes and lysosomes.^98,204^ Moreover, there is evidence that not only does PMP22 preferentially associate with lipid rafts, but it may also play a critical and central role in facilitating trafficking of newly synthesized cholesterol to the cell surface and into myelin membranes.^166,205^ PMP22 modulation of cholesterol trafficking may involve direct binding, possibly involving its CRAC motif in concert with its association with P_0_.^166^

WT PMP22 preferentially associates with lipid rafts,^176,206–208^ but CMT1E variant forms of PMP22 show an opposite preference for disordered phase membranes.^176^ Moreover, WT PMP22 is seen to increase the size of lipid raft-like ordered membrane domains present in HeLa cell-derived giant plasma membrane-derived vesicles (GPMVs) relative to both control and to analogous conditions when the L16P CMT1E mutant form of PMP22 is present.^176^ Compounds were recently discovered that both alter lipid raft formation and the partitioning of PMP22 into lipid rafts.^209^ These compounds may be useful tools in the study of PMP22/lipid raft relationships.

PMP22 has also been proposed to interact with a cholesterol export protein found in SCs, namely, ABCA1. This transporter may play a role in repair of damaged myelin by SCs, where cellular and membrane debris may need to be cleared out in preparation for remyelination.^205,210^ PMP22 may also help regulate cholesterol biosynthesis.^211^ How it does this is not understood, although it may be significant that of 56 proteins reported to interact with WT PMP22 in an HEK cell-based proteomics study, seven have functions related to cholesterol biosynthesis or homeostasis.^177^

### 7.4 Possible Normal Functions of PMP22: Modulation of the Functions of Ion Channels or Other Important Membrane Proteins

PMP22 homologues of the TARP and GSG1 protein families modulate the AMPAR receptor and other channels by laterally binding to them in the membrane and modulating their functions via specific interactions of extracellular loops anchored to their β-sheet domains.^135^ PMP22 may play an analogous role in modulating the functions of one or more other proteins. Intracellular Ca­(II) levels are elevated in CMT1A, and it has been reported that PMP22 activates P2X7 channel activity.^212,213^ Evidence has also been presented that PMP22 interacts with STIM1 to activate SOC Ca­(II) channel activity, one subunit of which is the transient receptor potential TRPC1 calcium channel.^214^

The closest family of homologues to PMP22 is the epithelial membrane protein family (EMP1, EMP2, and EMP3). This is an important family of membrane proteins, playing roles in certain forms of cancer, for example.^126,215^ However, the functions of the EMP proteins have remained mysterious. It is therefore significant that Li et al. recently published a study of EMP1 that shows it to play a major role in mitigating ER stress by binding to ceramide synthase 2 to inhibit its function, the production of dihydroceramides. These lipids induce protein aggregation and susceptibility of hematopoietic stem cells to injury by ionizing radiation, as occurs in radiotherapy.^216^ The interaction of EMP1 with the synthase was proposed to involve its compact extracellular β-domain. This mode of action seems not very different from the general picture for how the TARPs and GSG1 regulate the AMPAR.^135^ Besides possibly regulating Ca­(II) influx into the cytosol, could it be that PMP22 also regulates cholesterol and/or biosynthesis of certain sphingolipids by direct binding to lipid biosynthetic enzymes? This possibility is supported by studies suggesting that PMP22 modulates and/or interacts with a number of enzymes involved in cholesterol and sphingolipid biosynthesis.^177,178,211^ Indeed, sphingolipid defects have been observed in CMT1A.^217–220^

### 7.5 Possible Normal Functions of PMP22: Regulation of the Schwann Cell Cycle and Differentiation

Schwann cells undergo an unusually complex developmental program that can lead to both myelinating and nonmyelinating states (Figure 3). Moreover, myelinating Schwann cells can undergo dedifferentiation, as occurs, for example, in the process of PNS nerve repair (Figure 8).^47,48,108–110,221^ There is also much evidence that PMP22 plays roles, sometimes seemingly contradictory, in SC growth arrest, proliferation, regulation of the cell cycle, apoptosis, and differentiation.^5,7,211,222–230^ However, it is difficult to parse out normal from pathogenic functions, related to these processes. Some of these possible functions for PMP22 are largely inferred from the exogenous expression in cultured model cell lines. It seems quite possible that some of these roles may be specific to certain states of SC differentiation. In all cases, the molecular mechanisms by which PMP22 signals or otherwise impacts the fundamental cell states and processes remain to be determined.

### 7.6 Possible Normal Functions of PMP22: Cell Spreading and the Cytoskeleton

There is evidence that PMP22 can play an important role in cell spreading of epithelial cells, a process closely associated with actin polymerization,^164,200^ and that seems to require N-glycosylation^231^ and ECL2 ^200^ of PMP22. Cell spreading is related to wound healing. It has been argued that PMP22 is unlikely to play an analogous role in promoting myelin spiraling (radial expansion) led by an extending lamellipodia tongue during myelin formation.^232^ However, it is possible that a spreading-like phenomenon could occur during growth-related myelin segment lengthening. The RhoA GTPase has been identified as a potential PMP22 interactor in proteomics studies^177^ and may modulate PMP22’s role in this process.^233^ PMP22 may also play a role in forming or maintaining the actomyosin belt that is closely associated with tight and adherens junctions in epithelia and at the junctions of the SLI in PNS myelin (Figure 6).^201,202,206,234^ Determination of how PMP22 performs these roles and whether direct interactions of PMP22 with actin or related molecules occur will require further study. There is also evidence that the level of PMP22 in myelinating SCs alters the cytoskeleton of associated axons through unknown mechanisms.^235–237^

### 7.7 Possible Normal Functions of PMP22: Supporting Interactions of SCs with the Basal Lamina

A prerequisite to myelination by Schwann cells is the formation and maintenance of a basal lamina (basement membrane, see Figure 4).^50^ This process involves the construction and transport of key substituent proteins, such as collagens and laminin from the cytosol to the extracellular space, where they are organized and tethered to the plasma membrane through interactions with integrin α/β heterodimers.^238^ There is evidence that PMP22 expression levels alter integrin levels and function.^239,240^ In some cases—such as β4, α2, α6, and α7—PMP22 may bind to integrin subunits.^240–242^ These interactions likely play key roles in the construction of the basal lamina, and may possibly persist during and after myelination, playing a central role in the process by which PMP22 helps to provide mechanical stability to peripheral nerves.^243–245^

The L2/HNK-1 oligosaccharide/epitope that adorns ECL1 of PMP2 has been shown to bind a key polymer of the extracellular matrix,^246^ laminin, suggesting a direct, but as yet untested, PMP22-laminin interaction. Laminin’s best-known cell surface anchors are the integrins, including the α_6_β_4_ integrin believed both to bind PMP22 and to play an important role in SC basal lamina formation.^242^

Reductions in levels of key molecules of the basal lamina are seen in the sciatic nerves under conditions of either WT overexpression or expression of TrJ PMP22.^211^

## 8 The PMP22 Protein Traffics Inefficiently in Cells, Even under Healthy Conditions

There is much evidence that correct folding and trafficking for any given protein in mammalian cells can often be much lower than 100%, even under healthy conditions.^247^ A classic example is the cystic fibrosis transmembrane regulator (CFTR), an ATP-modulated chloride channel. WT CFTR appears to successfully fold and traffic to the plasma membrane with an efficiency of roughly 50% (see review in ref 247). Based on this and other examples, we long ago examined this phenomenon from an energetic perspective and hypothesized that for proteins such as CFTR the energetics of correct assembly and trafficking versus misfolding and mistrafficking—whether under thermodynamic or kinetic control—are surprisingly evenly matched.^248^

WT PMP22 appears to be CFTR-like in terms of the inefficiency of its folding and trafficking. In early work,^161,249^ as later confirmed,^150^ WT PMP22 was reported to fold and traffic to the plasma membrane in mammalian cells (both SC and non-SC cell lines) with an efficiency of roughly 20%. One objection that could be raised to this work is that some of the studies leading to this conclusion were carried out using model cell lines that were transiently transfected with *PMP22* under the control of strong promoters that result in high-level PMP22 expression. This might oversaturate the protein-folding quality control system, resulting in inefficient folding and trafficking. However, single-cell analysis of MDCK and HEK cells transfected with PMP22 has revealed that even in cells that express only low levels of PMP22, the surface trafficking efficiency is less than 50%.^250^

Studies of the folding kinetics of purified WT PMP22 in detergent micelles led to the conclusion that the folding and unfolding kinetics of PMP22 are very slow, requiring over an hour either way to reach completion.^170^ It is therefore possible that its folding rate in cells is slow such that misfolding and/or degradative pathways readily compete. At the same time, it was found that, at least in micelles, WT PMP22 is only a marginally stable protein,^170^ consistent with the notion that the low-energy difference between the folded and unfolded forms of PMP22 may also play a role in dictating its <100% folding/trafficking efficiency in cells.

When thinking about the stability of PMP22 in cells it is important to recognize that organelle-to-organelle lipid compositions in mammalian cells vary. Consider that the membranes at the site of initial PMP22 biogenesis, the endoplasmic reticulum (ER), have the lowest cholesterol concentration in the cell, ca. 5 mol %.^251^ Cholesterol concentrations increase to 10–15 mol % in the Golgi and then to >20% at the plasma membrane.^251^ It is known that the cholesterol content of compact myelin is 37–40 mol %.^96^ We hypothesize that the stability of correctly folded PMP22 will be highest in cholesterol-rich raft-like membrane compositions such as those that appear to dominate compact myelin. If so, this would suggests that WT PMP22 is most unstable and therefore most vulnerable to unfolding, misfolding, and degradation early in the secretory pathway—in the ER. Then, when correctly folded PMP22 advances beyond ER quality control and traffics on to the Golgi, the plasma membrane, and finally into myelin, it likely is stabilized by the increasing levels of cholesterol and sphingolipids. Moreover, while the documented slow kinetics of PMP22 folding^170^ may initially be a hindrance to folding, the unfolding rate is also slow. Thus, once correctly folded, PMP22 may tend to persist in this state, a property of the protein that we hypothesize will become more pronounced as PMP22 makes its way into myelin membranes. An interesting direction for future study will be to determine whether correctly folded PMP22 in the membranes of compact myelin is effectively kinetically trapped in its thermodynamically most favored state.

## 9 PMP22 Is Initially N-Glycosylated in the ER and Can Be Degraded via the Proteasomal Pathway

The initial integration of PMP22 into cell membranes has never been studied. However, proteins of both the Sec61 and EMC membrane protein integration pathways, as well as certain ribosomal subunits, were observed among 56 PMP22-interactive proteins in identified in a proteomics study in transiently transfected HEK cells that was weighted toward detection of interactions of PMP22 that occur early in the secretory pathway.^177^ PMP22 was also seen to interact with the ZMPSTE24 protease, which is known to degrade membrane proteins that are misintegrated into the membrane during or just after translation.^252,253^

Immediately after translation and integration into the ER membrane, PMP22 is N-glycosylated by the oligosaccharyltransferase (OST) complex. It has been shown that it can be glycosylated by either of the (alternative) catalytic subunits STT3A or STT3B.^177^ This initial glycosylation event serves as a ticket for PMP22 to engage with the calnexin cycle, which is coupled to the ER-associated degradation pathway (ERAD).^254,255^ It is likely that PMP22’s putative disulfide bond forms shortly after biogenesis, though this event is not tightly linked with N-glycosylation.^177^ PMP22 that folds correctly is recognized as such by proteins of the calnexin cycle or related ER-based folding sensors, resulting in modification of its N-glycoside, which subsequently serves as a ticket enabling PMP22 to exit the ER and traffic to the Golgi (Figure 18).^255,256^

**Figure 18:**
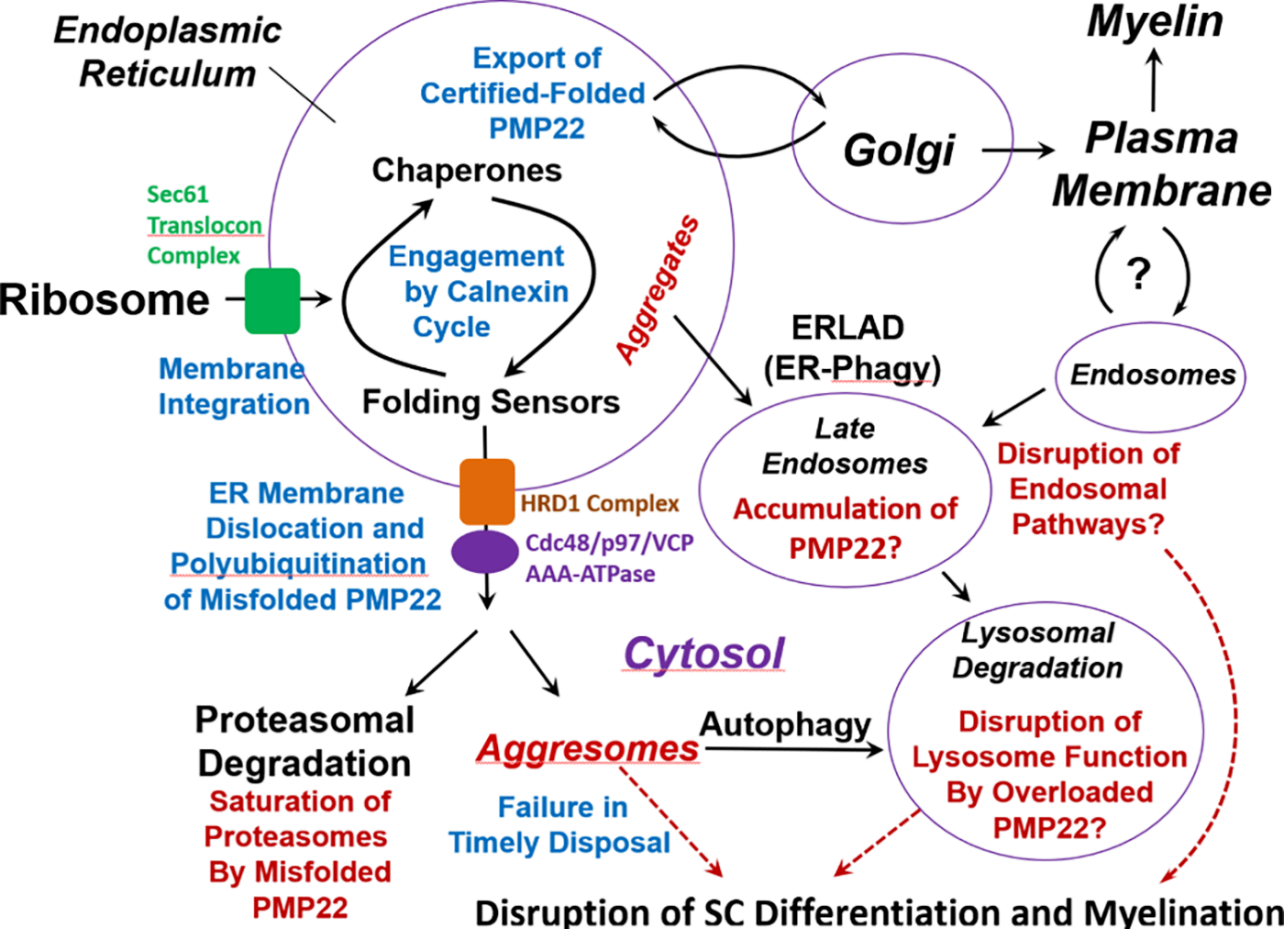
Known, likely, and possible trafficking pathways for PMP22 in myelinating Schwann cells.

PMP22 that reaches the Golgi is subject to further additions and modification of its core N-linked oligosaccharides to generate its final mature glycoform (see Figure 14) that can serve as the HNK-1/L2 epitope.^145,147^ Core-glycosylated PMP22 runs around 22 kDa in SDS-PAGE, explaining where the “22” of PMP22 derives from.^89^ At least one study indicated that under physiological conditions mature PMP22 is retained in the Golgi until myelination is activated.^161^ Total and surface expression levels of PMP22 are low in Schwann cells until myelination is activated.^87,89,161,257^ Under conditions of myelination, correctly folded and fully assembled PMP22 traffics from the Golgi to the plasma membrane and into myelin (Figure 18).

For nascent PMP22 in the ER that is membrane-integrated but fails to promptly and correctly fold, enzymes in the calnexin cycle and/or related proteins of ER quality control modify its N-glycoside, this time to generate a ticket for the ERAD pathway, leading to extrusion from the membrane and proteasomal degradation (see review in ref,^255^ Figure 18).^249^ PMP22 that is glycotagged in this manner is delivered to an ER membrane-to-cytosol transport system that, in concert with possible ubiquitination of PMP22, delivers PMP22 to the proteasome for degradation. While WT PMP22 has only 2 lysine residues (both of which are cytosolic and near the membrane), it does appear to get ubiquitinated. From knock-down of ERAD pathway-associated ubiquitin ligases Hrd1/SYVN1 and gp78./AMFR, it is thought that they mediate targeting for degradation of certain CMT1E mutant forms of PMP22 but not WT.^249,258,259^ Whether these results are the final word on the involvement of gp78/AMFR in WT PMP22 proteasomal degradation is uncertain in light of a proteomics study that identified 56 possible WT PMP22 interactors in HEK cells, including the gp78./AMFR ubiquitin ligase along with a number of proteins of the calnexin cycle and components of ERQC and of ERAD.^177^ Under CMT1A-like conditions of PMP22 overexpression, excess and/or misfolded PMP22 is not chronically retained in the ER.^196,260^ It is interesting to note that the lectin chaperone calnexin is well documented to directly form a complex with both WT and CMT1E mutant forms of PMP22.^159,177,261–263,264^

In HEK cells, the N41Q mutation that eliminates N-glycosylation of PMP22 results in a remarkable 3-fold increase in surface trafficking of PMP22 from 20% to 60%,^177^ which points to the role of N-glycosylation in ER quality control mechanisms that retain the protein intracellularly until its folding is deemed correct and complete. Proteomics in HEK cells revealed both overlap of interactors of N41Q PMP22 with respect to WT and also many differences.^177^ Whether the surplus of N41Q PMP22 that reaches the membrane surface is properly folded is an interesting unanswered question.

## 10 The Proteasomes of Schwann Cells Are Specially Adapted

Schwann cells contain the canonical 26S proteasome found in most cell types. However, under inflammatory conditions, such as tollowing exposure to pro-inflammatory cytokines or in disease states including the demyelinating neuropathy Guillain-Barré syndrome, expression of immunoproteasome subunits is induced.^265,266^ The immunoproteasome alters proteolytic specificity to enhance the generation of antigenic peptides for loading onto MHC class II molecules, thereby facilitating immune recognition. Notably, Schwann cells are capable of MHC class II mediated antigen presentation, a function typically restricted to classical antigen-presenting cells such as macrophages and dendritic cells. This MHC type II presentation may further amplify local inflammation within diseased peripheral nerves.^265–267^

Beyond these immune-related functions, Schwann cells exhibit specialized regulation of the proteasome that reflects their unique biological roles. The ubiquitin–proteasome system is essential for Schwann cell dedifferentiation into a repair phenotype following nerve injury.^268^ In addition, Schwann cells express alternative proteasome activators, including PA200, which may support the high demand for ER-associated degradation (ERAD) as required to clear large quantities of myelin proteins such as P0 and PMP22 that are synthesized during active myelination.^269^ Unlike the canonical 19S regulatory cap, PA200 binds directly to the 20S core particle, promotes gate opening through the smallest central pore of any known activator, and functions independently of ATP hydrolysis or ubiquitin recognition.^270^

In models of CMT1B, where mutant P0 inhibits proteasome activity, PA200 expression is upregulated.^269^ Although this increase would conventionally be interpreted as a compensatory response aimed at enhancing proteasomal degradation, recent work demonstrates that the genetic deletion of PA200 paradoxically improves protein turnover, increases assembly of 26S proteasomes, reduces accumulation of polyubiquitinated proteins, and alleviates proteotoxic stress, resulting in improved myelination.^269^ These findings indicate that PA200 upregulation in CMT1B is maladaptive, leading to less optimal proteostasis. This raises the critical question of why Schwann cells induce PA200 expression under conditions in which it ultimately exacerbates the proteostasis dysfunction. Whether these observations can be extended to CMT1A or CMT1E conditions is not yet tested but seems plausible.

As described in the next section, when proteasomal capacity is compromised or maladaptively regulated in Schwann cells, alternative protein quality control pathways are engaged to maintain proteostasis. In this context, autophagy culminating in lysosome-mediated degradation has emerged as a critical parallel route for the clearance of myelin proteins, particularly PMP22, when the ubiquitin–proteasome system is saturated or impaired.

## 11 WT PMP22 Is Also Degraded via Autophagy in Lysosomes

Notterpek and co-workers led the way in showing that high-level expression of PMP22 in SCs saturates the proteasomal pathway, leading to accumulation of misfolded PMP22 in the cytosol^161^ and aggresome formation.^249,258^ Disposal of aggresomes is managed by activation of autophagy, which results in the transport of misfolded PMP22 as cargo to lysosomes, where at least some PMP22 is degraded.^161,258^ Additional evidence for this pathway was provided by proteomic data for WT PMP22 in HEK293 cell lines that suggests not only engagement of WT PMP22 with the calnexin cycle and the ERAD pathway, but also with the cytosolic BAG6-UBL4A-TRC35/GET4 complex.^177^ This complex has been proposed to play a role in monitoring the flux of proteins being targeted for proteasomal degradation, shunting excess protein to aggresomes when the proteasomal pathway is deemed saturated.^271,272^

There are many variations of autophagic pathways, including those staged directly from the ER rather than from the cytosol.^273,274^ These “ER-phagy” pathways lead to lysosomal trafficking and degradation, and overlap with the various pathways of ER-to-lysosome associated degradation (ERLAD).^275–277^ In studies of WT PMP22 overexpressed in rat Schwann cells, classical aggresome formation is not observed, but abundant PMP22 is seen in puncta that colocalize with late endosome and lysosome markers (see next section). This suggests that, in addition to forming cytosolic aggresomes that are eventually degraded via autophagic delivery to lysosome, misfolded PMP22 may sometimes also traffic directly from the ER to late endosomes and lysosomes via one or more ERLAD pathways. It seems clear that defective PMP22 is degraded not only by proteasomes, but also by lysosomes and that autophagy is sometimes involved (Figure 18). The fact that autophagy declines with age^278^ may contribute to the slowly progressive nature of CMT1A. Whether accumulated PMP22 in late endosomes or lysosomes reduces lysosomal function is not known but is a distinct possibility. It is believed that a number of neurodegenerative and other disorders are caused, in part, by problems with lysosomes due to proteostasis stress.^279–281^

Very little is known regarding the degree to which PMP22 that traffics to the plasma membrane is subject to endocytosis and, if so, what the partitioning ratio is between recycling to the plasma membrane following endocytosis versus trafficking on to late endosome and lysosomes for degradation (Figure 18). However, there is a report that PMP22 disrupts the Arf6-associated endosomal recycling pathway.^282^ Moreover, variant forms of the LITAF/SIMPLE and FIG4 proteins that result in the disruption of endosomes and/or the endosomal pathway are known to cause CMT1C and CMT4J, respectively.^283–288^

## 12 WT PMP22 Tends to Form Intracellular Inclusions

In human tissue from CMT1A/1E patients, CMT mouse models, Schwann cell/neuron cocultures, as well as in cultured Schwann cells and other model cell lines in which PMP22 is exogenously expressed, both WT and disease variant forms of PMP22 are seen to form intracellular puncta that are visualized via immunostaining and microscopy (Figure 19).^174,223,232,249,259,282,289–292^

**Figure 19:**
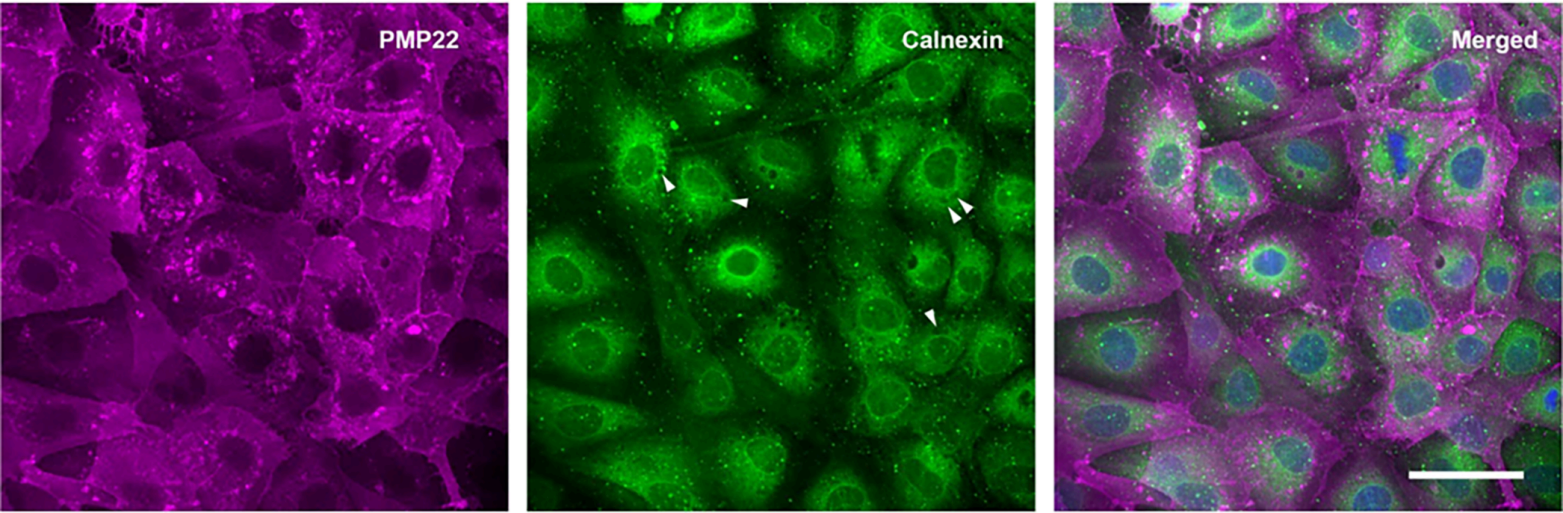
Confocal immunofluorescence micrographs of rat Schwann cells expressing human PMP22, highlighting the presence of PMP22 puncta. Large spaces in calnexin staining (examples highlighted by white arrows) are occupied by PMP22 puncta in the merged image (right), suggesting the PMP22 puncta are located near in the extremes of the ER, where they may be engaged by the ERLAD pathway. Scale bar is 50 μm (Sanders lab data).

These inclusions sometimes colocalize with organelle markers that indicate late endosomes and/or lysosomes. However, this is not always the case. Whether for any given example these puncta are dominated in composition by PMP22 or whether PMP22 is only one of many proteins present is not yet clear. Also unclear is whether the PMP22 in these puncta is aggregated, microdisperse, or something in-between. In the absence of additional data, we suggest that they are best described as PMP22 “inclusions” rather than aggregates. Also requiring additional study is the question of whether these inclusions represent stable assemblies or whether they are en route to degradation or some other fundamental transformation. The possible roles of PMP22-containing inclusions in CMT are discussed further in a later section.

A second form of inclusion that is sometimes associated with PMP22 are large assemblies that are located nearer to the plasma membrane.^159,197,282^ These assemblies sometimes have a spiraled appearance suggestive of myelin structure (Figure 17), in which case they are referred to as intracellular myelin-like figures (IMLFs).^159,196,197^ It is possible that these assemblies simply represent another mechanism by which cells manage an excess of PMP22.

## 13 Trafficking and Degradation Pathways Vary for Some CMT1E Variants

Two CMT1E variant forms of PMP22 have been subjected to much study over the years: the G150D and (especially) the L16P variant forms of PMP22, which, respectively, cause severe dominant forms of CMT, with G150D being considered a cause of DSS.^88,293–298^ While these are both rare variants in the human population, their profile in the CMT research literature relates to the availability of CMT mouse models that carry these variations. The Trembler-J (TrJ) mouse is based on L16P,^295^ while the Trembler (Tr) mouse is based on G150D.^294^ Clinically, mice and patients with either of these PMP22 variants do not present with exactly the same symptoms. Both L16P and G150D PMP22 have been shown to be thermodynamically unstable relative to those of WT.^150,151,299^ It has also been shown that G150D PMP22 is aggregation prone, unlike L16P or the WT protein.^154,155,169,300,301^

L16P/TrJ PMP22 has been reported to form long-lived complexes with the ER-resident lectin chaperone calnexin in sciatic nerves^159^ and is reported to accumulate in the ER-to-Golgi intermediate compartment (ERGIC) or the Golgi in cultured SCs.^155^ With the involvement of both calnexin and the RER protein, L16P PMP22 has also been reported to be polyubiquitinated in non-SC model cell lines, most likely by the HRD1/SYVN1 E3 ubiquitin ligase, and targeted for proteasomal degradation.^302^ L16P also is seen to form aggresomes, suggesting it saturates and/or inhibits proteasomes,^258,303–305^ possibly taking WT PMP22 with it via WT/mutant heterodimerization.^155,300,301^ The L16P-containing aggresomes are thought to be destined for engulfment by autophagosomes and trafficked to lysosomes for degradation.^306^ A proteomics study to identify L16P PMP22 interactors in HEK cells revealed that most ER quality control and ERAD pathway proteins identified as potential WT PMP22 interactors were also documented to be potential L16P interactors, but that L16P PMP22 may also interact with many additional proteins than WT.^177^ Moreover, an untargeted proteomics study using sciatic nerves from CMT mouse models indicated that, relative to WT PMP22, expression of L16P PMP22 upregulated expression of proteins involved in splicing, the proteasome, the lysosomal pathway, and of other proteins known to be overexpressed across a range of neurodegenerative disorders.^307^

G150D PMP22 inclusions have been observed in the ER,^160,300,308^ where they are engaged by calnexin,^261,262^ and this variant is also targeted for proteasomal degradation. Unlike L16P, G150D seems not to make it to the ERGIC or Golgi, and the RER protein seems not to be involved in its trafficking. Aggregation by this variant has been reported to involve disulfide bond formation.^262^ However, G150D PMP22 also seems to be targeted for proteasomal degradation and may saturate this pathway, leading to aggresome formation and shunting via autophagy to lysosomes.

## 14 Consequences of Complete Loss of PMP22 in Humans and Mice

While loss of a single allele of PMP22 leads to a mild disorder (see later section), elimination of both alleles has much more severe consequences.^180,309–313^ PMP22-null mice live only a few months and die of seizures. Only a single PMP22-null human has been described, a 7 year old boy with severe neuropathy.^309^ Examination of the PNS tissue from the boy and the mice revealed similar defects in myelination, with motor nerves being especially severely affected. Early stage myelination was delayed or defective, with the levels of expressed P_o_ and MBP being dramatically reduced. Small caliber axons were preferentially hypermyelinated, with the myelin that does form containing high diameter and often-loosely wound tomacula. Higher caliber axons were not myelinated at all or were thinly myelinated, often leading to axon degeneration and loss. The myelin that did form exhibited very short internodes. The tomacula were seen to be unstable and degenerated over a number of days, leading to demyelination. Schwann cell differentiation seemed to be mis-timed and deranged, with evidence for dedifferentiation occurring in some cases. Defects were observed in the basal lamina, which were accompanied by underexpression of integrin β4, a protein known to interact with PMP22.^242^ It was also seen that an axonal potassium channel involved in saltatory conduction, Kv_1.1_, was mislocalized, as also was a key adherens junction protein, E-cadherin, the latter normally being associated only with the SLIs. This suggests that tomacula formation and other myelin defects disrupt the paranodal septate junction that serves as a barrier between the juxtaparanodal region and the node in both axonal and adaxonal membranes.

A recent study of PMP22-null mice revealed major inner ear defects that very likely offer insights into hearing loss and problems with balance that afflict some CMT patients.^311^

## 15 CMT Disease Mechanisms Associated with PMP22

Demyelination of PNS axons is central to CMT1E, HNPP, and CMT1A. Electron micrographs of the sectioned ends of bundles of myelinated axons are revealing (Figure 20 and see ref 314). For severe CMT1E (DSS^314^), the axons are completely demyelinated, consistent with the extreme nature of severe CMT1E. For CMT1A, it is seen that some axons are normally myelinated, some are only thinly myelinated, and others are not myelinated at all, consistent with the notion that CMT1A is peripheral neuropathy of intermediate severity (compare Figures 20A and 20B). For HNPP, most axons appear to be myelinated normally, consistent with the generally mild nature of this disorder, but tomacula (high caliber, but loosely wound myelin segments, typically paranodal) are also seen (Figure 20C,D). For all three forms of CMT, problems with myelination result in axon degeneration, which leads to the manifestation of disease symptoms.^117^

**Figure 20:**
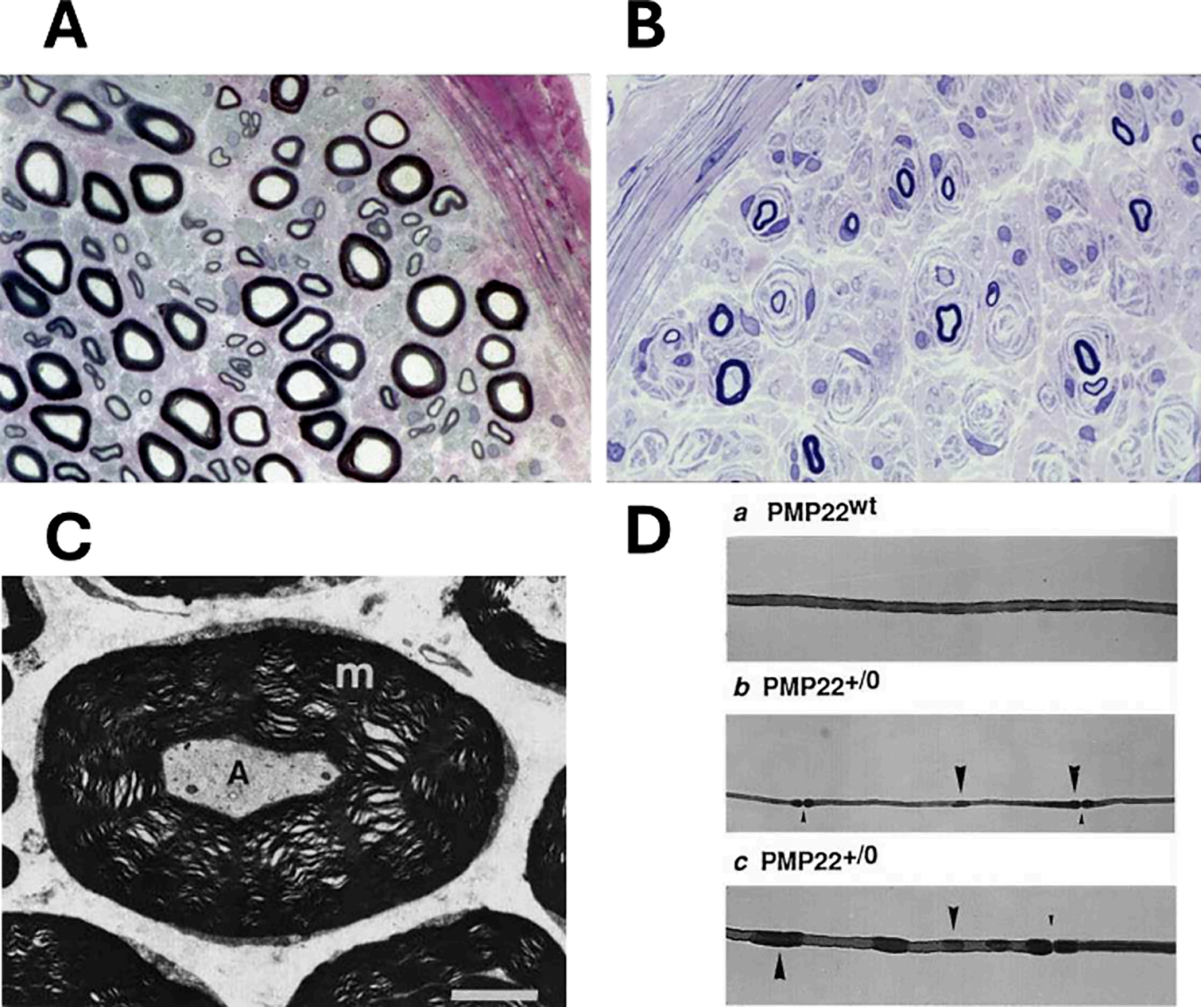
Healthy and unhealthy myelin. Panel (A) shows the cross section of a bundle of myelinated PNS axons, while panel (B) shows a bundle of myelinated PNS axons from a CMT1A patient (in both cases the scale bar is 20 μm). Panel (C) shows an underwound tomacula myelin segment, as is most common in HNPP (scale bar is 2.5 μm). (D) Myelin from a healthy mouse (a) and from a 5 month old HNPP (PMP22^+,0^) mouse (b,c). The small arrows point out nodes of Ranvier, whereas the large arrows point out both internodal and paranodal tomacula. Panels A and B represent unpublished data provided by the courtesy of Professor Steve S. Scherer of the University of Pennsylvania. Panels C and D are reproduced with permission from panels 2B and 6a-c of Adlkofer et al. (1997).^315^ Copyright 1997 Society for Neuroscience.

In the following sections, we overview what is known about how the genetic defects associated with the *PMP22* gene cause these distinct types of CMT.

### 15.1 How Do Variations in the Sequence of PMP22 Cause Type 1E CMT?

CMT1E is a demyelinating form of CMT caused by dominant *PMP22* variants that most often encode PMP22 with a single-amino-acid change. Depending on which variant is causative, the severity of CMT1E can range from mild to severe, with the most severe and early onset cases being classified as DSS. In the older literature, the mild and moderate forms of CMT1E were often classified as being subtypes of HNPP and CMT1A, respectively, though it is now appreciated that at least the more serious forms of CMT1E present differently than HNPP and CMT1A.^211^ Sites of known CMT1E variants are highlighted in the PMP22 topology diagram of Figure 14.

As earlier noted, many CMT1E variant forms of the PMP22 protein are prone to mistrafficking—failing to reach the plasma membrane or myelin, leading both to degradation and formation of intracellular inclusions, the locations of which may differ from variant to variant. In early work, it was shown that the surface trafficking efficiency (relative to intracellular entrapment) of CMT1E variants is usually much lower in HeLa AND HEK cells than the already marginal trafficking efficiency of WT PMP22.^261^ While disputed,^308^ it has also been proposed that heterozygously expressed CMT1E PMP22 variants can form heterodimers with WT PMP22,^155,300,301^ further reducing the already-modest surface trafficking efficiency of WT PMP22. Some of the native functions of PMP22 require surface trafficking of the protein, indicating that reduced trafficking efficiency of WT and variant forms of PMP22 is expected to result in, at least, partial loss of PMP22 function. However, given that HNPP (caused by loss of one of the two PMP22 alleles) is usually a mild disorder, it is generally believed that the more severe forms of CMT1E are driven largely by the adverse effects of the intracellular inclusions formed by mistrafficked PMP22.

The impact of accumulated mistrafficked PMP22 on SCs may differ from variant to variant, but should not be assumed to be “toxicity” defined as a tendency to kill the host SC. Rather CMT1E inclusions formed by the various CMT1E variants appear to disrupt SC differentiation and/or myelination without killing the cells.

Why are CMT1E variants usually even more prone to mis-traffic than WT PMP22? In the early 2000s, the Sanders lab set out to test the hypothesis that the underlying cause of mis-trafficking for most CMT1E PMP22 variants is thermodynamic destabilization of the correctly folded form of the protein. The thermodynamic stability of a protein reflects the position of the equilibrium between the folded and unfolded states. This hypothesis was inspired by studies of the folding of purified *E. coli* diacylglycerol kinase (DAGK), then studied as a model system for membrane protein misfolding (review in ref 255). Many mutant forms of DAGK are prone to form kinetically trapped structures that are aberrant in terms of both conformations and oligomeric state.^316^ Studies of DAGK revealed that the propensity of mutants to misfold correlates with the degree of destabilization of the native structure induced by each mutation.^317,318^ It was found that increased unfolding of DAGK results in increased misfolding: an unfolded membrane protein is a vulnerable membrane protein.

The conclusions made for DAGK inspired over a decade of study of PMP22 to determine if the propensity of CMT1E variants to mistraffic correlates with the degree to which these variants are destabilized. In early work, spectroscopy confirmed that the L16P (TrJ) and G150D (Tr) variant forms of PMP22 are less stable in detergent micelles than WT PMP22.^151^ Indeed, a comparative NMR study of WT and L16P PMP22 in micelles revealed that while WT PMP22 formed the expected bundle of 4 transmembrane helices, L16P adopted a structural state in which the first transmembrane helix was completely dissociated from the other three helices, the latter of which remained in contact with each other but only in a molten-globular state, bereft of stable tertiary structure (Figure 21).^299^

**Figure 21:**
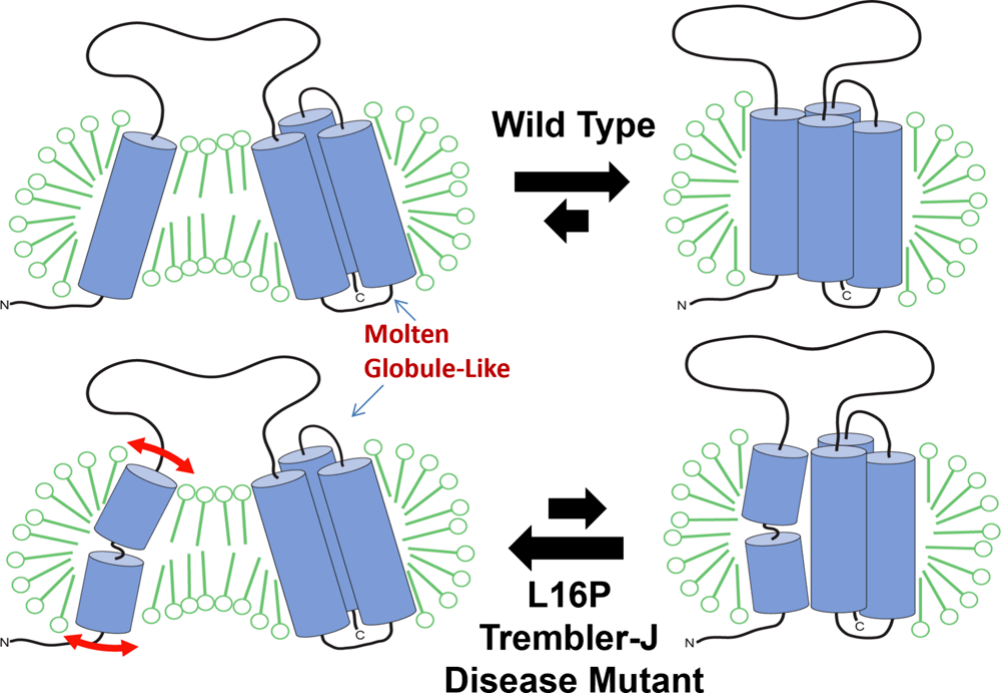
Folding equilibrium for WT (stable) PMP22 and for the L16P (unstable) variant. This was determined using NMR and other methods under conditions where PMP22 was solubilized in tetradecylphosphocholine micelles at 25 °C. The L16P (Trembler-J) disease mutation site is located in the first TM helix with the proline substitution resulting in the flexible hinge illustrated in the lower left panel. The “partially unfolded state” depicted on the left may be similar to the true physiological unfolded state. Further unfolding is restrained by the short loops connect TM2 to TM3 and TM3 to TM4. Caption (adapted) and figure reprinted with permission from Sakakura et al., 2011.^299^ Copyright 2011 Cell Press.

Early efforts to quantitate the thermodynamic stability of WT, L16P, G150D, and other CMT1E PMP22 variants were long thwarted by hysteresis seen between near-UV circular dichroism-monitored folded→denatured and denatured→folded curves,^151^ indicating that folding equilibrium conditions did not pertain when PMP22 in micelles was titrated with a denaturing detergent or vice versa. This problem was eventually solved by recognition that both denaturant-induced unfolding and refolding of micellar PMP22 are very slow processes, requiring on the order of hours to reach completion.^170^ This realization enabled the classical denaturant titration/removal unfolding/folding experiment to be conducted for WT PMP22 in detergent micelles under true equilibrium conditions, this time with the unfolding and refolding curves being seen to be identical. This enabled determination of the thermodynamic stability of WT PMP22.^170^ This method was then successfully applied to a series of CMT1E variants known to be associated with widely varying disease severity, ranging from mild to severe.^150^

In parallel with determining the thermodynamic stability of CMT1E variants, a quantitative trafficking assay was developed for PMP22 based on using fluorescently tagged antibodies and flow cytometry to quantitate, in single cell readout mode for thousands of cells, the surface and cell-interior trapped populations of PMP22 following expression in any one of several different model mammalian cell lines.^150^ From these two measurements, it is possible to also determine the total level of PMP22 (surface + internal) and the surface trafficking efficiency (surface/total × 100). This method was applied to both WT and the same set of CMT1E variants for which stability measurements were made.^150^ As a quantitative clinical indicator of the severity of CMT, values of the measured nerve conduction velocities (NCVs) were collected from the medical literature, describing CMT1E patients who were heterozygous for the variants examined in this study.

Figure 22 shows the correlations made using the thermodynamic stability, cell trafficking, and clinical NCV data collected as summarized above.^150^ As can be seen, the correlation between PMP22 stability and trafficking efficiency is clear, consistent with the driving hypothesis that it is thermodynamic instability that drives the protein misfolding events that lead to mis-trafficking and/or degradation of CMT1E PMP22 variants. Moreover, it is seen that patient NCVs correlate well with both PMP22 stability and trafficking efficiency. These results suggest that, at least for the variants represented in these plots, amino acid variation-induced instability is the fundamental molecular defect that is responsible for most cases of CMT1E. Earlier work and an important recent clinical study for a much larger set of CMT1E variants support this relationship between reduced surface trafficking and disease severity.^261,319,320^ The recent study also confirmed that the locations of the amino acid variations that cause the more severe disease phenotypes tend to be located in the transmembrane domain, while variant sites associated with less severe phenotypes tend to be located in the extracellular domain. This important observation is consistent with the notion that the overall folding of PMP22 is driven largely by tertiary structural interactions occurring within the bundle of 4 α-helices that comprise the transmembrane domain (Figure 22B).

**Figure 22:**
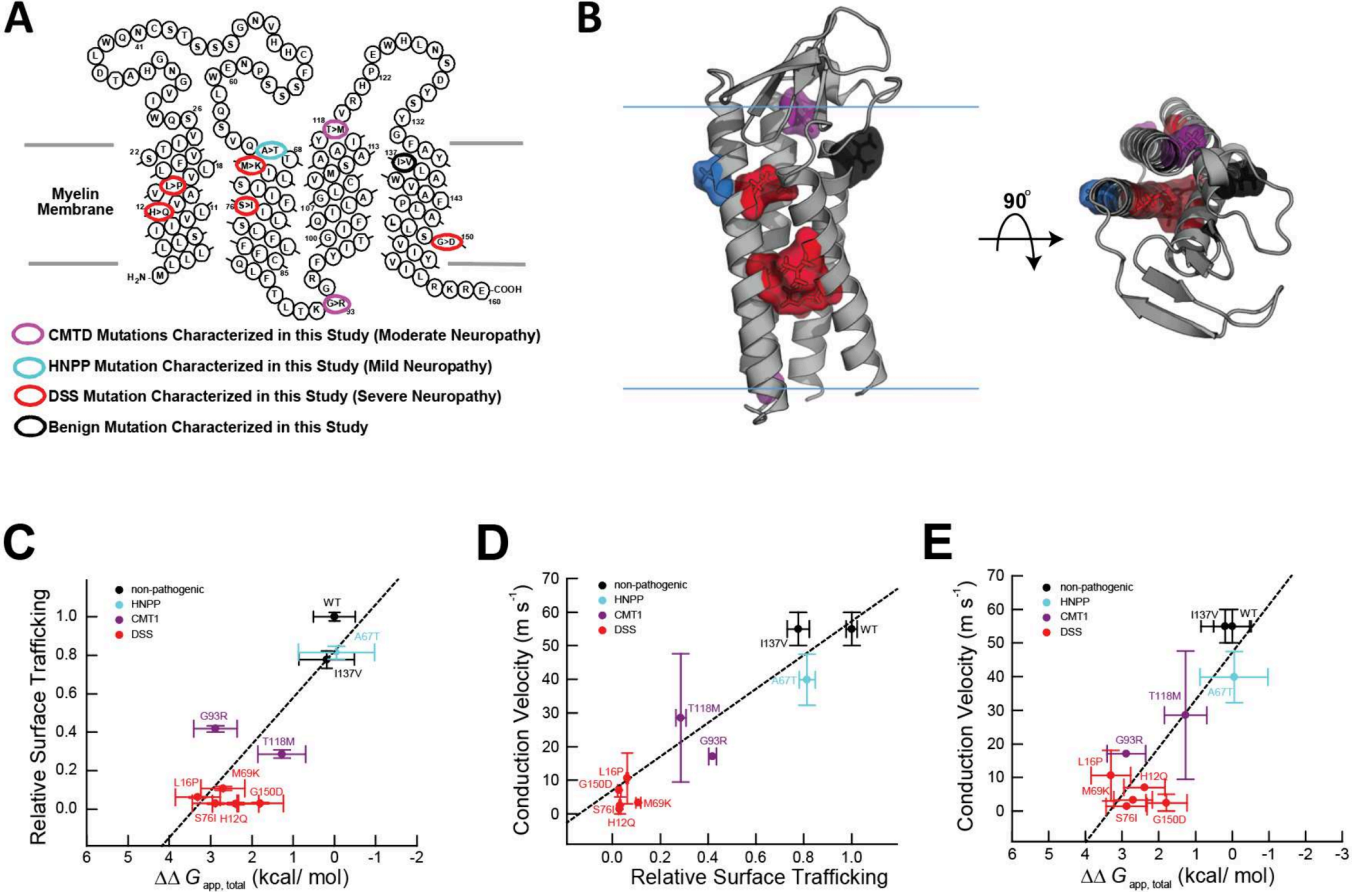
Thermodynamic destabilization of human PMP22 by disease variant forms results in mistrafficking of the protein and DSS (severe CMT1E), moderate (CMT1), or mild (HNPP-like) CMT1E. Panels (A) and (B) show the locations of the disease variants in the sequence and modeled 3-D structure of the PMP22, respectively. Panel (C) shows a strong correlation between surface trafficking efficiency and stability across the panel of tested PMP22 mutants. Panel (D) shows that the extent of surface trafficking correlates well with nerve conduction velocity in humans carrying each variant form. Healthy patients present with high conduction velocities, with reductions in conduction velocity correlating with disease severity. Panel (E) shows that there is also a strong correlation between the stability of PMP22 and nerve conduction velocity. Figure for panel (A) is adapted from Marinko et al. *Chemical Reviews* *119*, 5537–5606 (2019).^255^ Copyright 2019 American Chemical Society. Panels B-E were adapted from Figures 1 and 5 of Schlebach et al., *Journal of the American Chemical Society* (2015) *137*, 8758–8768.^150^ Copyright 2015 American Chemical Society.

An interesting exception to the observation that CMT1E variants are usually destabilized is the A67T variant, which involves a site located on the surface of the transmembrane domain. This variant is known to cause mild CMT1E (classified as HNPP in 2015) but exhibits stability and trafficking similar to WT (Figure 22). An important recent paper by Piper, Shy, and colleagues described a cis binding interface between PMP22 and P_0_, that includes A67 of PMP22 (see Figure 7).^78^ It was shown in that work that the A67T variation in PMP22 disrupts this PMP22/P_o_ complex, which is almost certainly the underlying defect associated with CMT1E caused by the A67T PMP22. This indicates that not all CMT1E variants cause disease by being destabilized, resulting in mistrafficking (see also ref 321). Interestingly, while P_0_ and PMP22 appear to form a complex and are synchronously expressed and codistributed under conditions of myelination,^261,322^ they do not appear to cotraffic as a complex.^78,261^

### 15.2 The Exceptional Case of Thr118Met PMP22 Provides Additional Insight into the Mechanisms of CMT1E

The T118M variant represented in the data of Figure 22 was regarded in previous work as an intermediate-severity CMT1E variant.^150^ However, as reflected by the high variability seen for patient NCVs associated with this variant and by conflicting literature reports as to whether heterozygous T118M PMP22 is actually CMT-causative, this variant is the subject of some controversy.^323–328^ It was recently observed, based on the gnomAD database^329^ (https://gnomad.broadinstitute.org/) for hundreds of thousands of sequenced human genomes, that the T118M variant of PMP22 is common in the human population, with a prevalence of roughly 1:100.^174^ This variation is therefore more common—by more than an order of magnitude—than all forms of CMT.^174,330,331^ This means that T118M, if it contributes to CMT, does so as a weak risk factor rather than as the determinative cause of the disease, with the former model having originally been proposed a number of years ago.^326^ For the rare heterozygous T118M PMP22 patients who do present with CMT1E, it is not yet clear what other risk factors are held by those patients that collude with the T118M variant to induce clinical presentation of CMT1E. It should be added that T118M/null and T118M/T118M patients have been identified and that their neuropathy is more severe than for either WT/null (HNPP) or WT/T118M patients.^324,332^ On the other hand, a WT/WT/T118M subject presented with classical CMT1A.^325^

T118M PMP22 represents a paradox. The experimental data of Figure 22 show that this variant form of PMP22 is significantly destabilized relative to WT PMP22 and that it traffics to the membrane surface with a much lower efficiency than WT.^150^ And yet, we now appreciate that it is only a weak risk factor for CMT. Why? A recent HeLa cell-based study of this protein indicates that while T118M is indeed unstable and mistraffics, T118M is resistant to the formation of intracellular inclusions and instead is more prone to be degraded.^174^ Indeed, T118M was seen not only to be less prone than the L16P and G150D PMP22 variants to form intracellular inclusions, but was also even less prone to do so than WT.^174^ This implies that the severe phenotypes of CMT1E seen for patients carrying variants such as L16P and G150D stem mainly from the pathogenic effects of the intracellular inclusions that they form rather than as a consequence of PMP22 loss-of-function effects. T118M seems to be only a weak risk factor for CMT1E because it is resistant to forming intracellular inclusions. Thr118 in PMP22 is located in the extracellular domain but close to the membrane.

### 15.3 How Does Underexpression of WT PMP22 Cause HNPP?

As noted earlier, HNPP is an inherited disorder in which the patient inherits only one WT allele, an example of haploinsufficiency as a disease mechanism.^34,124,125,333,334^ HNPP is generally thought of as a mild form of CMT, with typical symptoms being muscle weakness and atrophy, as well as focal numbness often associated with nerve compression. A distinctive feature of HNPP is that juxtanodal domains of some SC-generated myelin sheaths exhibit tomacula, which have a much higher caliber than normal myelin sheaths, but with myelin layers that are only loosely compacted (Figure 20C,D). Tomacula are associated with focal constrictions that pinch the axons, possibly impeding local propagation of the action potential^335^ or with failing to seal the paranodal domain, thus allowing disorganization of the nodal and juxtaparanodal regions.^203^ Moss and co-workers have provided evidence for a key role of PMP22 in organizing these domains of PNS.^202^ Tomacula may represent an extreme state of disorganization where the juxtanodal layering of the myelin membrane becomes “unglued”, suggesting a key role for PMP22 in normal intermembrane adhesion in myelin proximal to the nodes.

It has been reported that the level of PMP22 in the PNS myelin of HNPP patients is roughly 60% that of homozygous subjects.^336^ We suggest that the fact that this number is 60% rather than 50% reflects the marginally higher cell surface/myelin trafficking efficiency of PMP22 under HNPP conditions, likely because the folding efficiency of the WT protein is higher under the reduced total expression level conditions of HNPP,^250^ where there will be lower proteostasis stress.

Though the germline allele deletion event that causes HNPP in founder patients is as probable as the allele duplication that causes CMT1A, HNPP is reported to be much less common, implying that haploinsufficiency for *PMP22* is only a risk factor for HNPP. A majority of *PMP22* WT/null individuals are not diagnosed as suffering from HNPP.^38^ It is quite possible that many of those who are not diagnosed do experience symptoms such as pressure palsies (slow recovery from a compressed or pinched nerve) or other mild symptoms such as clumsiness, but these symptoms escape expert clinical notice. An additional contributor to the pathology of HNPP, as it is for other PMP22-linked forms of CMT, is aging. Certain symptoms of HNPP patients worsen with age.^337^ Beyond this, it is not known what other factors differentiate *PMP22* haploinsufficient individuals with clear HNPP symptoms from those who do not.

All indicators suggest that HNPP is caused by the partial loss of PMP22 function that accompanies reduced expression of WT PMP22. Peripheral axons of HNPP patients are more sensitive to mechanical compression than in healthy patients, likely reflecting the previously reviewed role of PMP22 in supporting the formation of the basal lamina of Schwann cells^243,244^ as well as for helping to support the SC cytoskeleton. While patient NCVs tend to be normal or nearly normal, other electrophysiological measurements can detect this condition.^338^ The fact that nerve conduction velocities of HNPP patients approach those of healthy individuals is consistent with the observation that PNS dysmyelination in HNPP is not as severe as that in most other forms of type 1 CMT. This highlights the likelihood that some functions of PMP22 in myelination are redundant, meaning that there are other proteins that can stand in, functionally, for PMP22 when its expression levels are reduced. For all organisms it is thought that a majority of proteins share important functions with at least one other protein, as is required for life to be robust.^339–341^

### 15.4 How Does Overexpression of WT PMP22 Cause Type 1A CMT?

Schwann cells, even those that have a third copy of the WT *PMP22* allele, express only modest amounts of PMP22 ^161,342^ and are generally normal^196^ until they encircle a large axon after radial sorting,^343^ at which point signals such as NRG-1 from the axon trigger formation of a SC basal lamina followed by myelination.^161^ The transcription of the *PMP22* gene in SCs is controlled by powerful SC-specific promoters and enhancers that are activated during myelination to drive high-level expression of the PMP22 protein.^11,123^ It has been shown that mRNA levels for PMP22 in myelinating CMT1A SCs are indeed higher than in healthy patients.^223,344,345^ Overexpression of PMP22 leads to upregulation of ERAD,^346^ saturation of the proteasome,^223,347,348^ activation of other proteostasis stress pathways,^254,303,347–352^ and formation of intracellular inclusions.^347,348^ It is the overexpression of PMP22 in myelinating SCs that triggers the development of CMT1A.^87^ This is perhaps most elegantly shown using an engineered mouse that overexpresses PMP22 but under the negative control of tetracycline.^353^ When tetracycline was introduced via ingestion, overexpression of PMP22 in this mouse was halted, and CMT1A-like dysmyelination was resolved. When tetracycline was removed to re-enable PMP22 overexpression, disease-like conditions returned. While activation of P0 and MBP expression occurs in concert with activation of PMP22 upon the initiation of myelination,^232^ overexpression of PMP22 in CMT1A does not appear to alter the levels of P_0_ or MBP in myelin.^342,354^ The levels of PMP22 in myelin under CMT1A conditions have been variously reported to be normal,^342,354^ increased,^87,123,336,355–357^ or variable.^355,358^ These contrasting results may reflect, to some degree, differences in the stages of the disease at which the measurements were made.

As noted, soon after birth radial sorting takes place and PNS axons are *de novo* myelinated. PMP22 is expressed upon activation of myelination.^87,89,257^ Overexpression of PMP22 seems to result in difficulty by at least some SCs to distinguish small caliber axons (normally not myelinated) from thicker axons. As a result, myelin does form, but the average axonal gauge is smaller than usual, with higher gauge axons being hypomyelinated, particularly at later times after *de novo* myelination, while small caliber axons are sometimes hypermyelinated.^33,101,217,359–362^ The formation of “not quite right” myelin either sets the stage or occurs in parallel—the literature is not clear—with defects in Schwann cell differentiation, the arrest of further myelination and altered SC growth and proliferation, with apoptosis also sometimes being induced (see refs 17, 22, 24, 196, 197, 211, 222, 224–226,^228^,^362^,^363^ ). These issues seem to result in dysmyelination and demyelination. Included in the defects is dedifferentiation of SCs, which seems to be closely associated with onion bulb formation, where SCs cluster near axons but fail to myelinate.^24,221,364–367^ What is not yet well-established is the degree to which these problems reflect PMP22-related events that occur at the very initial stage of myelination, which occurs mainly shortly after birth when the SC/myelin units first develop. This is as opposed to PMP22-related problems that arise during childhood and adolescence when the internodal lengths of the established myelin units normally increase in response to axonal lengthening with skeletal growth.

As adulthood is approached, this early stage of CMT1A seems to transition into a new and long-term steady state^33,361,368^ where large gauge axons are generally hypomyelinated, some axons are demyelinated, onion bulbs are abundant, motor nerve conduction velocity is subnormal, a variety of defects in the myelin structure are observed (see refs 101, 202, 342, 348, 354, 359, 360, 365,^369–371^ ), there are problems with the ion channels responsible for saltatory conduction,^372^ there is loss of innervation of muscle tissue and of neuromuscular junctions,^373^ and secondary axonal damage occurs leading to axon degeneration^117,236,237,370^ (see also Figure 20D). Once this chronic phase of the disease is established, CMT1A advances only slowly,^361^ with progression being most evident via reductions in the compound motor axon potential, which largely reflect secondary axonal degeneration.^368^ The state of PMP22 expression in this lifelong chronic phase of the disease has been controversial,^123^ with varying reports that expression levels are high^336,356^ or variable.^355,358,374^ One possibility is that, while the amount of PMP22 protein in myelin is high under conditions of active myelination, additional expression of the protein is low except at the latter stages of nerve injury repair during subsequent remyelination.^89,358,375^

While healthy SC-myelin sheaths are meant to last for the lifetime of a patient,^103,104^ the *t*_1/2_ value for PMP22 in compact myelin does not appear to have ever been measured. While the *t*_1/2_ of PMP22 in actively myelinating cells has been measured to be on the order of 30–60 min,^159,160,168,376^ it is possible that this mainly reflects the kinetics of PMP22 folding quality control leading to degradation during the intense burst of expression that occurs during myelination rather than the *t*_1/2_ for that fraction of PMP22 that folds correctly and traffics into myelin. It is known, for example, that P_0_ turns over very slowly.^99^ Nonetheless, there is at least some evidence that WT PMP22 continues to be turned over (and replenished) even after myelin formation.^161^

Based on our understanding of the literature as summarized above, CMT1A includes an early phase that centers around the *de novo* biogenesis of myelinated peripheral nerves starting shortly after birth when the number of Schwann cell/myelin units associated with each axon is fixed, followed by increased myelin production to increase the internode length in response to growth, which continues until adulthood, at which stage PNS axon growth is completed and myelin expansion is no longer needed. The profound problems with myelination that occur during the early phase of the disease result in a set of responses or adjustments (dedifferentiation of SCs, onion bulb formation, etc.) that lead to the chronic form of the disease. During the chronic phase of CMT1A, the slow progression is aging-associated and is significantly associated with the secondary axonal damage that gradually accrues when myelination becomes increasingly suboptimal because the cells responsible for repair and remyelination express three copies of *PMP22*, the same as for the SCs that led to CMT1A conditions to begin with.

What Schwann cell processes drive the transition of CMT1A from its early phase to chronic phase is not fully understood beyond important roles for SC dedifferentiation and onion bulb formation. There has long been a debate in the literature about possible roles for oxidative stress, inflammation, and macrophages in CMT1A, including a recent suggestion that ferroptosis is involved.^86,116,346,347,377–380^ These processes are certainly relevant under conditions of PNS nerve injury and repair.^47,48,108–110^ Perhaps some of these roles are indeed relevant to early stage CMT and are also active during the early-to-chronic disease transition, which may also involve contributions from SC dedifferentiation and nonideal myelin repair. PMP22 is known to be involved in nerve repair,^89,381^ with its mRNA initially being down-regulated and the PMP22 protein being degraded immediately after nerve injury. PMP22 expression is then upregulated 3 weeks later, upon commencement of remyelination.^89^ A possible confounding factor in studies of the disease etiology of CMT is the likelihood of temporal overlap between early stage CMT (associated with *de novo* myelination associated with new axons, followed by internodal lengthening in response to growth) and chronic CMT involving imperfect repair of injured PNS nerve segments, which can occur at any stage of life.

Overexpression of PMP22 occurs when Schwann cells have generated a basal lamina and are facing the daunting task of expanding their membrane surface by a factor of several thousand^41,56^ to generate myelin. It also likely occurs when, upon reactivation of myelination, the internodes are lengthened in response to skeletal/axonal growth during childhood and adolescence. This expansion of myelin must almost certainly involve not only production of a huge amount of lipid but also (even under normal conditions) myelin proteins, including P_0_ and PMP22. It would be surprising if the expression of 1.5X the normal amount of PMP22 during myelination does not represent a difficult-to-manage burden to Schwann cells. Moreover, even when expressed at low levels, WT PMP22 folds and traffics inefficiently.^161,250^ It is therefore not surprising that there are many studies that report the intracellular accumulation of WT PMP22 in SCs from CMT animal models,^197,249,291,308^ cultured SCs,^249^ and CMT1A patients.^223,308,354^ This accumulation of PMP22 likely reflects a failure to promptly degrade misfolded PMP22 because of the oversaturation of the degradation pathways.

To summarize, accumulated misfolded PMP22 in SCs during the formidable task of myelination likely places an additional heavy burden on SCs, which adjust proteostatically to manage excess accumulated PMP22. ERAD is activated,^346^ autophagy is activated,^292^ expression of certain chaperones is upregulated,^347^ endocytosis is possibly disrupted,^282,382^ the number of lysosomes is increased,^347,348,350^ certain components of the unfolded protein response (UPR) are activated,^383^ and overall gene expression is down-regulated.^384^ Very possibly compounding this proteostasis stress are “gain of function” problems caused by too much functional PMP22, although this remains uncertain. As a consequence of all these stresses and aberrations, Schwann cell biology is altered, resulting in an altered cell cycle,^211,228^ altered proliferation,^224,225^ altered migration and motility,^196^ and problems with SC development and differentiation.^24,196,197,224,263,365,385^ These fundamental cellular problems are accompanied by altered expression of the p75 neurotrophin receptor,^386^ altered ErbB receptor tyrosine kinase signaling,^72^ dysregulation of lysosomes,^204^ down-regulation of ciliary neurotrophic factor (CNTF),^384,387^ sphingolipid defects/deficiencies,^217–220^ problems with cholesterol and lipid homeostasis,^166,211,218–220,384,387,388^ and impaired axon-SC interactions. These issuesresult in serious axonal problems: increased neurofilaments, decreased microtubules, degeneration, and loss.^235^ It is a tribute to the robust and plastic nature of SCs that they just do not die in response to PMP22 overexpression during myelination. They valiantly adjust their biology and fix what they can during the transition from early stage CMT1A to the chronic stage, but it is faulty CMT1A cells that are responsible for repair. While Schwann cells in adults are quiescent,^103,104^ when they and/or their associated axons are damaged they dedifferentiate, clear the damaged tissue, and then regenerate myelin (Figure 8). However, even in healthy adults, repaired SC/axon units are not as healthy as in the original replaced tissue.^104,107^ The situation is presumably worse under CMT1A conditions, where the SCs are burdened by overexpression of PMP22 possibly during repair and certainly during remyelination. It is not surprising that the state of PNS axon myelination during this chronic stage of CMT1A is unhealthy.

## 16 Animal Models for CMT1A Research and Drug Discovery

Much of what is thought to be known about the pathobiology of CMT1A and other PMP22-related forms of CMT derives from research involving rodent models. An erudite recent perspective outlines the general limitations of disease models for CMT.^389^ As reviewed elsewhere,^3,7,385,390,391^ the most commonly used mouse models for CMT1A are the C3 ^364^ and C22 ^392,393^ mice. The C22 mice have 7 copies of the human PMP22 gene and develop a severe neuropathy, much worse than typical CMT1A. As such, they are not considered the best model for the disease. The C3 mouse model was originally reported to contain 3–4 copies of the human PMP22 gene;^364^ however, a reassessment of the C3 mice indicated that these mice actually have 5 copies of the gene.^394^ The C3 mice also have the two endogenous mouse PMP22 alleles. There is also a CMT rat that expresses three human alleles in addition to the two rat alleles.^395^ While more difficult and expensive to work with, the CMT rat seems to most faithfully replicate human CMT1A,^7^ while the C3 mouse is deemed a better model than C22.^364^ Unfortunately, a unique CMT1A mouse model in which PMP22 overexpression and resulting CMT1A-like disease could be reduced by administering tetracycline^353^ is now lost. All these CMT mouse models have limitations in terms of how well they recapitulate human CMT1A.^374,396^ A recent and welcome development is generation of a new and more fully “humanized” mouse model in which both endogenous mouse PMP22 genes have been knocked out and human PMP22 genes have been introduced to generate both heterohumanized C3 mice (5 copies of human PMP22) and homohumanized mice (6–8 copies).^397^ Also available is an HNPP model mouse^315^ (one copy of PMP22) and the extensively studied *Trembler* ^294^ and *Trembler-J* ^295^ mice, which heterozygously express the G150D and L16P PMP22 CMT1E variants, respectively.

## 17 Potential Therapeutic Strategies for PMP22-Linked Forms of CMT

There is reason to hope that CMT1A, CMT1E and HNPP can be treated or even cured.^3,23–25,347,348^ While currently very expensive and not yet widely available for any disease, gene therapy, whereby the fundamental genetic defects that cause disease are directly corrected by editing, deleting, or replacing the defective gene, offers hope for a true cure for patients with PMP22-linked forms of CMT. Progress is being made toward this end.^398–404^ For CMT gene therapy, it seems desirable to treat the patient as early in life as possible—even before birth—to maximize the fraction of patient cells, especially SCs or their progenitors, that are genetically corrected. Also and ideally, this would restore normality to PNS myelination prior to the onset of early stage or chronic disease and possibly irreversible damage. A variety of RNA (RNAi, microRNA, etc.) or antisense DNA-based strategies for modulating PMP22 transcription or translation are also being explored.^3,405–409^

There is interest in developing biologicals or small-molecule drugs to either reduce PMP22 levels (for CMT1A) or to increase PMP22 levels (for HNPP).Such drugs would preferably act by altering levels of WT PMP22 transcription or translation and might need to be taken throughout the full lifetime of the patient.^161^ The feasibility that such PMP22-level-tuning drugs would be beneficial even for adult patients with chronic CMT is suggested by mouse and organoid studies in which WT PMP22 is overexpressed, resulting in disease pathology that was seen to be reversible following the restoration of normal PMP22 expression levels.^348,353^ The remarkable regenerative capacity of Schwann cells^210^ may therefore favor the feasibility and effectiveness of drugs for treating CMT1A and HNPP at all stages of life. Once again, such drug therapies would ideally be started early in childhood to avoid possibly irreversible PNS pathologies such as loss of axons.^101^ Examples of efforts to discover molecules that alter PMP22 transcription or translation levels are described in the literature.^410–413^

In the case of CMT1E caused by unstable mutants that are prone to mistraffic, drug therapies based on modulating total PMP22 protein levels are less appealing. For these forms of CMT1E, a more promising strategy would be to develop folding-corrective molecules that act by enhancing the stability of the PMP22 variant, providing the basis for avoiding either degradation or formation of inclusions, resulting in enhanced productive forward trafficking to the plasma membrane and/or myelin (Figure 23). This is a strategy akin to that which was successfully used to develop folding/trafficking correctors for the ΔF508 variant form of the cystic fibrosis transmembrane regulator (CFTR). The transformative Trikafta drug cocktail developed by Vertex is composed of two folding corrector molecules plus a third molecule that restores native-like chloride channel function to CTFR.^414^ Structurally speaking, PMP22 is a much simpler membrane protein than the multidomain CFTR and is known to fold cooperatively,^170^ such that it hopefully will require only a single folding corrector to enhance the folding of a whole spectrum of unstable CMT1E mutant forms. It also can be hoped that PMP22 will not require a functional corrector once correctly folded. It should be added that PMP22 folding corrector drugs might also prove effective for treating HNPP. While they might not alter total PMP22 expression, by increasing the normally modest efficiency of WT PMP22 folding and surface trafficking, such compounds could conceivably restore PMP22 functionality to near normal levels. Early efforts to discover molecules that bind directly to PMP22 to stabilize the protein are underway.^167,415^

**Figure 23:**
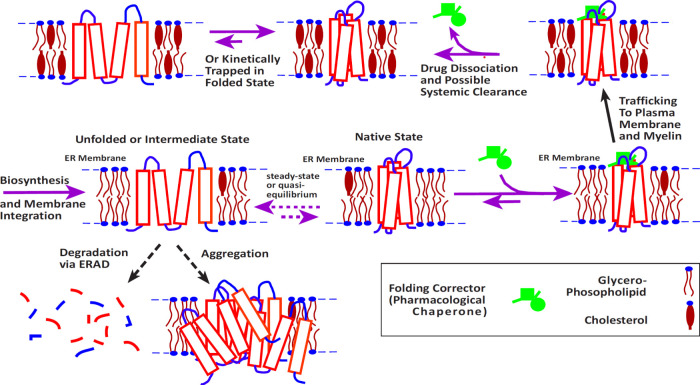
Model for how a small molecule folding corrector could both reduce degradation and puncta formation by PMP22 and promote forward trafficking of folded PMP22 to the cell surface and myelin membranes. The initial binding/rescue event occurs shortly after administration of the corrector, at which point the corrector concentration is fairly high. In this model once the PMP22 reaches the plasma membrane and the total corrector concentration is cleared (due to action of a cytochrome P_450_, for example) the folding corrector will dissociate and not be replenished (until another dose of the corrector is administered). We speculate that PMP22 could possibly remain mostly folded in the plasma and myelin membranes because it stabilized by the much higher levels of cholesterol and sphingolipids present there relatively to the ER. Adapted from Figure 26 of Marinko et al. *Chemical Reviews* (2019) *119*, 5537–5606.^255^ Copyright 2019 American Chemical Society.

For CMT1E, the number of patients is much smaller than for HNPP or CMT1A,^17,18,34,319^ such that there may be less economic incentive to invest in CMT1E drug discovery. However, some of the approaches that one can imagine pursuing to discover and develop CMT1A and/or HNPP drugs can readily be extended or adapted to find and develop molecules for CMT1E, which may lower the economic barriers to such efforts. It is also possible that mild forms of CMT1E and HNPP are underdiagnosed. If so, such underdiagnosis may be amended in the near future as whole genome sequence analysis becomes part of routine medical practice. It is probable that even CMT1E and HNPP patients with only very mild symptoms will be interested in safe and effective therapies if available.

Another set of therapeutic approaches is less direct and seeks to target or manage the cellular mayhem caused by PMP22 under CMT conditions. Such strategies include activation of chaperones, proteasomes, or autophagy pathways.^111,259,289,292,358,383,416–419^ Some investigators are targeting specific pathways of the unfolded protein response (UPR) that are triggered by overexpression of PMP22, resulting in such a strong UPR response that these normally helpful pathways become a contributor to CMT pathology and progression.^23,111,112,289,383,416,417,419–422^ Fasting has also been explored as a way to reduce PMP22 inclusions and improve disease symptoms in TrJ mice, where caloric reduction is thought to operate by promoting autophagy.^423,424^ Somewhat conversely, dietary supplementation of lipids has also been explored in animal CMT models as a potential therapy.^425,426^ Notterpek and co-workers have proposed that the ABCA1 cholesterol transporter may be a target for CMT1A.^427^ Finally, Prior et al. have presented data showing that inhibition of histone deacetylase 3 (HDAC3), an epigenetic regulator of myelination and nerve repair, may provide a therapeutic avenue.^394^

Summaries of previous and ongoing drug discovery efforts for both PMP22-linked forms of CMT and other types of CMT can be found in refs 3, 23–25, 28, 123, 210, 384, 398, 428–430 . We add that an uncommon aspect of both biologics and small-molecule drug discovery based on targeting biomolecules in Schwann cells is the need for the drugs to be able to pass the blood-nerve barrier (BNB) to reach their target in Schwann cells.^43,431,432^ Central structures of the BNB are the perineurium and epineurium (Figure 1). These barriers are similar to but not the same as those confronting CNS-targeted drugs that must cross the blood-brain barrier (BBB). In general, it is thought that the BNB is more permeable than the BBB.^432^ Interestingly, PMP22 is thought to be a component of both.^431^

## 18 Future Directions in PMP22-Related CMT Disease Research

### 18.1 There Is a Need to Conduct More Studies of PMP22 in SCs Under Myelinating Conditions

Much remains unknown about the functions of PMP22 or how it impacts CMT. Much also remains mysterious about the trafficking and intracellular locations of PMP22 under the CMT conditions. It is possible that a major hurdle to our understanding of PMP22 is the difficulty of conducting experiments on actively myelinating Schwann cells. Most previous cell biological studies have been carried out using immature nonmyelinating SCs or immortalized SCs^433–436^ in the absence of axons; however, it appears that the most important roles for PMP22 in both normal health and CMT occur after initial wrapping of an axon by a promyelinating Schwann cell and the subsequent transition to a myelinating Schwann cell. While *in vitro* myelination experiments are possible using dorsal root ganglia (DRG) neurons dissected from embryonic mice and Schwann cells^232,437–439^ (see also refs 436, 440 ), these are challenging experiments that take weeks to produce myelin. Moreover, the number of Schwann cells that myelinate is only a tiny fraction of the number of cells in the DRG cultures, such that they are not easily amenable to many of the vast array of tools available for classical cell biological experiments. In this regard, there may be hope for using human pluripotent stem cells and carefully crafted cell culture media as the basis for organoid formation^222,348,441–443^ or other approaches^444–446^ to generate conditions that better (or more conveniently) approximate actual myelination than is possible using model cell lines, even most Schwann cell lines.

### 18.2 There Is Impetus to Address the Incomplete Understanding of the Molecular Progress of CMT1A from Early Stage to Chronic Stage

In this review we have suggested that there is an early stage of CMT1A that is associated with the initial (*de novo*) myelination of new axons that have never previously been myelinated and then expansion of the SC/myelin units as their internodal lengths increase in response to growth. This early stage is followed by a transition to the chronic form of the disease which is characterized by the presence of abundant demyelinated or thinly myelinated axons and the presence of abundant onion bulbs. Very little is known about the molecular basis, including the role of PMP22, in the lengthening of myelin units during childhood and adolescent growth.^369,447^ We also suggest that the transition from the early stage disease to the chronic stage likely involves Schwann cell dedifferentiation and myelin repair, processes that continue occur throughout the chronic phase of the disease. However, this early to-chronic disease transition is not well-understood, including whether the transitional myelin repair processes are exactly the same as in the chronic form of the disease. Given that there is much thinking that PMP22-linked forms of CMT would best be treated very early in life, it may be especially important to better understand the differences between early stage CMT and chronic CMT, as well as the transition between them. Moreover, it is also very possible that “early-stage CMT” should be recognized as having two stages—the first being associated with *de novo* SC/axon pairing and initial myelination, and the second being associated with the additional myelination of existing units as required for internodal lengthening during childhood and adolescent growth. It is hoped that future studies will eventually illuminate these issues.

### 18.3 Is There a Gain-of-Function Component to CMT1A?

From single-cell trafficking studies of HEK cultures following transient transfection and expression of human PMP22, it appears that as total PMP22 expression levels increase, the amount of PMP22 that misfolds and is intracellularly retained increases more steeply than the surface-trafficked population. Nevertheless, this surface population continues to grow.^250^ Whether this trend occurs under actual physiological conditions of myelination has not been determined but seems likely. If so, this raises the question of whether the excess PMP22 that traffics to the cell surface and into myelin in CMT1A patients results in higher-than-normal levels of one or more of the functions of PMP22 in ways that are unhealthy, contributing to the etiology or progression of the disease. This remains an unresolved question, the answer to which may have implications for some prospective therapeutic modalities.

### 18.4 What Are the Functions and Interactions of PMP22?

As overviewed previously, PMP22 appears to be a multifunctional protein, but none of its putative functions are currently well-understood, even for those functions where there is already a sizable body of related literature, such as cholesterol homeostasis. In part, this reflects the difficulties summarized above in studying the functions of PMP22 under myelinating conditions. With respect to both the function and interactions of PMP22, there has been a burst of activity in the area of PMP22/CMT transcriptomics, metabolomics, and proteomics.^157,177,178,307,377,384^ However, these studies face some of the same limitations as functional and trafficking studies if they are not conducted under myelinating conditions. Notable efforts in this regard have appeared.^375,448,449^

### 18.5 What Are the Lifetimes of PMP22 and Other Key Myelin Proteins in Mature Myelin?

While myelinating Schwann cells are meant to last for a lifetime, barring injury or other damage, this does not mean that their proteins are unusually long-lived. There is evidence that some axonal proteins located at the nodes of Ranvier have lifetimes on the order of a month.^450^ Whether PMP22 in myelin has a very long lifetime and whether quiescent Schwann cells turn over and replenish degraded PMP22 is an important issue related to potential drugs that act by directly binding to PMP22 to correct its folding, to induce its degradation, or for other purposes. The required frequency of drug administration to lower PMP22 levels, for example, would depend on how long it takes for the PMP22 level to return to the concentration at which it needs to again be targeted. While there is a single paper^161^ indicating that PMP22 turns over fairly rapidly in myelinating Schwann cells, it is unclear whether the full population of PMP22 turns over or whether there are both labile and long-term-stable populations of PMP22 in SC/myelin units.

### 18.6 What Is the Nature of the Membrane in Compact Myelin—Raft-like, Fluid, or Mixed Phases?

Related to the issue of the PMP22 lifetime in myelin is the degree to which PMP22 is free to undergo lateral diffusion in myelin membranes. It is known that the 2-D translational diffusion of membrane proteins in the raft-like ordered phase is low, in contrast to the high mobility of membrane proteins in the disordered phase. Of course, even if PMP22 is in lipid rafts, the rafts themselves may be metastable or diffuse rapidly as a unit. Moreover, it is possible that PMP22 might occasionally spontaneously dissociate from one raft and diffuse rapidly through the disordered phase until it associates with another raft. However, if compact myelin is uniformly in the ordered phase, then PMP22 and P_0_ may be effectively fixed therein, suggesting that these proteins would have very long lifetimes, at least when located in compact myelin. For PMP22 and P_0_, there are reports that they do diffuse rapidly in myelinating SCs.^99,159^ However, whether this is for the full cellular population of these proteins or only for the fraction not present in compact myelin is unclear.

### 18.7 Besides Compact Myelin, Where Else in SC-Myelin-Axon Units Is PMP22 Located?

It is clear that PMP22 is present in the compact myelin. However, there are good reasons to believe that PMP22 should also be found in SLIs, where it likely is associated with junctions and/or actin/myosin. PMP22 also seems likely to be found in the abaxonal membrane, where it may associate with the basal lamina and/or the cytoskeleton. The localization of PMP22 within intact SM/myelin/axon units should be experimentally revisited.

### 18.8 What Are the Disease Modifying Factors for PMP22-Associated Forms of CMT Disease?

For patients with a particular gene variation that impacts *PMP22*, there can be a range of age-onset of symptoms and severity of symptoms. As noted, some CMT1A patients experience only mild neuropathy, whereas others experience much more serious symptoms and progression. Under heterozygous conditions, the T118M PMP22 variant seems to be only a weak risk factor, such that only a small fraction of all carriers will ever be diagnosed as having CMT. The disease-modifying factors, risk factors, and protective factors that modify disease severity are just beginning to be explored. It seems likely that these modifying factors will sometimes include gene variations in other known demyelinating type CMT disease genes, such as *MPZ*, *LITAF*, and *GJB1*. It can be hoped that the increasing collection and analysis of patient whole-genome sequences will provide the basis for breakthroughs (cf. refs 451,452 ). Of course, it is likely that there are nongenetic factors at play as well, such as diet, patient behavior, and environmental factors, which also need to be investigated.^33–36^

### 18.9 Is PMP22 Directly Druggable by Small Molecules?

Druggability would mean both that molecules can be found that bind tightly to PMP22 and also that, upon binding PMP22, they exert a desirable impact on its trafficking or functions. While proteomics studies of PMP22 are suggestive of the possibility that PMP22 undergoes direct binding with other proteins, the only well-validated examples are provided by calnexin, by anti-PMP22 antibodies used as experimental reagents for Western blots or other applications, and by the P0 protein, which binds PMP22 through lateral helix–helix interactions in the membrane plane.^78^ However, given that both the homology/Rossetta and alphaFold models of PMP22 are in agreement that the protein has clefts located on both the extracellular domain and near the intracellular membrane-water interface, this suggests the possibility that small molecules will be found that bind to these sites. Indeed, preliminary results from an NMR screen of a small molecule fragment library yielded at least one compound that binds to PMP22.^167^ Whether the affinity of such molecules can be optimized to reach drug-like levels (submicromolar K_D_) and whether binding to PMP22 will actually stabilize the protein and enhance trafficking remain to be seen.

We have reviewed much evidence that the proteostasis crisis in myelinating Schwan cells caused by WT PMP22 overexpression is, at the very least, one of the underlying drivers of CMT1A. This suggests that small-molecule PMP22 folding correctors might prove efficacious not only in treating CMT1E but also for CMT1A by reducing levels of misfolded and mistrafficked PMP22 in SCs. However, this would mean that a higher-than-normal level of correctly folded PMP22 would reach the plasma membrane and myelin. It is not yet clear what the pathophysiological consequences of too high a level of functional PMP22 would be. Further research is needed, and indeed, the availability of such folding corrective molecules for PMP22 might provide a powerful tool in probing this issue.

Finally, if avid small-molecule binders of PMP22 are discovered, they could be the building blocks for development either of proteolysis targeting chimeras (PROTACs)^453^ that direct PMP22 to the proteasome or for lysosome-targeting chimeras (LYTACs).^454^ Such compounds would promote PMP22 degradation to lower the total amount of PMP22, as would be desirable under the CMT1A conditions. Of course, whether this is possible depends on the validity of the assumption that the proteasomal and lysosomal degradation pathways are not already saturated under disease conditions. As reviewed herein, there is reason to suspect that this assumption may not be correct, especially for the proteasomal pathway. However, only additional data will resolve this question.

## 19 Conclusions

In the over 30 years since PMP22 was first recognized as being the cause of CMT1A, CMT1E, and HNPP, the literature describing the molecular and cellular biology of these disorders and our understanding of PMP22 have grown to impressive proportions. Moreover, public awareness is increasing of CMT and how common this disease is, thanks in part to the patient advocacy and other activities of organizations such as the Charcot-Marie-Tooth Association and the CMT Research Foundation, to name only two. Nevertheless, there is much that we still do not know about PMP22-linked forms of CMT, not to mention the numerous less common forms of this disease caused by defects in other genes. It can be expected that further advances will sharpen and focus efforts to develop therapeutics that address the molecular bases of the various forms of CMT. The time would appear to be ripe for greater engagement in CMT research by chemists, who can bring tools and approaches to the table that have hitherto been used only sparingly, if at all. This can be expected to speed the prosecution of both a more profound understanding of the molecular basis for the functions and dysfunctions of PMP22 and the rational pursuit of CMT therapeutics.

## References

[1] Berger P., Niemann A., Suter U. (2006). Schwann cells and the pathogenesis
of inherited motor and sensory neuropathies (Charcot-Marie-Tooth disease). Glia.

[2] Berger P., Young P., Suter U. (2002). Molecular
cell biology of Charcot-Marie-Tooth
disease. Neurogenetics.

[3] Boutary S., Echaniz-Laguna A., Adams D., Loisel-Duwattez J., Schumacher M., Massaad C., Massaad-Massade L. (2021). Treating PMP22
gene duplication-related Charcot-Marie-Tooth disease: the past, the
present and the future. Transl Res..

[4] Brennan K. M., Bai Y., Shy M. E. (2015). Demyelinating
CMT-what’s known, what’s
new and what’s in store?. Neurosci. Lett..

[5] Jetten A. M., Suter U. (2000). The peripheral myelin
protein 22 and epithelial membrane protein
family. Prog. Nucleic Acid Res. Mol. Biol..

[6] Klein C. J. (2020). Charcot-Marie-Tooth
Disease and Other Hereditary Neuropathies. Continuum
(Minneap Minn).

[7] Li J., Parker B., Martyn C., Natarajan C., Guo J. (2013). The PMP22 gene and its related diseases. Mol.
Neurobiol.

[8] Meyer
zu Horste G., Prukop T., Nave K. A., Sereda M. W. (2006). Myelin
disorders: Causes and perspectives of Charcot-Marie-Tooth neuropathy. J. Mol. Neurosci.

[9] Muller H. W. (2000). Tetraspan
myelin protein PMP22 and demyelinating peripheral neuropathies: new
facts and hypotheses. Glia.

[10] Naef R., Suter U. (1998). Many facets of the
peripheral myelin protein PMP22 in myelination
and disease. Microsc Res. Tech.

[11] Pantera H., Hu B., Moiseev D., Dunham C., Rashid J., Moran J. J., Krentz K., Rubinstein C. D., Won S., Li J., Svaren J. (2020). Pmp22 super-enhancer
deletion causes tomacula formation
and conduction block in peripheral nerves. Hum.
Mol. Genet..

[12] Pisciotta C., Shy M. E. (2018). Neuropathy. Handb. Clin. Neurol..

[13] Scherer S. S., Svaren J. (2024). Peripheral Nervous
System (PNS) Myelin Diseases. Cold Spring Harb
Perspect Biol..

[14] Suter U., Scherer S. S. (2003). Disease mechanisms in inherited neuropathies. Nat. Rev. Neurosci.

[15] Watila M. M., Balarabe S. A. (2015). Molecular and clinical features of inherited neuropathies
due to PMP22 duplication. J. Neurol Sci..

[16] Fridman V., Bundy B., Reilly M. M., Pareyson D., Bacon C., Burns J., Day J., Feely S., Finkel R. S., Grider T., Kirk C. A., Herrmann D. N., Laura M., Li J., Lloyd T., Sumner C. J., Muntoni F., Piscosquito G., Ramchandren S., Shy R., Siskind C. E., Yum S. W., Moroni I., Pagliano E., Zuchner S., Scherer S. S., Shy M. E., Inherited Neuropathies C. (2015). CMT subtypes and disease
burden in patients enrolled in the Inherited Neuropathies Consortium
natural history study: a cross-sectional analysis. J. Neurol Neurosurg Psychiatry.

[17] DiVincenzo C., Elzinga C. D., Medeiros A. C., Karbassi I., Jones J. R., Evans M. C., Braastad C. D., Bishop C. M., Jaremko M., Wang Z., Liaquat K., Hoffman C. A., York M. D., Batish S. D., Lupski J. R., Higgins J. J. (2014). The allelic spectrum
of Charcot-Marie-Tooth disease in over 17,000 individuals with neuropathy. Mol. Genet Genomic Med..

[18] Record C. J., Pipis M., Skorupinska M., Blake J., Poh R., Polke J. M., Eggleton K., Nanji T., Zuchner S., Cortese A., Houlden H., Rossor A. M., Laura M., Reilly M. M. (2024). Whole genome sequencing increases the diagnostic rate
in Charcot-Marie-Tooth disease. Brain.

[19] Abe K. T., Lino A. M., Hirata M. T., Pavanello R. C., Brotto M. W., Marchiori P. E., Zatz M. (2004). A novel stop codon
mutation in the PMP22 gene associated with a variable phenotype. Neuromuscul Disord.

[20] Pipis M., Won S., Poh R., Efthymiou S., Polke J. M., Skorupinska M., Blake J., Rossor A. M., Moran J. J., Munot P., Muntoni F., Laura M., Svaren J., Reilly M. M. (2023). Post-transcriptional
microRNA repression of PMP22 dose in severe Charcot-Marie-Tooth disease
type 1. Brain.

[21] Visigalli D., Castagnola P., Capodivento G., Geroldi A., Bellone E., Mancardi G., Pareyson D., Schenone A., Nobbio L. (2016). Alternative
Splicing in the Human PMP22 Gene: Implications in CMT1A Neuropathy. Hum Mutat.

[22] Johnson J. S., Roux K. J., Fletcher B. S., Fortun J., Notterpek L. (2005). Molecular
alterations resulting from frameshift mutations in peripheral myelin
protein 22: implications for neuropathy severity. J. Neurosci Res..

[23] Bolino A., D’Antonio M. (2023). Recent advances in the treatment of Charcot-Marie-Tooth
neuropathies. J. Peripher Nerv Syst.

[24] Hertzog N., Jacob C. (2023). Mechanisms and treatment
strategies of demyelinating and dysmyelinating
Charcot-Marie-Tooth disease. Neural Regen Res..

[25] Morena J., Gupta A., Hoyle J. C. (2019). Charcot-Marie-Tooth:
From Molecules
to Therapy. Int. J. Mol. Sci..

[26] Apgar T. L., Sanders C. R. (2022). Compendium of causative
genes and their encoded proteins
for common monogenic disorders. Protein Sci..

[27] Foley C., Schofield I., Eglon G., Bailey G., Chinnery P. F., Horvath R. (2012). Charcot-Marie-Tooth
disease in Northern England. J. Neurol Neurosurg
Psychiatry.

[28] Fridman V., Saporta M. A. (2021). Mechanisms and Treatments in Demyelinating CMT. Neurotherapeutics.

[29] Ma M., Li Y., Dai S., Chu M., Sun L., Liu L., Zhou J. C. (2023). A meta-analysis on the prevalence of Charcot-Marie-Tooth
disease and related inherited peripheral neuropathies. J. Neurol.

[30] Martyn C. N., Hughes R. A. (1997). Epidemiology of peripheral neuropathy. J. Neurol Neurosurg Psychiatry.

[31] Skre H. (1974). Genetic and
clinical aspects of Charcot-Marie-Tooth’s disease. Clin Genet.

[32] Burns J., Timmerman V., Laura M., Yiu E. M., D’Antonio M., Mukherjee-Clavin B., De Winter J., Scherer S. S. (2026). Charcot-Marie-Tooth
disease and related neuropathies. Nat. Rev.
Dis Primers.

[33] Thomas P. K., Marques W., Davis M. B., Sweeney M. G., King R. H., Bradley J. L., Muddle J. R., Tyson J., Malcolm S., Harding A. E. (1997). The phenotypic
manifestations of
chromosome 17p11.2 duplication. Brain.

[34] Cesaroni C. A., Caiazza L., Pisano G., Gnazzo M., Sigona G., Rizzi S., Pantani A., Frattini D., Fusco C. (2025). PMP22-Related
Neuropathies: A Systematic Review. Genes (Basel).

[35] Mathis S., Corcia P., Tazir M., Camu W., Magdelaine C., Latour P., Biberon J., Guennoc A. M., Richard L., Magy L., Funalot B., Vallat J. M. (2014). Peripheral myelin
protein 22 gene duplication with atypical presentations: a new example
of the wide spectrum of Charcot-Marie-Tooth 1A disease. Neuromuscul Disord.

[36] Bis-Brewer D. M., Fazal S., Zuchner S. (2020). Genetic modifiers and
non-Mendelian
aspects of CMT. Brain Res..

[37] De
Grado A., Serio M., Saveri P., Pisciotta C., Pareyson D. (2025). Charcot-Marie-Tooth disease: a review of clinical developments
and its management - What’s new in 2025?. Expert Rev. Neurother.

[38] Kumar N., Muley S., Pakiam A., Parry G. J. (2002). Phenotypic
Variability
Leads to Under-recognition of HNPP. J. Clin
Neuromuscul Dis.

[39] Vaeth S., Andersen H., Christensen R., Jensen U. B. (2021). A Search for Undiagnosed
Charcot-Marie-Tooth Disease Among Patients Registered with Unspecified
Polyneuropathy in the Danish National Patient Registry. Clin Epidemiol.

[40] Dubourg O., Mouton P., Brice A., LeGuern E., Bouche P. (2000). Guidelines
for diagnosis of hereditary neuropathy with liability to pressure
palsies. Neuromuscul Disord.

[41] Salzer J., Feltri M. L., Jacob C. (2024). Schwann Cell
Development and Myelination. Cold Spring Harb
Perspect Biol..

[42] Bolino A. (2021). Myelin Biology. Neurotherapeutics.

[43] Sun, Y. ; Zabihi, M. ; Li, Q. ; Li, X. ; Kim, B. J. ; Ubogu, E. E. ; Raja, S. N. ; Wesselmann, U. ; Zhao, C. Drug Permeability: From the Blood-Brain Barrier to the Peripheral Nerve Barriers, Adv. Ther (Weinh) 2023, 6. 10.1002/adtp.202200150. PMC10465108 37649593

[44] Zotter B., Dagan O., Brady J., Baloui H., Samanta J., Salzer J. L. (2022). Gli1 Regulates the Postnatal Acquisition of Peripheral
Nerve Architecture. J. Neurosci..

[45] Nave K. A., Werner H. B. (2014). Myelination of the
nervous system: mechanisms and functions. Annu.
Rev. Cell Dev Biol..

[46] Garbay B., Heape A. M., Sargueil F., Cassagne C. (2000). Myelin synthesis in
the peripheral nervous system. Prog. Neurobiol.

[47] Balakrishnan A., Belfiore L., Chu T. H., Fleming T., Midha R., Biernaskie J., Schuurmans C. (2021). Insights Into the Role and Potential
of Schwann Cells for Peripheral Nerve Repair From Studies of Development
and Injury. Front Mol. Neurosci.

[48] Fledrich, R. ; Kungl, T. ; Nave, K. A. ; Stassart, R. M. Axo-glial interdependence in peripheral nerve development. Development 2019, 146. 10.1242/dev.151704. 31719044

[49] Bunge R. P., Bunge M. B., Bates M. (1989). Movements of the Schwann cell nucleus
implicate progression of the inner (axon-related) Schwann cell process
during myelination. J. Cell Biol..

[50] Chernousov M. A., Yu W. M., Chen Z. L., Carey D. J., Strickland S. (2008). Regulation
of Schwann cell function by the extracellular matrix. Glia.

[51] Oliveira J.
T., Yanick C., Wein N., Gomez Limia C. E. (2023). Neuron-Schwann
cell interactions in peripheral nervous system homeostasis, disease,
and preclinical treatment. Front Cell Neurosci.

[52] Ozcelik M., Cotter L., Jacob C., Pereira J. A., Relvas J. B., Suter U., Tricaud N. (2010). Pals1 is a
major regulator of the
epithelial-like polarization and the extension of the myelin sheath
in peripheral nerves. J. Neurosci..

[53] Simons M., Snaidero N., Aggarwal S. (2012). Cell polarity
in myelinating glia:
from membrane flow to diffusion barriers. Biochim.
Biophys. Acta.

[54] D’Este E., Kamin D., Balzarotti F., Hell S. W. (2017). Ultrastructural
anatomy of nodes of Ranvier in the peripheral nervous system as revealed
by STED microscopy. Proc. Natl. Acad. Sci. U.
S. A..

[55] Scherer S. S. (1997). Molecular
genetics of demyelination: new wrinkles on an old membrane. Neuron.

[56] Webster H. D. (1971). The geometry
of peripheral myelin sheaths during their formation and growth in
rat sciatic nerves. J. Cell Biol..

[57] Perkins G. A., Ellisman M. H. (2011). Mitochondrial configurations
in peripheral nerve suggest
differential ATP production. J. Struct Biol..

[58] Follis R., Prabhu V. V., Carter B. D. (2025). The Influence
of Schwann Cell Metabolism
and Dysfunction on Axon Maintenance. Glia.

[59] Babetto E., Beirowski B. (2022). Of axons that
struggle to make ends meet: Linking axonal
bioenergetic failure to programmed axon degeneration. Biochim Biophys Acta Bioenerg.

[60] Terada N., Saitoh Y., Kamijo A., Yamauchi J., Ohno N., Sakamoto T. (2019). Structures and Molecular Composition
of Schmidt-Lanterman
Incisures. Adv. Exp. Med. Biol..

[61] Citi, S. ; Fromm, M. ; Furuse, M. ; Gonzalez-Mariscal, L. ; Nusrat, A. ; Tsukita, S. ; Turner, J. R. A short guide to the tight junction, J. Cell Sci. 2024, 137. 10.1242/jcs.261776. PMC11128289 38712627

[62] Spiegel I., Peles E. (2002). Cellular junctions
of myelinated nerves (Review). Mol. Membr. Biol..

[63] Scherer S. S., Arroyo E. J. (2002). Recent progress
on the molecular organization of myelinated
axons. J. Peripher Nerv Syst.

[64] Li J. (2008). Hypothesis
of double polarization. J. Neurol Sci..

[65] Suzuki H., Tani K., Tamura A., Tsukita S., Fujiyoshi Y. (2015). Model for
the architecture of claudin-based paracellular ion channels through
tight junctions. J. Mol. Biol..

[66] Samanta P., Wang Y., Fuladi S., Zou J., Li Y., Shen L., Weber C., Khalili-Araghi F. (2018). Molecular
determination of claudin-15 organization and channel selectivity. J. Gen Physiol.

[67] Krokengen O. C., Raasakka A., Klenow M. B., Pal A., Hetland O., Mularski A., Ruskamo S., Pedersen J. S., Simonsen A. C., Kursula P. (2025). On the synergy between myelin proteins P0, MBP, and
P2 in peripheral nerve major dense line formation. FEBS J..

[68] de
Waegh S. M., Lee V. M., Brady S. T. (1992). Local modulation
of neurofilament phosphorylation, axonal caliber, and slow axonal
transport by myelinating Schwann cells. Cell.

[69] Stassart R. M., Mobius W., Nave K. A., Edgar J. M. (2018). The Axon-Myelin
Unit in Development and Degenerative Disease. Front Neurosci.

[70] Birchmeier C., Bennett D. L. (2016). Neuregulin/ErbB
Signaling in Developmental Myelin Formation
and Nerve Repair. Curr. Top Dev Biol..

[71] Grigoryan T., Birchmeier W. (2015). Molecular
signaling mechanisms of axon-glia communication
in the peripheral nervous system. Bioessays.

[72] Lee S. M., Chin L. S., Li L. (2017). Dysregulation
of ErbB Receptor Trafficking
and Signaling in Demyelinating Charcot-Marie-Tooth Disease. Mol. Neurobiol.

[73] Willem M. (2016). Proteolytic
processing of Neuregulin-1. Brain Res. Bull..

[74] Zhang Y., Zhao Q., Chen Q., Xu L., Yi S. (2023). Transcriptional
Control of Peripheral Nerve Regeneration. Mol.
Neurobiol.

[75] Cristobal C. D., Lee H. K. (2022). Development of myelinating glia: An overview. Glia.

[76] Han S. H., Cho J. G., Park S. J., Shin Y. K., Hong Y. B., Han J. Y., Park H. T., Park J. I. (2025). Transcription Factors
and Coregulators in Schwann Cell Differentiation, Myelination, and
Remyelination: Implications for Peripheral Neuropathy. J. Neurosci Res..

[77] Inouye H., Kuo F. H., Denninger A. R., Weinhausen B., Burghammer M., Kirschner D. A. (2017). Myelin structure in unfixed, single
nerve fibers: Scanning X-ray microdiffraction with a beam size of
200nm. J. Struct Biol..

[78] Pashkova N., Peterson T. A., Ptak C. P., Winistorfer S. C., Guerrero-Given D., Kamasawa N., Ahern C. A., Shy M. E., Piper R. C. (2024). Disrupting the transmembrane domain
interface between
PMP22 and MPZ. causes peripheral neuropathy. iScience.

[79] Sakakura M., Tanabe M., Mori M., Takahashi H., Mio K. (2023). Structural bases for the Charcot-Marie-Tooth
disease induced by single
amino acid substitutions of myelin protein zero. Structure.

[80] Shapiro L., Doyle J. P., Hensley P., Colman D. R., Hendrickson W. A. (1996). Crystal
structure of the extracellular domain from P0, the major structural
protein of peripheral nerve myelin. Neuron.

[81] Raasakka A., Kursula P. (2020). How Does Protein Zero
Assemble Compact Myelin?. Cells.

[82] Shy M. E. (2006). Peripheral
neuropathies caused by mutations in the myelin protein zero. J. Neurol Sci..

[83] Kursula P. (2008). Structural
properties of proteins specific to the myelin sheath. Amino Acids.

[84] Martini R., Schachner M. (1997). Molecular bases of myelin formation
as revealed by
investigations on mice deficient in glial cell surface molecules. Glia.

[85] Krokengen O. C., Touma C., Mularski A., Sutinen A., Dunkel R., Ytterdal M., Raasakka A., Mertens H. D. T., Simonsen A. C., Kursula P. (2024). The cytoplasmic tail
of myelin protein zero induces
morphological changes in lipid membranes. Biochim
Biophys Acta Biomembr.

[86] Kobsar I., Hasenpusch-Theil K., Wessig C., Muller H. W., Martini R. (2005). Evidence for
macrophage-mediated myelin disruption in an animal model for Charcot-Marie-Tooth
neuropathy type 1A. J. Neurosci Res..

[87] Miyazaki T., Takeda Y., Murakami Y., Kawano H., Shimazu T., Toya S., Uyemura K. (1995). Distribution
of PASII/PMP22 and connexin
32 proteins in the peripheral nervous system. Neurochem. Int..

[88] Rosso G., Negreira C., Sotelo J. R., Kun A. (2012). Myelinating
and demyelinating
phenotype of Trembler-J mouse (a model of Charcot-Marie-Tooth human
disease) analyzed by atomic force microscopy and confocal microscopy. J. Mol. Recognit.

[89] Snipes G. J., Suter U., Welcher A. A., Shooter E. M. (1992). Characterization
of a novel peripheral nervous system myelin protein (PMP-22/SR13). J. Cell Biol..

[90] D’Urso D., Ehrhardt P., Muller H. W. (1999). Peripheral myelin protein 22 and
protein zero: a novel association in peripheral nervous system myelin. J. Neurosci..

[91] Hasse B., Bosse F., Hanenberg H., Muller H. W. (2004). Peripheral myelin
protein 22 kDa and protein zero: domain specific trans-interactions. Mol. Cell Neurosci.

[92] Uyemura K., Kitamura K. (1991). Comparative studies on myelin proteins
in mammalian
peripheral nerve. Comp Biochem Physiol C Comp
Pharmacol Toxicol.

[93] Sedzik J., Kotake Y., Uyemura K. (1998). Purification of PASII/PMP22-an
extremely
hydrophobic glycoprotein of PNS myelin membrane. Neuroreport.

[94] Sedzik J., Tsukihara T. (2000). Solubilization of PNS myelin membrane proteins by detergents. Neuroreport.

[95] Quarles R. H. (1997). Glycoproteins
of myelin sheaths. J. Mol. Neurosci.

[96] Svennerholm L., Bostrom K., Fredman P., Jungbjer B., Mansson J. E., Rynmark B. M. (1992). Membrane lipids
of human peripheral nerve and spinal
cord. Biochim. Biophys. Acta.

[97] Saher G., Quintes S., Mobius W., Wehr M. C., Kramer-Albers E. M., Brugger B., Nave K. A. (2009). Cholesterol
regulates the endoplasmic
reticulum exit of the major membrane protein P0 required for peripheral
myelin compaction. J. Neurosci..

[98] Saher G., Quintes S., Nave K. A. (2011). Cholesterol:
a novel regulatory role
in myelin formation. Neuroscientist.

[99] Gould R. M. (1977). Incorporation
of glycoproteins into peripheral nerve myelin. J. Cell Biol..

[100] Farrar M. A., Park S. B., Lin C. S., Kiernan M. C. (2013). Evolution
of peripheral nerve function in humans: novel insights from motor
nerve excitability. J. Physiol.

[101] Grandis M., Leandri M., Vigo T., Cilli M., Sereda M. W., Gherardi G., Benedetti L., Mancardi G., Abbruzzese M., Nave K. A., Nobbio L., Schenone A. (2004). Early abnormalities in sciatic nerve function and structure
in a rat model of Charcot-Marie-Tooth type 1A disease. Exp. Neurol..

[102] Kim J. K., Lee H. J., Park H. T. (2014). Two faces
of Schwann
cell dedifferentiation in peripheral neurodegenerative diseases: pro-demyelinating
and axon-preservative functions. Neural Regen
Res..

[103] Stierli S., Imperatore V., Lloyd A. C. (2019). Schwann cell plasticity-roles
in tissue homeostasis, regeneration, and disease. Glia.

[104] Stierli, S. ; Napoli, I. ; White, I. J. ; Cattin, A. L. ; Monteza Cabrejos, A. ; Garcia Calavia, N. ; Malong, L. ; Ribeiro, S. ; Nihouarn, J. ; Williams, R. ; Young, K. M. ; Richardson, W. D. ; Lloyd, A. C. The regulation of the homeostasis and regeneration of peripheral nerve is distinct from the CNS and independent of a stem cell population, Development 2018, 145. 10.1242/dev.170316. PMC6307893 30413560

[105] Friede R. L., Brzoska J., Hartmann U. (1985). Changes in
myelin sheath
thickness and internode geometry in the rabbit phrenic nerve during
growth. J. Anat.

[106] Simpson A. H., Gillingwater T. H., Anderson H., Cottrell D., Sherman D. L., Ribchester R. R., Brophy P. J. (2013). Effect of limb lengthening
on internodal length and conduction velocity of peripheral nerve. J. Neurosci..

[107] Villalon E., Barry D. M., Byers N., Frizzi K., Jones M. R., Landayan D. S., Dale J. M., Downer N. L., Calcutt N. A., Garcia M. L. (2018). Internode length
is reduced during
myelination and remyelination by neurofilament medium phosphorylation
in motor axons. Exp. Neurol..

[108] Park H. T., Kim J. K., Tricaud N. (2019). The conceptual
introduction
of the ″demyelinating Schwann cell″ in peripheral demyelinating
neuropathies. Glia.

[109] Gomez-Sanchez J. A., Carty L., Iruarrizaga-Lejarreta M., Palomo-Irigoyen M., Varela-Rey M., Griffith M., Hantke J., Macias-Camara N., Azkargorta M., Aurrekoetxea I., De Juan V. G., Jefferies H. B., Aspichueta P., Elortza F., Aransay A. M., Martinez-Chantar M. L., Baas F., Mato J. M., Mirsky R., Woodhoo A., Jessen K. R. (2015). Schwann cell autophagy, myelinophagy, initiates myelin
clearance from injured nerves. J. Cell Biol..

[110] Yuan Y., Wang Y., Wu S., Zhao M. Y. (2022). Review:
Myelin clearance is critical for regeneration after peripheral nerve
injury. Front Neurol.

[111] Rangaraju S., Verrier J. D., Madorsky I., Nicks J., Dunn W. A., Notterpek L. (2010). Rapamycin
activates autophagy and improves myelination in explant cultures from
neuropathic mice. J. Neurosci..

[112] Verdu E., Ceballos D., Vilches J. J., Navarro X. (2000). Influence
of aging on peripheral nerve function and regeneration. J. Peripher Nerv Syst.

[113] Lee S., Notterpek L. (2013). Dietary restriction
supports peripheral nerve health
by enhancing endogenous protein quality control mechanisms. Exp Gerontol.

[114] Graciani A. L., Gutierre M. U., Coppi A. A., Arida R. M., Gutierre R. C. (2023). Myelin, aging, and physical exercise. Neurobiol Aging.

[115] Mehdipour, M. ; Thakkar, V. ; Chang, S. Enhancing peripheral nerve regeneration in aging: the role of Schwann cells, c-Jun, and emerging therapeutic strategies. Geroscience 2025, 10.1007/s11357-025-01882-5. PMC13356191 40938501

[116] Park H. T., Kim Y. H., Lee K. E., Kim J. K. (2020). Behind
the pathology of macrophage-associated demyelination in inflammatory
neuropathies: demyelinating Schwann cells. Cell.
Mol. Life Sci..

[117] Krajewski K. M., Lewis R. A., Fuerst D. R., Turansky C., Hinderer S. R., Garbern J., Kamholz J., Shy M. E. (2000). Neurological
dysfunction and axonal degeneration in Charcot-Marie-Tooth disease
type 1A. Brain.

[118] Fridman V., Reilly M. M. (2015). Inherited Neuropathies. Semin Neurol.

[119] Rasmussen S. A., Pomputius A., Amberger J. S., Hamosh A. (2021). Viewing Victor
McKusick’s legacy through the lens of his bibliography. Am. J. Med. Genet A.

[120] Stenson P. D., Mort M., Ball E. V., Chapman M., Evans K., Azevedo L., Hayden M., Heywood S., Millar D. S., Phillips A. D., Cooper D. N. (2020). The Human Gene Mutation
Database (HGMD­((R))): optimizing its use in a clinical diagnostic
or research setting. Hum. Genet..

[121] Yoshimura A., Yuan J. H., Hashiguchi A., Ando M., Higuchi Y., Nakamura T., Okamoto Y., Nakagawa M., Takashima H. (2019). Genetic profile and onset features
of 1005 patients with Charcot-Marie-Tooth disease in Japan. J. Neurol Neurosurg Psychiatry.

[122] Marques W., Freitas M. R., Nascimento O. J., Oliveira A. B., Calia L., Melo A., Lucena R., Rocha V., Barreira A. A. (2005). 17p duplicated Charcot-Marie-Tooth
1A: characteristics of a new population. J.
Neurol.

[123] Pantera H., Shy M. E., Svaren J. (2020). Regulating PMP22 expression
as a dosage sensitive neuropathy gene. Brain
Res..

[124] Attarian S., Fatehi F., Rajabally Y. A., Pareyson D. (2020). Hereditary neuropathy with liability to pressure palsies. J. Neurol.

[125] Chen L., Zhang H., Li C., Yang N., Wang J., Liang J. (2025). Literature review of clinical analysis
of hereditary neuropathy with liability to pressure palsies. J. Neurol.

[126] Ahmat Amin M. K. B., Shimizu A., Ogita H. (2019). The Pivotal
Roles of
the Epithelial Membrane Protein Family in Cancer Invasiveness and
Metastasis. Cancers (Basel).

[127] Sanders C. R., Ismail-Beigi F., McEnery M. W. (2001). Mutations of peripheral
myelin protein 22 result in defective trafficking through mechanisms
which may be common to diseases involving tetraspan membrane proteins. Biochemistry.

[128] Nicolas W. J., Shiriaeva A., Martynowycz M. W., Grey A. C., Ruma Y. N., Donaldson P. J., Gonen T. (2025). Structure of the lens MP20 mediated
adhesive junction. Nat. Commun..

[129] Roberts O., Paraoan L. (2020). PERP-ing into diverse
mechanisms
of cancer pathogenesis: Regulation and role of the p53/p63 effector
PERP. Biochim Biophys Acta Rev. Cancer.

[130] Attardi L. D., Reczek E. E., Cosmas C., Demicco E. G., McCurrach M. E., Lowe S. W., Jacks T. (2000). PERP, an apoptosis-associated
target of p53, is a novel member of the PMP-22/gas3 family. Genes Dev..

[131] Suzuki H., Nishizawa T., Tani K., Yamazaki Y., Tamura A., Ishitani R., Dohmae N., Tsukita S., Nureki O., Fujiyoshi Y. (2014). Crystal structure of a claudin provides
insight into the architecture of tight junctions. Science.

[132] Nakamura S., Irie K., Tanaka H., Nishikawa K., Suzuki H., Saitoh Y., Tamura A., Tsukita S., Fujiyoshi Y. (2019). Morphologic determinant of tight junctions revealed
by claudin-3 structures. Nat. Commun..

[133] Twomey E. C., Yelshanskaya M. V., Grassucci R. A., Frank J., Sobolevsky A. I. (2016). Elucidation
of AMPA receptor-stargazin
complexes by cryo-electron microscopy. Science.

[134] Menuz K., Stroud R. M., Nicoll R. A., Hays F. A. (2007). TARP auxiliary
subunits switch AMPA receptor antagonists into partial agonists. Science.

[135] Kamalova A., Nakagawa T. (2021). AMPA receptor structure and auxiliary
subunits. J. Physiol.

[136] Hale W. D., Romero A. M., Koylass N., Warrick C. R., Qiu Z., Huganir R. L., Twomey E. C. (2025). Structure
of transmembrane AMPA receptor
regulatory protein subunit gamma2. Nat. Commun..

[137] Chen S., Zhao Y., Wang Y., Shekhar M., Tajkhorshid E., Gouaux E. (2017). Activation and Desensitization
Mechanism
of AMPA Receptor-TARP Complex by Cryo-EM. Cell.

[138] Herguedas, B. ; Watson, J. F. ; Ho, H. ; Cais, O. ; Garcia-Nafria, J. ; Greger, I. H. Architecture of the heteromeric GluA1/2 AMPA receptor in complex with the auxiliary subunit TARP gamma8. Science 2019, 364. 10.1126/science.aav9011. PMC6513756 30872532

[139] Zhao Y., Chen S., Yoshioka C., Baconguis I., Gouaux E. (2016). Architecture of fully occupied GluA2
AMPA receptor-TARP
complex elucidated by cryo-EM. Nature.

[140] Mittendorf K. F., Kroncke B. M., Meiler J., Sanders C. R. (2014). The homology
model of PMP22 suggests mutations resulting in peripheral neuropathy
disrupt transmembrane helix packing. Biochemistry.

[141] Jumper J., Evans R., Pritzel A., Green T., Figurnov M., Ronneberger O., Tunyasuvunakool K., Bates R., Zidek A., Potapenko A., Bridgland A., Meyer C., Kohl S. A. A., Ballard A. J., Cowie A., Romera-Paredes B., Nikolov S., Jain R., Adler J., Back T., Petersen S., Reiman D., Clancy E., Zielinski M., Steinegger M., Pacholska M., Berghammer T., Bodenstein S., Silver D., Vinyals O., Senior A. W., Kavukcuoglu K., Kohli P., Hassabis D. (2021). Highly accurate protein structure
prediction with AlphaFold. Nature.

[142] Klykov O., Gangwar S. P., Yelshanskaya M. V., Yen L., Sobolevsky A. I. (2021). Structure and desensitization of
AMPA receptor complexes with type II TARP gamma5 and GSG1L. Mol. Cell.

[143] Fleming J., Magana P., Nair S., Tsenkov M., Bertoni D., Pidruchna I., Lima Afonso M. Q., Midlik A., Paramval U., Zidek A., Laydon A., Kovalevskiy O., Pan J., Cheng J., Avsec Z., Bycroft C., Wong L. H., Last M., Mirdita M., Steinegger M., Kohli P., Varadi M., Velankar S. (2025). AlphaFold
Protein Structure Database and 3D-Beacons: New Data and Capabilities. J. Mol. Biol..

[144] Kister A., Kister I. (2023). Overview of myelin, major myelin
lipids, and myelin-associated proteins. Front
Chem..

[145] Snipes G. J., Suter U., Shooter E. M. (1993). Human peripheral
myelin protein-22 carries the L2/HNK-1 carbohydrate adhesion epitope. J. Neurochem.

[146] Morita I., Kizuka Y., Kakuda S., Oka S. (2007). Expression
and function of the HNK-1 carbohydrate. J. Biochem.

[147] Kitamura K., Uyemura K., Shibuya K., Sakamoto Y., Yoshimura K., Nomura M. (2000). Structure of a major
oligosaccharide
of PASII/PMP22 glycoprotein in bovine peripheral nerve myelin. J. Neurochem.

[148] Morise J., Takematsu H., Oka S. (2017). The role of human natural
killer-1 (HNK-1) carbohydrate in neuronal plasticity and disease. Biochim Biophys Acta Gen Subj.

[149] Grant O. C., Wentworth D., Holmes S. G., Kandel R., Sehnal D., Wang X., Xiao Y., Sheppard P., Grelsson T., Coulter A., Miller G., Singh A., Nagarajan M., Foley B. L., Woods R. J. (2026). Generating 3D models
of complex carbohydrates with GLYCAM-Web. Nat.
Methods.

[150] Schlebach J. P., Narayan M., Alford C., Mittendorf K. F., Carter B. D., Li J., Sanders C. R. (2015). Conformational Stability
and Pathogenic Misfolding of the Integral Membrane Protein PMP22. J. Am. Chem. Soc..

[151] Myers J. K., Mobley C. K., Sanders C. R. (2008). The peripheral neuropathy-linked
Trembler and Trembler-J mutant forms of peripheral myelin protein
22 are folding-destabilized. Biochemistry.

[152] Unal B., Tan H., Orbak Z., Kiki I., Bilici M., Bilici N., Aslan H., Kaplan S. (2005). Morphological
alterations produced by zinc deficiency in rat sciatic nerve: a histological,
electron microscopic, and stereological study. Brain Res..

[153] O’Dell B. L., Conley-Harrison J., Browning J. D., Besch-Williford C., Hempe J. M., Savage J. E. (1990). Zinc deficiency and peripheral neuropathy
in chicks. Proc. Soc. Exp Biol. Med..

[154] Liu N., Yamauchi J., Shooter E. M. (2004). Recessive,
but not dominant, mutations
in peripheral myelin protein 22 gene show unique patterns of aggregation
and intracellular trafficking. Neurobiol Dis.

[155] Tobler A. R., Notterpek L., Naef R., Taylor V., Suter U., Shooter E. M. (1999). Transport
of Trembler-J mutant peripheral
myelin protein 22 is blocked in the intermediate compartment and affects
the transport of the wild-type protein by direct interaction. J. Neurosci..

[156] Jung J., Coe H., Michalak M. (2011). Specialization
of endoplasmic
reticulum chaperones for the folding and function of myelin glycoproteins
P0 and PMP22. FASEB J..

[157] Holt I., Emery N., Gates M. A., Brown S. J., Shirran S. L., Fuller H. R. (2025). Characterising PMP22-Proximal
Partners
in a Schwann Cell Model of Charcot-Marie-Tooth Disease Type1A. Biology (Basel).

[158] Hanemann C. O., D’Urso D., Gabreels-Festen A. A., Muller H. W. (2000). Mutation-dependent
alteration in cellular distribution
of peripheral myelin protein 22 in nerve biopsies from Charcot-Marie-Tooth
type 1A. Brain.

[159] Dickson K. M., Bergeron J. J., Shames I., Colby J., Nguyen D. T., Chevet E., Thomas D. Y., Snipes G. J. (2002). Association
of calnexin with mutant peripheral myelin protein-22 ex vivo: a basis
for ″gain-of-function″ ER diseases. Proc. Natl. Acad. Sci. U. S. A..

[160] Colby J., Nicholson R., Dickson K. M., Orfali W., Naef R., Suter U., Snipes G. J. (2000). PMP22 carrying the
trembler or trembler-J mutation is intracellularly retained in myelinating
Schwann cells. Neurobiol Dis.

[161] Pareek S., Notterpek L., Snipes G. J., Naef R., Sossin W., Laliberte J., Iacampo S., Suter U., Shooter E. M., Murphy R. A. (1997). Neurons
promote the translocation
of peripheral myelin protein 22 into myelin. J. Neurosci..

[162] Zambon A. A., Pitt M., Laura M., Polke J. M., Reilly M. M., Muntoni F. (2020). A novel homozygous variant extending
the peripheral myelin protein 22 by 9 amino acids causes early-onset
Charcot-Marie-Tooth disease with predominant severe sensory ataxia. J. Peripher Nerv Syst.

[163] Ogbu C. P., de Las Alas M., Mandriota A. M., Liu X., Kapoor S., Choudhury J., Ruma Y. N., Goodman M. C., Sanders C. R., Gonen T., Kossiakoff A. A., Duffey M. E., Vecchio A. J. (2025). Biophysical
basis of tight junction
barrier modulation by a pan-claudin-binding molecule. PNAS Nexus.

[164] Zoltewicz S. J., Lee S., Chittoor V. G., Freeland S. M., Rangaraju S., Zacharias D. A., Notterpek L. (2012). The palmitoylation
state of PMP22 modulates epithelial cell morphology and migration. ASN Neuro.

[165] Fantini J., Epand R. M., Barrantes F. J. (2019). Cholesterol-Recognition
Motifs in Membrane Proteins. Adv. Exp. Med.
Biol..

[166] Zhou Y., Borchelt D., Bauson J. C., Fazio S., Miles J. R., Tavori H., Notterpek L. (2020). Subcellular
diversion of cholesterol by gain- and loss-of-function mutations in
PMP22. Glia.

[167] Li G. C., Castro M. A., Ukwaththage T., Sanders C. R. (2024). Optimizing NMR fragment-based drug screening for membrane
protein targets. J. Struct Biol. X.

[168] Ryan M. C., Notterpek L., Tobler A. R., Liu N., Shooter E. M. (2000). Role of the peripheral
myelin protein 22 N-linked glycan
in oligomer stability. J. Neurochem.

[169] Tobler A. R., Liu N., Mueller L., Shooter E. M. (2002). Differential
aggregation of the Trembler and Trembler J mutants of peripheral myelin
protein 22. Proc. Natl. Acad. Sci. U. S. A..

[170] Schlebach J. P., Peng D., Kroncke B. M., Mittendorf K. F., Narayan M., Carter B. D., Sanders C. R. (2013). Reversible
folding
of human peripheral myelin protein 22, a tetraspan membrane protein. Biochemistry.

[171] Mobley C. K., Myers J. K., Hadziselimovic A., Ellis C. D., Sanders C. R. (2007). Purification
and initiation of structural
characterization of human peripheral myelin protein 22, an integral
membrane protein linked to peripheral neuropathies. Biochemistry.

[172] Fantin, S. M. ; Parson, K. F. ; Yadav, P. ; Juliano, B. ; Li, G. C. ; Sanders, C. R. ; Ohi, M. D. ; Ruotolo, B. T. Ion mobility-mass spectrometry reveals the role of peripheral myelin protein dimers in peripheral neuropathy, Proc. Natl. Acad. Sci. U. S. A. 2021, 118. 10.1073/pnas.2015331118. PMC8092585 33893233

[173] Kitamura K., Suzuki M., Uyemura K. (1976). Purification and partial
characterization of two glycoproteins in bovine peripheral nerve myelin
membrane. Biochim. Biophys. Acta.

[174] Stefanski K. M., Li G. C., Marinko J. T., Carter B. D., Samuels D. C., Sanders C. R. (2023). How T118M peripheral
myelin protein
22 predisposes humans to Charcot-Marie-Tooth disease. J. Biol. Chem..

[175] Hutchison J. M., Lu Z., Li G. C., Travis B., Mittal R., Deatherage C. L., Sanders C. R. (2017). Dodecyl-beta-melibioside
Detergent Micelles as a Medium for Membrane Proteins. Biochemistry.

[176] Marinko J. T., Kenworthy A. K., Sanders C. R. (2020). Peripheral myelin
protein 22 preferentially partitions into ordered phase membrane domains. Proc. Natl. Acad. Sci. U. S. A..

[177] Marinko J. T., Wright M. T., Schlebach J. P., Clowes K. R., Heintzman D. R., Plate L., Sanders C. R. (2021). Glycosylation
limits forward trafficking of the tetraspan membrane protein PMP22. J. Biol. Chem..

[178] Stausberg D., M S., Arlt Fa, Fledrich R., Stassart R. M., Nave K. A., Urlaub H., Ewers D., Sereda M. W. (2025). High affinity cross-context
cellular assays reveal
novel protein-protein interactions of peripheral myelin protein of
22 kDa. bioRxiv 2025.09.03.673966.

[179] Xu W., Manichella D., Jiang H., Vallat J. M., Lilien J., Baron P., Scarlato G., Kamholz J., Shy M. E. (2000). Absence
of P0 leads to the dysregulation of myelin gene expression and myelin
morphogenesis. J. Neurosci Res..

[180] Neuberg D. H., Sancho S., Suter U. (1999). Altered molecular
architecture
of peripheral nerves in mice lacking the peripheral myelin protein
22 or connexin32. J. Neurosci Res..

[181] Carenini S., Neuberg D., Schachner M., Suter U., Martini R. (1999). Localization and functional roles
of PMP22 in peripheral nerves of P0-deficient mice. Glia.

[182] Sedzik J., Jastrzebski J. P., Grandis M. (2015). Glycans of myelin proteins. J.
Neurosci Res..

[183] Gallego R.
G., Blanco J. L., Thijssen-van Zuylen C. W., Gotfredsen C. H., Voshol H., Duus J. O., Schachner M., Vliegenthart J. F. (2001). Epitope diversity of N-glycans from bovine peripheral
myelin glycoprotein P0 revealed by mass spectrometry and nano probe
magic angle spinning 1H NMR spectroscopy. J.
Biol. Chem..

[184] Castillo G., Kleene R., Schachner M., Loers G., Torda A. E. (2021). Proteins
Binding to the Carbohydrate
HNK-1: Common Origins?. Int. J. Mol. Sci..

[185] Ptak C. P., Peterson T. A., Hopkins J. B., Ahern C. A., Shy M. E., Piper R. C. (2023). Homomeric interactions
of the MPZ.
Ig domain and their relation to Charcot-Marie-Tooth disease. Brain.

[186] Yoshimura T., Hayashi A., Handa-Narumi M., Yagi H., Ohno N., Koike T., Yamaguchi Y., Uchimura K., Kadomatsu K., Sedzik J., Kitamura K., Kato K., Trapp B. D., Baba H., Ikenaka K. (2017). GlcNAc6ST-1
regulates sulfation of N-glycans and myelination in the peripheral
nervous system. Sci. Rep.

[187] Needham L. K., Schnaar R. L. (1993). Carbohydrate recognition in the peripheral
nervous system: a calcium-dependent membrane binding site for HNK-1
reactive glycolipids potentially involved in Schwann cell adhesion. J. Cell Biol..

[188] Chou K. H., Ilyas A. A., Evans J. E., Quarles R. H., Jungalwala F. B. (1985). Structure of a glycolipid reacting with monoclonal
IgM in neuropathy and with HNK-1. Biochem. Biophys.
Res. Commun..

[189] Griffith L. S., Schmitz B., Schachner M. (1992). L2/HNK-1 carbohydrate
and protein-protein interactions mediate the homophilic binding of
the neural adhesion molecule P0. J. Neurosci
Res..

[190] Gao T., Lu N., Yao W., Wang X., Liu Y., Sun L., Cai M., Song Y., Rong R., Cao H. (2025). Lactone-Facilitated
Chemoenzymatic Synthesis of Sulfoglucuronosyl Paragloboside Oligosaccharides
Bearing the HNK-1 Epitope. Org. Lett..

[191] Bunyatov M. I., Boons G. J. (2025). Chemoenzymatic Synthesis
of Glycosphingolipids
Having an HNK-1 Epitope for Erythrocyte Cell Surface Remodeling. J. Am. Chem. Soc..

[192] Ieronymaki M., Nuti F., Brancaccio D., Rossi G., Real-Fernandez F., Cao Y., Monasson O., Larregola M., Peroni E., Uziel J., Sabatino G., Novellino E., Carotenuto A., Papini A. M., Rovero P. (2017). Structure-Activity
Relationship Studies, SPR Affinity Characterization, and Conformational
Analysis of Peptides That Mimic the HNK-1 Carbohydrate Epitope. ChemMedChem..

[193] Arulnangai R., Asia Thabassoom H., Vajiha Banu H., Thirugnanasambandham K., Ganesamoorthy R. (2025). Recent developments on ursolic acid
and its potential biological applications. Toxicol
Rep.

[194] Mittendorf K. F., Marinko J. T., Hampton C. M., Ke Z., Hadziselimovic A., Schlebach J. P., Law C. L., Li J., Wright E. R., Sanders C. R., Ohi M. D. (2017). Peripheral myelin
protein 22 alters membrane architecture. Sci.
Adv..

[195] Wilson C. H., Hartline D. K. (2011). Novel organization
and development
of copepod myelin. ii. nonglial origin. J. Comp
Neurol.

[196] Nobbio L., Vigo T., Abbruzzese M., Levi G., Brancolini C., Mantero S., Grandis M., Benedetti L., Mancardi G., Schenone A. (2004). Impairment of PMP22
transgenic Schwann cells differentiation in culture: implications
for Charcot-Marie-Tooth type 1A disease. Neurobiol
Dis.

[197] Niemann S., Sereda M. W., Suter U., Griffiths I. R., Nave K. A. (2000). Uncoupling of myelin assembly and schwann cell differentiation
by transgenic overexpression of peripheral myelin protein 22. J. Neurosci..

[198] Zhao J., Krystofiak E. S., Ballesteros A., Cui R., Van Itallie C. M., Anderson J. M., Fenollar-Ferrer C., Kachar B. (2018). Multiple claudin-claudin
cis interfaces are required
for tight junction strand formation and inherent flexibility. Commun. Biol..

[199] Hempel C., Protze J., Altun E., Riebe B., Piontek A., Fromm A., Lee I. M., Saleh T., Gunzel D., Krause G., Piontek J. (2020). Assembly of
Tight Junction
Strands: Claudin-10b and Claudin-3 Form Homo-Tetrameric Building Blocks
that Polymerise in a Channel-Independent Manner. J. Mol. Biol..

[200] Roux K. J., Amici S. A., Fletcher B. S., Notterpek L. (2005). Modulation
of epithelial morphology, monolayer permeability, and cell migration
by growth arrest specific 3/peripheral myelin protein 22. Mol. Biol. Cell.

[201] Notterpek L., Roux K. J., Amici S. A., Yazdanpour A., Rahner C., Fletcher B. S. (2001). Peripheral myelin protein 22 is a
constituent of intercellular junctions in epithelia. Proc. Natl. Acad. Sci. U. S. A..

[202] Moss K. R., Arowolo M. A., Gutierrez D. R., Hoke A. (2026). Aberrant Molecular Myelin Architecture in Charcot-Marie-Tooth Disease
Type 1A and Hereditary Neuropathy With Liability to Pressure Palsies. Glia.

[203] Guo J., Wang L., Zhang Y., Wu J., Arpag S., Hu B., Imhof B. A., Tian X., Carter B. D., Suter U., Li J. (2014). Abnormal junctions and permeability of myelin in PMP22-deficient
nerves. Ann. Neurol..

[204] Prior R., Silva A., Vangansewinkel T., Idkowiak J., Tharkeshwar A. K., Hellings T. P., Michailidou I., Vreijling J., Loos M., Koopmans B., Vlek N., Agaser C., Kuipers T. B., Michiels C., Rossaert E., Verschoren S., Vermeire W., de Laat V., Dehairs J., Eggermont K., van den Biggelaar D., Bademosi A. T., Meunier F. A., vandeVen M., Van Damme P., Mei H., Swinnen J. V., Lambrichts I., Baas F., Fluiter K., Wolfs E., Van Den Bosch L. (2024). PMP22 duplication dysregulates lipid homeostasis and
plasma membrane organization in developing human Schwann cells. Brain.

[205] Zhou Y., Miles J. R., Tavori H., Lin M., Khoshbouei H., Borchelt D. R., Bazick H., Landreth G. E., Lee S., Fazio S., Notterpek L. (2019). PMP22 Regulates Cholesterol Trafficking
and ABCA1-Mediated Cholesterol Efflux. J. Neurosci..

[206] Lee S., Amici S., Tavori H., Zeng W. M., Freeland S., Fazio S., Notterpek L. (2014). PMP22 is critical
for actin-mediated
cellular functions and for establishing lipid rafts. J. Neurosci..

[207] Hasse B., Bosse F., Muller H. W. (2002). Proteins
of peripheral
myelin are associated with glycosphingolipid/cholesterol-enriched
membranes. J. Neurosci Res..

[208] Erne B., Sansano S., Frank M., Schaeren-Wiemers N. (2002). Rafts in adult
peripheral nerve myelin contain major structural myelin proteins and
myelin and lymphocyte protein (MAL) and CD59 as specific markers. J. Neurochem.

[209] Stefanski, K. M. ; Huang, H. ; Luu, D. D. ; Li, G. C. ; Hutchison, J. M. ; Saksena, N. ; Fisch, A. J. ; Hasaka, T. P. ; Bauer, J. A. ; Kenworthy, A. K. ; Van Horn, W. D. ; Sanders, C. R. Pharmacological tools to modulate ordered membrane domains and order-dependent protein function. Commun. Chem. 2026, 10.1038/s42004-025-01874-8. PMC12881431 41507418

[210] Zhou Y., Notterpek L. (2016). Promoting
peripheral myelin repair. Exp. Neurol..

[211] Giambonini-Brugnoli G., Buchstaller J., Sommer L., Suter U., Mantei N. (2005). Distinct disease mechanisms
in peripheral neuropathies
due to altered peripheral myelin protein 22 gene dosage or a Pmp22
point mutation. Neurobiol Dis.

[212] Wilson H. L., Wilson S. A., Surprenant A., North R. A. (2002). Epithelial membrane proteins induce membrane blebbing
and interact with the P2 × 7 receptor C terminus. J. Biol. Chem..

[213] Nobbio L., Sturla L., Fiorese F., Usai C., Basile G., Moreschi I., Benvenuto F., Zocchi E., De Flora A., Schenone A., Bruzzone S. (2009). P2 ×
7-mediated increased intracellular calcium causes functional derangement
in Schwann cells from rats with CMT1A neuropathy. J. Biol. Chem..

[214] Vanoye C. G., Sakakura M., Follis R. M., Trevisan A. J., Narayan M., Li J., Sanders C. R., Carter B. D. (2019). Peripheral
myelin protein 22 modulates store-operated calcium channel activity,
providing insights into Charcot-Marie-Tooth disease etiology. J. Biol. Chem..

[215] Zhang N., Zhu H. P., Huang W., Wen X., Xie X., Jiang X., Peng C., Han B., He G. (2022). Unraveling
the structures, functions and mechanisms of epithelial membrane protein
family in human cancers. Exp Hematol Oncol.

[216] Li L., Lei Y., Li Y., Xie Y., Hui P., Zang X., Wu W., Wu F., Fan J., Wang J., Chen J., Chen Z., Hou Y. (2025). EMP1 safeguards
hematopoietic stem cells by suppressing sphingolipid metabolism and
alleviating endoplasmic reticulum stress. Nat.
Commun..

[217] Capodivento G., Camera M., Liessi N., Trada A., Debellis D., Schenone A., Armirotti A., Visigalli D., Nobbio L. (2024). Monitoring Myelin Lipid Composition
and the Structure of Myelinated Fibers Reveals a Maturation Delay
in CMT1A. Int. J. Mol. Sci..

[218] Visigalli D., Capodivento G., Basit A., Fernandez R., Hamid Z., Pencova B., Gemelli C., Marubbi D., Pastorino C., Luoma A. M., Riekel C., Kirschner D. A., Schenone A., Fernandez J. A., Armirotti A., Nobbio L. (2020). Exploiting Sphingo- and Glycerophospholipid Impairment
to Select Effective Drugs and Biomarkers for CMT1A. Front Neurol.

[219] Muller A., Grove K., Christen I., Kreider J., Santos C., Hoque S., Bidinosti M., Hatakeyama S., Zhang J. (2026). Metabolic signatures in sciatic nerve
of PMP22 transgenic rats provide insights into the pathogenesis of
charcot-marie-tooth disease type 1 A. Sci. Rep.

[220] Hellings T. P., Lamzira-Arichi N., Vreijling J. P., Mei H., Cats D., Kuipers T. B., Derks R. J. E., Heijink M., Blomberg N., Sidorov I., Giera M., Baas F., Fluiter K. (2026). Longitudinal analysis
of lipid changes in the sciatic
nerve caused by overexpression of PMP22 in murine models of CMT1A. J. Lipid Res..

[221] Vallat J. M. (2003). Dominantly
inherited peripheral neuropathies. J. Neuropathol
Exp Neurol.

[222] Shi L., Huang L., He R., Huang W., Wang H., Lai X., Zou Z., Sun J., Ke Q., Zheng M., Lu X., Pei Z., Su H., Xiang A. P., Li W., Yao X. (2018). Modeling the Pathogenesis of Charcot-Marie-Tooth Disease Type 1A
Using Patient-Specific iPSCs. Stem Cell Reports.

[223] Lee S., Bazick H., Chittoor-Vinod V., Al Salihi M. O., Xia G., Notterpek L. (2018). Elevated Peripheral
Myelin Protein 22, Reduced Mitotic
Potential, and Proteasome Impairment in Dermal Fibroblasts from Charcot-Marie-Tooth
Disease Type 1A Patients. Am. J. Pathol..

[224] Hanemann C. O., Rosenbaum C., Kupfer S., Wosch S., Stoegbauer F., Muller H. W. (1998). Improved culture methods to expand
Schwann cells with altered growth behaviour from CMT1A patients. Glia.

[225] Hanemann C. O., Muller H. W. (1998). Pathogenesis of
Charcot-Marie-Tooth
1A (CMT1A) neuropathy. Trends Neurosci.

[226] Fledrich R., Stassart R. M., Klink A., Rasch L. M., Prukop T., Haag L., Czesnik D., Kungl T., Abdelaal T. A., Keric N., Stadelmann C., Bruck W., Nave K. A., Sereda M. W. (2014). Soluble neuregulin-1
modulates disease pathogenesis in rodent models of Charcot-Marie-Tooth
disease 1A. Nat. Med..

[227] Fabbretti E., Edomi P., Brancolini C., Schneider C. (1995). Apoptotic phenotype induced by overexpression of wild-type
gas3/PMP22: its relation to the demyelinating peripheral neuropathy
CMT1A. Genes Dev..

[228] Atanasoski S., Scherer S. S., Nave K. A., Suter U. (2002). Proliferation
of Schwann cells and regulation of cyclin D1 expression in an animal
model of Charcot-Marie-Tooth disease type 1A. J. Neurosci Res..

[229] Takeda Y., Notsu T., Kitamura K., Uyemura K. (2001). Functional
analysis for peripheral myelin protein PASII/PMP22: is it a member
of claudin superfamily?. Neurochem. Res..

[230] Zoidl G., Blass-Kampmann S., D’Urso D., Schmalenbach C., Muller H. W. (1995). Retroviral-mediated
gene transfer
of the peripheral myelin protein PMP22 in Schwann cells: modulation
of cell growth. EMBO J..

[231] Brancolini C., Edomi P., Marzinotto S., Schneider C. (2000). Exposure at the cell surface is required for gas3/PMP22
To regulate both cell death and cell spreading: implication for the
Charcot-Marie-Tooth type 1A and Dejerine-Sottas diseases. Mol. Biol. Cell.

[232] D’Urso D., Schmalenbach C., Zoidl G., Prior R., Muller H. W. (1997). Studies on the effects of altered PMP22 expression
during myelination in vitro. J. Neurosci Res..

[233] Brancolini C., Marzinotto S., Edomi P., Agostoni E., Fiorentini C., Muller H. W., Schneider C. (1999). Rho-dependent
regulation of cell spreading by the tetraspan membrane protein Gas3/PMP22. Mol. Biol. Cell.

[234] Hu B., Arpag S., Zhang X., Mobius W., Werner H., Sosinsky G., Ellisman M., Zhang Y., Hamilton A., Chernoff J., Li J. (2016). Tuning PAK Activity to Rescue Abnormal
Myelin Permeability in HNPP. PLoS Genet.

[235] Sahenk Z., Chen L., Mendell J. R. (1999). Effects
of PMP22
duplication and deletions on the axonal cytoskeleton. Ann. Neurol..

[236] Moss K. R., Bopp T. S., Johnson A. E., Hoke A. (2021). New evidence
for secondary axonal degeneration in demyelinating neuropathies. Neurosci. Lett..

[237] Sahenk Z. (1999). Abnormal Schwann
cell-axon interactions in CMT neuropathies.
The effects of mutant Schwann cells on the axonal cytoskeleton and
regeneration-associated myelination. Ann. N.Y.
Acad. Sci..

[238] Court F. A., Wrabetz L., Feltri M. L. (2006). Basal lamina: Schwann
cells wrap to the rhythm of space-time. Curr.
Opin Neurobiol.

[239] Morales S. A., Telander D., Notterpek L., Wadehra M., Braun J., Gordon L. K. (2011). Rewiring integrin-mediated
signaling and cellular response with the peripheral myelin protein
22 and epithelial membrane protein 2 components of the tetraspan web. Invest Ophthalmol Vis Sci..

[240] Rao R. G., Sudhakar D., Hogue C. P., Amici S., Gordon L. K., Braun J., Notterpek L., Goodglick L., Wadehra M. (2011). Peripheral myelin protein-22 (PMP22)
modulates alpha 6 integrin expression in the human endometrium. Reprod Biol. Endocrinol.

[241] Nodari A., Previtali S. C., Dati G., Occhi S., Court F. A., Colombelli C., Zambroni D., Dina G., Del Carro U., Campbell K. P., Quattrini A., Wrabetz L., Feltri M. L. (2008). Alpha6beta4
integrin and dystroglycan
cooperate to stabilize the myelin sheath. J.
Neurosci..

[242] Amici S. A., Dunn W. A., Murphy A. J., Adams N. C., Gale N. W., Valenzuela D. M., Yancopoulos G. D., Notterpek L. (2006). Peripheral myelin protein 22 is in
complex with alpha6beta4 integrin, and its absence alters the Schwann
cell basal lamina. J. Neurosci..

[243] Rosso G., Liashkovich I., Gess B., Young P., Kun A., Shahin V. (2014). Unravelling
crucial biomechanical resilience of myelinated
peripheral nerve fibres provided by the Schwann cell basal lamina
and PMP22. Sci. Rep.

[244] Li J., Krajewski K., Lewis R. A., Shy M. E. (2004). Loss-of-function
phenotype of hereditary neuropathy with liability to pressure palsies. Muscle Nerve.

[245] Poitelon Y., Matafora V., Silvestri N., Zambroni D., McGarry C., Serghany N., Rush T., Vizzuso D., Court F. A., Bachi A., Wrabetz L., Feltri M. L. (2018). A dual role for
Integrin alpha6beta4 in modulating
hereditary neuropathy with liability to pressure palsies. J. Neurochem.

[246] Hall H., Liu L., Schachner M., Schmitz B. (1993). The L2/HNK-1 carbohydrate mediates
adhesion of neural
cells to laminin. Eur. J. Neurosci.

[247] Sanders C. R., Myers J. K. (2004). Disease-related
misassembly of membrane
proteins. Annu. Rev. Biophys. Biomol. Struct..

[248] Sanders C. R., Nagy J. K. (2000). Misfolding of membrane
proteins in
health and disease: the lady or the tiger?. Curr. Opin Struct Biol..

[249] Notterpek L., Ryan M. C., Tobler A. R., Shooter E. M. (1999). PMP22 accumulation
in aggresomes: implications for CMT1A pathology. Neurobiol Dis.

[250] Marinko J. T., Carter B. D., Sanders C. R. (2020). Direct relationship
between increased expression and mistrafficking of the Charcot-Marie-Tooth-associated
protein PMP22. J. Biol. Chem..

[251] van Meer G., Voelker D. R., Feigenson G. W. (2008). Membrane
lipids: where they are and how they behave. Nat. Rev. Mol. Cell Biol..

[252] Goblirsch B. R., Wiener M. C. (2020). Ste24: An Integral Membrane Protein
Zinc Metalloprotease with Provocative Structure and Emergent Biology. J. Mol. Biol..

[253] Fregno I., Molinari M. (2019). Proteasomal and lysosomal clearance
of faulty secretory proteins: ER-associated degradation (ERAD) and
ER-to-lysosome-associated degradation (ERLAD) pathways. Crit Rev. Biochem Mol. Biol..

[254] Volpi V. G., Touvier T., D’Antonio M. (2017). Endoplasmic
Reticulum Protein Quality Control Failure in Myelin Disorders. Front Mol. Neurosci.

[255] Marinko J. T., Huang H., Penn W. D., Capra J. A., Schlebach J. P., Sanders C. R. (2019). Folding and Misfolding of Human Membrane
Proteins in Health and Disease: From Single Molecules to Cellular
Proteostasis. Chem. Rev..

[256] Tannous A., Pisoni G. B., Hebert D. N., Molinari M. (2015). N-linked sugar-regulated
protein folding and quality control in the ER. Semin Cell Dev Biol..

[257] Notterpek L., Snipes G. J., Shooter E. M. (1999). Temporal expression
pattern of peripheral myelin protein 22 during in vivo and in vitro
myelination. Glia.

[258] Ryan M. C., Shooter E. M., Notterpek L. (2002). Aggresome
formation in neuropathy models based on peripheral myelin protein
22 mutations. Neurobiol Dis.

[259] Ha N., Choi Y. I., Jung N., Song J. Y., Bae D. K., Kim M. C., Lee Y. J., Song H., Kwak G., Jeong S., Park S., Nam S. H., Jung S. C., Choi B. O. (2020). A novel histone
deacetylase 6 inhibitor improves myelination
of Schwann cells in a model of Charcot-Marie-Tooth disease type 1A. Br. J. Pharmacol..

[260] Fernandez-Fuente G., Farrugia M. A., Peng Y., Schneider A., Svaren J., Puglielli L. (2024). Spatial selectivity of ATase inhibition
in mouse models of Charcot-Marie-Tooth disease. Brain Commun..

[261] Shames I., Fraser A., Colby J., Orfali W., Snipes G. J. (2003). Phenotypic differences between peripheral
myelin protein-22
(PMP22) and myelin protein zero (P0) mutations associated with Charcot-Marie-Tooth-related
diseases. J. Neuropathol Exp Neurol.

[262] Fontanini A., Chies R., Snapp E. L., Ferrarini M., Fabrizi G. M., Brancolini C. (2005). Glycan-independent
role of calnexin
in the intracellular retention of Charcot-Marie-tooth 1A Gas3/PMP22
mutants. J. Biol. Chem..

[263] Kraus A., Groenendyk J., Bedard K., Baldwin T. A., Krause K. H., Dubois-Dauphin M., Dyck J., Rosenbaum E. E., Korngut L., Colley N. J., Gosgnach S., Zochodne D., Todd K., Agellon L. B., Michalak M. (2010). Calnexin deficiency
leads to dysmyelination. J. Biol. Chem..

[264] Denzel A., Molinari M., Trigueros C., Martin J. E., Velmurgan S., Brown S., Stamp G., Owen M. J. (2002). Early postnatal death and motor disorders in mice congenitally
deficient in calnexin expression. Mol. Cell.
Biol..

[265] Meyer
zu Horste G., Hu W., Hartung H. P., Lehmann H. C., Kieseier B. C. (2008). The immunocompetence of Schwann cells. Muscle Nerve.

[266] Meyer
Zu Horste G., Heidenreich H., Lehmann H. C., Ferrone S., Hartung H. P., Wiendl H., Kieseier B. C. (2010). Expression of antigen
processing and presenting molecules by Schwann cells in inflammatory
neuropathies. Glia.

[267] Stoll G., Gabreels-Festen A. A., Jander S., Muller H. W., Hanemann C. O. (1998). Major histocompatibility
complex class II expression
and macrophage responses in genetically proven Charcot-Marie-Tooth
type 1 and hereditary neuropathy with liability to pressure palsies. Muscle Nerve.

[268] Lee H. K., Shin Y. K., Jung J., Seo S. Y., Baek S. Y., Park H. T. (2009). Proteasome inhibition
suppresses
Schwann cell dedifferentiation in vitro and in vivo. Glia.

[269] VerPlank J. J., Gawron J. M., Silvestri N. J., Wrabetz L., Feltri M. L. (2024). Knockout
of PA200 improves proteasomal
degradation and myelination in a proteotoxic neuropathy. Life Sci. Alliance.

[270] Ustrell V., Hoffman L., Pratt G., Rechsteiner M. (2002). PA200, a nuclear
proteasome activator involved in DNA repair. EMBO J..

[271] Mock J. Y., Xu Y., Ye Y., Clemons W. M. (2017). Structural basis
for regulation of the nucleo-cytoplasmic
distribution of Bag6 by TRC35. Proc. Natl. Acad.
Sci. U. S. A..

[272] Benarroch R., Austin J. M., Ahmed F., Isaacson R. L. (2019). The roles
of cytosolic quality control proteins, SGTA and the BAG6 complex,
in disease. Adv. Protein Chem. Struct Biol..

[273] Yu G., Klionsky D. J. (2022). Life and Death Decisions-The
Many Faces of Autophagy
in Cell Survival and Cell Death. Biomolecules.

[274] Belgrad J., De Pace R., Fields R. D. (2020). Autophagy
in Myelinating
Glia. J. Neurosci..

[275] Wu S. A., Li Z. J., Qi L. (2025). Endoplasmic
reticulum
(ER) protein degradation by ER-associated degradation and ER-phagy. Trends Cell Biol..

[276] Salomo-Coll C., Jimenez-Moreno N., Wilkinson S. (2025). Lysosomal
Degradation of ER Client Proteins by ER-phagy and Related Pathways. J. Mol. Biol..

[277] Reggiori F., Molinari M. (2022). ER-phagy: mechanisms, regulation,
and diseases connected to the lysosomal clearance of the endoplasmic
reticulum. Physiol Rev..

[278] Lim S. H. Y., Hansen M., Kumsta C. (2024). Molecular
Mechanisms
of Autophagy Decline during Aging. Cells.

[279] Zhong R., Richardson C. E. (2025). Maintenance
and Decline of Neuronal
Lysosomal Function in Aging. Cells.

[280] Nixon R. A. (2024). Autophagy-lysosomal-associated neuronal
death in neurodegenerative
disease. Acta Neuropathol.

[281] Kim S. H., Cho Y. S., Jung Y. K. (2025). Failure of lysosomal
acidification and endomembrane network in neurodegeneration. Exp Mol. Med..

[282] Chies R., Nobbio L., Edomi P., Schenone A., Schneider C., Brancolini C. (2003). Alterations in the Arf6-regulated
plasma membrane endosomal recycling pathway in cells overexpressing
the tetraspan protein Gas3/PMP22. J. Cell Sci..

[283] Wunderley, L. ; Zhang, L. ; Yarwood, R. ; Qin, W. ; Lowe, M. ; Woodman, P. Endosomal recycling tubule scission and integrin recycling involve the membrane curvature-supporting protein LITAF, J. Cell Sci. 2021, 134. 10.1242/jcs.258549. PMC8353527 34342350

[284] Lee S. M., Olzmann J. A., Chin L. S., Li L. (2011). Mutations
associated with Charcot-Marie-Tooth disease cause SIMPLE protein mislocalization
and degradation by the proteasome and aggresome-autophagy pathways. J. Cell Sci..

[285] Lacerda A. F., Hartjes E., Brunetti C. R. (2014). LITAF mutations
associated with Charcot-Marie-Tooth disease 1C show mislocalization
from the late endosome/lysosome to the mitochondria. PLoS One.

[286] Edgar J. R., Ho A. K., Laura M., Horvath R., Reilly M. M., Luzio J. P., Roberts R. C. (2020). A dysfunctional
endolysosomal pathway common to two sub-types of demyelinating Charcot-Marie-Tooth
disease. Acta Neuropathol Commun..

[287] Kutchukian C., Casas M., Dixon R. E., Dickson E. J. (2025). Disruption
of the PIKfyve complex unveils an adaptive mechanism to promote lysosomal
repair and mitochondrial homeostasis. Nat. Commun..

[288] Guo J., Ma Y. H., Yan Q., Wang L., Zeng Y. S., Wu J. L., Li J. (2012). Fig4 expression
in the rodent nervous
system and its potential role in preventing abnormal lysosomal accumulation. J. Neuropathol Exp Neurol.

[289] Rangaraju S., Madorsky I., Pileggi J. G., Kamal A., Notterpek L. (2008). Pharmacological induction of the heat shock response
improves myelination in a neuropathic model. Neurobiol Dis.

[290] Intisar A., Woo H., Kang H. G., Kim W. H., Shin H. Y., Kim M. Y., Kim Y. S., Mo Y. J., Lee Y. I., Kim M. S. (2023). Electroceutical approach ameliorates
intracellular PMP22 aggregation and promotes pro-myelinating pathways
in a CMT1A in vitro model. Biosens Bioelectron.

[291] Fortun J., Go J. C., Li J., Amici S. A., Dunn W. A., Notterpek L. (2006). Alterations
in degradative pathways and protein aggregation in a neuropathy model
based on PMP22 overexpression. Neurobiol Dis.

[292] Chittoor-Vinod, V. G. ; Lee, S. ; Judge, S. M. ; Notterpek, L. Inducible HSP70 is critical in preventing the aggregation and enhancing the processing of PMP22, ASN Neuro 2015, 7. 10.1177/1759091415569909. PMC4342366 25694550

[293] Vallat J. M., Sindou P., Garbay B., Preux P. M., Anani T., Richard L., Diot M. (1999). Expression of myelin
proteins in the adult heterozygous Trembler mouse. Acta Neuropathol.

[294] Suter U., Welcher A. A., Ozcelik T., Snipes G. J., Kosaras B., Francke U., Billings-Gagliardi S., Sidman R. L., Shooter E. M. (1992). Trembler mouse carries a point mutation
in a myelin gene. Nature.

[295] Suter U., Moskow J. J., Welcher A. A., Snipes G. J., Kosaras B., Sidman R. L., Buchberg A. M., Shooter E. M. (1992). A leucine-to-proline
mutation in the putative first transmembrane domain of the 22-kDa
peripheral myelin protein in the trembler-J mouse. Proc. Natl. Acad. Sci. U. S. A..

[296] Robertson A. M., King R. H., Muddle J. R., Thomas P. K. (1997). Abnormal
Schwann cell/axon interactions in the Trembler-J mouse. J. Anat.

[297] Martini R. (1997). Animal models for inherited peripheral
neuropathies. J. Anat.

[298] Kirkpatrick L. L., Brady S. T. (1994). Modulation of the
axonal microtubule
cytoskeleton by myelinating Schwann cells. J.
Neurosci..

[299] Sakakura M., Hadziselimovic A., Wang Z., Schey K. L., Sanders C. R. (2011). Structural
basis for the Trembler-J phenotype of Charcot-Marie-Tooth
disease. Structure.

[300] Naef R., Adlkofer K., Lescher B., Suter U. (1997). Aberrant protein
trafficking in Trembler suggests a disease mechanism for hereditary
human peripheral neuropathies. Mol. Cell Neurosci.

[301] Isaacs A. M., Jeans A., Oliver P. L., Vizor L., Brown S. D., Hunter A. J., Davies K. E. (2002). Identification
of
a new Pmp22 mouse mutant and trafficking analysis of a Pmp22 allelic
series suggesting that protein aggregates may be protective in Pmp22-associated
peripheral neuropathy. Mol. Cell Neurosci.

[302] Hara T., Hashimoto Y., Akuzawa T., Hirai R., Kobayashi H., Sato K. (2014). Rer1 and calnexin regulate endoplasmic
reticulum retention of a peripheral myelin protein 22 mutant that
causes type 1A Charcot-Marie-Tooth disease. Sci. Rep.

[303] Fortun J., Verrier J. D., Go J. C., Madorsky I., Dunn W. A., Notterpek L. (2007). The formation
of peripheral myelin
protein 22 aggregates is hindered by the enhancement of autophagy
and expression of cytoplasmic chaperones. Neurobiol
Dis.

[304] Fortun J., Li J., Go J., Fenstermaker A., Fletcher B. S., Notterpek L. (2005). Impaired proteasome
activity and
accumulation of ubiquitinated substrates in a hereditary neuropathy
model. J. Neurochem.

[305] Fortun J., Dunn W. A., Joy S., Li J., Notterpek L. (2003). Emerging role
for autophagy in the removal of aggresomes
in Schwann cells. J. Neurosci..

[306] Notterpek L., Shooter E. M., Snipes G. J. (1997). Upregulation
of
the endosomal-lysosomal pathway in the trembler-J neuropathy. J. Neurosci..

[307] Defilippi V., Petereit J., Handlos V. J. L., Notterpek L. (2024). Quantitative
proteomics unveils known and previously unrecognized alterations in
neuropathic nerves. J. Neurochem.

[308] D’Urso D., Prior R., Greiner-Petter R., Gabreels-Festen A. A., Muller H. W. (1998). Overloaded endoplasmic reticulum-Golgi
compartments, a possible pathomechanism of peripheral neuropathies
caused by mutations of the peripheral myelin protein PMP22. J. Neurosci..

[309] Saporta M. A., Katona I., Zhang X., Roper H. P., McClelland L., Macdonald F., Brueton L., Blake J., Suter U., Reilly M. M., Shy M. E., Li J. (2011). Neuropathy
in a human without the PMP22 gene. Arch Neurol.

[310] Sancho S., Magyar J. P., Aguzzi A., Suter U. (1999). Distal axonopathy
in peripheral nerves of PMP22-mutant mice. Brain.

[311] Lee J. H., Park S., Perez-Flores M. C., Chen Y., Kang M., Choi J., Levine L., Gratton M. A., Zhao J., Notterpek L., Yamoah E. N. (2024). Demyelination and Na­(+) Channel Redistribution
Underlie
Auditory and Vestibular Dysfunction in PMP22-Null Mice. eNeuro.

[312] Amici S. A., Dunn W. A., Notterpek L. (2007). Developmental
abnormalities in the nerves of peripheral myelin protein 22-deficient
mice. J. Neurosci Res..

[313] Adlkofer K., Martini R., Aguzzi A., Zielasek J., Toyka K. V., Suter U. (1995). Hypermyelination and
demyelinating
peripheral neuropathy in Pmp22-deficient mice. Nat. Genet..

[314] Gabreels-Festen A. (2002). Dejerine-Sottas
syndrome grown to maturity: overview
of genetic and morphological heterogeneity and follow-up of 25 patients. J. Anat.

[315] Adlkofer K., Frei R., Neuberg D. H., Zielasek J., Toyka K. V., Suter U. (1997). Heterozygous peripheral
myelin protein
22-deficient mice are affected by a progressive demyelinating tomaculous
neuropathy. J. Neurosci..

[316] Gorzelle B. M., Nagy J. K., Oxenoid K., Lonzer W. L., Cafiso D. S., Sanders C. R. (1999). Reconstitutive refolding
of diacylglycerol
kinase, an integral membrane protein. Biochemistry.

[317] Nagy J. K., Sanders C. R. (2004). Destabilizing mutations
promote membrane
protein misfolding. Biochemistry.

[318] Mi D., Kim H. J., Hadziselimovic A., Sanders C. R. (2006). Irreversible misfolding
of diacylglycerol kinase is independent of aggregation and occurs
prior to trimerization and membrane association. Biochemistry.

[319] Ward K. S., Ptak C. P., Pashkova N., Grider T., Peterson T. A., Pareyson D., Pisciotta C., Saveri P., Moroni I., Laura M., Burns J., Menezes M. P., Cornett K., Finkel R., Mukherjee-Clavin B., Sumner C. J., Greene M., Abdul Hamid O., Herrmann D., Sadjadi R., Walk D., Zuchner S., Reilly M. M., Scherer S. S., Inherited Neuropathy C., Piper R. C., Shy M. E. (2026). Charcot-Marie-Tooth
disease type 1E: clinical natural history and molecular impact of
PMP22 variants. Brain.

[320] Naef R., Suter U. (1999). Impaired intracellular trafficking
is a common disease mechanism of PMP22 point mutations in peripheral
neuropathies. Neurobiol Dis.

[321] Gess B., Jeibmann A., Schirmacher A., Kleffner I., Schilling M., Young P. (2011). Report of a novel mutation
in the PMP22 gene causing an axonal neuropathy. Muscle Nerve.

[322] Kuhn G., Lie A., Wilms S., Muller H. W. (1993). Coexpression
of PMP22 gene with MBP and P0 during de novo myelination and nerve
repair. Glia.

[323] Young P., Stogbauer F., Eller B., de Jonghe P., Lofgren A., Timmerman V., Rautenstrauss B., Oexle K., Grehl H., Kuhlenbaumer G., Van Broeckhoven C., Ringelstein E. B., Funke H. (2000). PMP22 Thr118Met is
not a clinically relevant CMT1 marker. J. Neurol.

[324] Shy M. E., Scavina M. T., Clark A., Krajewski K. M., Li J., Kamholz J., Kolodny E., Szigeti K., Fischer R. A., Saifi G. M., Scherer S. S., Lupski J. R. (2006). T118M PMP22 mutation
causes partial loss of function and HNPP-like neuropathy. Ann. Neurol..

[325] Seeman P., Mazanec R., Marikova T., Rautenstrauss B. (1999). Charcot-Marie-Tooth
1A: Heterozygous T118M Mutation over a CMT1A Duplication Has No Influence
on the Phenotype. Ann. N.Y. Acad. Sci..

[326] Russo M., Laura M., Polke J. M., Davis M. B., Blake J., Brandner S., Hughes R. A., Houlden H., Bennett D. L., Lunn M. P., Reilly M. M. (2011). Variable
phenotypes
are associated with PMP22 missense mutations. Neuromuscul Disord.

[327] Nelis E., Holmberg B., Adolfsson R., Holmgren G., van Broeckhoven C. (1997). PMP22 Thr(118)­Met: recessive CMT1
mutation or polymorphism?. Nat. Genet..

[328] Ho K. W. D., Jerath N. U. (2018). T118M Variant of
PMP22 Gene Presents
with Painful Peripheral Neuropathy and Varying Charcot-Marie-Tooth
Features: A Case Series and Review of the Literature. Case Rep. Genet.

[329] Gudmundsson S., Singer-Berk M., Watts N. A., Phu W., Goodrich J. K., Solomonson M., Genome Aggregation Database C., Rehm H. L., MacArthur D. G., O’Donnell-Luria A. (2022). Variant interpretation
using population databases: Lessons from gnomAD. Hum Mutat.

[330] Stefanski K. M., Li G. C., Marinko J. T., Carter B. D., Samuels D. C., Sanders C. R. (2023). Reply to Record et al. ″The
role of PMP22 T118M in Charcot-Marie-Tooth disease remains unsolved″. J. Biol. Chem..

[331] Record C. J., Laura M., Rossor A. M., Reilly M. M. (2023). The role
of PMP22 T118M in Charcot-Marie-Tooth disease remains unsolved. J. Biol. Chem..

[332] Jerath N. U., Kamholz J., Grider T., Harper A., Swenson A., Shy M. E. (2015). Coexistence of a T118M PMP22 missense
mutation and chromosome 17 (17p11.2-p12) deletion. Muscle Nerve.

[333] Warner L. E., Garcia C. A., Lupski J. R. (1999). Hereditary peripheral
neuropathies: clinical forms, genetics, and molecular mechanisms. Annu. Rev. Med..

[334] Chance P. F. (2006). Inherited focal, episodic neuropathies: hereditary
neuropathy with liability to pressure palsies and hereditary neuralgic
amyotrophy. Neuromolecular Med..

[335] Bai Y., Zhang X., Katona I., Saporta M. A., Shy M. E., O’Malley H. A., Isom L. L., Suter U., Li J. (2010). Conduction
block in PMP22 deficiency. J. Neurosci..

[336] Vallat J. M., Sindou P., Preux P. M., Tabaraud F., Milor A. M., Couratier P., LeGuern E., Brice A. (1996). Ultrastructural
PMP22 expression in inherited demyelinating neuropathies. Ann. Neurol..

[337] Koike H., Hirayama M., Yamamoto M., Ito H., Hattori N., Umehara F., Arimura K., Ikeda S., Ando Y., Nakazato M., Kaji R., Hayasaka K., Nakagawa M., Sakoda S., Matsumura K., Onodera O., Baba M., Yasuda H., Saito T., Kira J., Nakashima K., Oka N., Sobue G. (2005). Age associated
axonal features in HNPP with 17p11.2 deletion in Japan. J. Neurol Neurosurg Psychiatry.

[338] Mouton P., Tardieu S., Gouider R., Birouk N., Maisonobe T., Dubourg O., Brice A., LeGuern E., Bouche P. (1999). Spectrum of clinical and electrophysiologic
features
in HNPP patients with the 17p11.2 deletion. Neurology.

[339] Winzeler E. A., Shoemaker D. D., Astromoff A., Liang H., Anderson K., Andre B., Bangham R., Benito R., Boeke J. D., Bussey H., Chu A. M., Connelly C., Davis K., Dietrich F., Dow S. W., El Bakkoury M., Foury F., Friend S. H., Gentalen E., Giaever G., Hegemann J. H., Jones T., Laub M., Liao H., Liebundguth N., Lockhart D. J., Lucau-Danila A., Lussier M., M’Rabet N., Menard P., Mittmann M., Pai C., Rebischung C., Revuelta J. L., Riles L., Roberts C. J., Ross-MacDonald P., Scherens B., Snyder M., Sookhai-Mahadeo S., Storms R. K., Veronneau S., Voet M., Volckaert G., Ward T. R., Wysocki R., Yen G. S., Yu K., Zimmermann K., Philippsen P., Johnston M., Davis R. W. (1999). Functional
characterization of the S. cerevisiae genome by gene deletion and
parallel analysis. Science.

[340] Giaever G., Chu A. M., Ni L., Connelly C., Riles L., Veronneau S., Dow S., Lucau-Danila A., Anderson K., Andre B., Arkin A. P., Astromoff A., El-Bakkoury M., Bangham R., Benito R., Brachat S., Campanaro S., Curtiss M., Davis K., Deutschbauer A., Entian K. D., Flaherty P., Foury F., Garfinkel D. J., Gerstein M., Gotte D., Guldener U., Hegemann J. H., Hempel S., Herman Z., Jaramillo D. F., Kelly D. E., Kelly S. L., Kotter P., LaBonte D., Lamb D. C., Lan N., Liang H., Liao H., Liu L., Luo C., Lussier M., Mao R., Menard P., Ooi S. L., Revuelta J. L., Roberts C. J., Rose M., Ross-Macdonald P., Scherens B., Schimmack G., Shafer B., Shoemaker D. D., Sookhai-Mahadeo S., Storms R. K., Strathern J. N., Valle G., Voet M., Volckaert G., Wang C. Y., Ward T. R., Wilhelmy J., Winzeler E. A., Yang Y., Yen G., Youngman E., Yu K., Bussey H., Boeke J. D., Snyder M., Philippsen P., Davis R. W., Johnston M. (2002). Functional profiling of the Saccharomyces
cerevisiae genome. Nature.

[341] Costanzo M., Baryshnikova A., Myers C. L., Andrews B., Boone C. (2011). Charting the genetic
interaction map of a cell. Curr. Opin Biotechnol.

[342] Haney C., Snipes G. J., Shooter E. M., Suter U., Garcia C., Griffin J. W., Trapp B. D. (1996). Ultrastructural
distribution of PMP22 in Charcot-Marie-Tooth disease type 1A. J. Neuropathol Exp Neurol.

[343] El-Hage O., Mikdache A., Boueid M. J., Degerny C., Tawk M. (2025). Schwann cells have a limited window
of time in which to initiate
myelination signaling during early migration in vivo. Cells Dev.

[344] Yoshikawa H., Nishimura T., Nakatsuji Y., Fujimura H., Himoro M., Hayasaka K., Sakoda S., Yanagihara T. (1994). Elevated expression
of messenger RNA for peripheral
myelin protein 22 in biopsied peripheral nerves of patients with Charcot-Marie-Tooth
disease type 1A. Ann. Neurol..

[345] Svaren J., Moran J. J., Wu X., Zuccarino R., Bacon C., Bai Y., Ramesh R., Gutmann L., Anderson D. M., Pavelec D., Shy M. E. (2019). Schwann
cell transcript
biomarkers for hereditary neuropathy skin biopsies. Ann. Neurol..

[346] Msheik Z., Durand S., Pinault E., Caillaud M., Vignaud L., Billet F., El Massry M., Desmouliere A. (2023). Charcot-Marie-Tooth-1A
and sciatic nerve crush rat
models: insights from proteomics. Neural Regen
Res..

[347] Chittoor V. G., Sooyeon L., Rangaraju S., Nicks J. R., Schmidt J. T., Madorsky I., Narvaez D. C., Notterpek L. (2013). Biochemical
characterization of protein quality control
mechanisms during disease progression in the C22 mouse model of CMT1A. ASN Neuro.

[348] Van
Lent J., Vendredy L., Adriaenssens E., Da Silva Authier T., Asselbergh B., Kaji M., Weckhuysen S., Van Den Bosch L., Baets J., Timmerman V. (2023). Downregulation
of PMP22 ameliorates myelin defects in iPSC-derived human organoid
cultures of CMT1A. Brain.

[349] Wiseman R. L., Mesgarzadeh J. S., Hendershot L. M. (2022). Reshaping
endoplasmic reticulum quality control through the unfolded protein
response. Mol. Cell.

[350] Libberecht K., Vangansewinkel T., Van Den Bosch L., Lambrichts I., Wolfs E. (2023). Proteostasis plays
an important role
in demyelinating Charcot Marie Tooth disease. Biochem. Pharmacol..

[351] D’Antonio M., Feltri M. L., Wrabetz L. (2009). Myelin under stress. J. Neurosci Res..

[352] Hantke J., Carty L., Wagstaff L. J., Turmaine M., Wilton D. K., Quintes S., Koltzenburg M., Baas F., Mirsky R., Jessen K. R. (2014). c-Jun activation
in Schwann cells protects against loss of sensory axons in inherited
neuropathy. Brain.

[353] Perea J., Robertson A., Tolmachova T., Muddle J., King R. H., Ponsford S., Thomas P. K., Huxley C. (2001). Induced myelination and demyelination
in a conditional
mouse model of Charcot-Marie-Tooth disease type 1A. Hum. Mol. Genet..

[354] Nishimura T., Yoshikawa H., Fujimura H., Sakoda S., Yanagihara T. (1996). Accumulation of peripheral myelin protein 22 in onion
bulbs and Schwann cells of biopsied nerves from patients with Charcot-Marie-Tooth
disease type 1A. Acta Neuropathol.

[355] Katona I., Wu X., Feely S. M., Sottile S., Siskind C. E., Miller L. J., Shy M. E., Li J. (2009). PMP22 expression
in dermal nerve myelin from patients with CMT1A. Brain.

[356] Gabriel J. M., Erne B., Pareyson D., Sghirlanzoni A., Taroni F., Steck A. J. (1997). Gene dosage effects in hereditary
peripheral neuropathy. Expression of peripheral myelin protein 22
in Charcot-Marie-Tooth disease type 1A and hereditary neuropathy with
liability to pressure palsies nerve biopsies. Neurology.

[357] Anani T., Sindou P., Richard L., Diot M., Vallat J. M. (1999). Ultrastructural
immunocytochemical abnormalities of
peripheral myelin proteins in hereditary sensory-motor neuropathies:
12 cases. Ann. N.Y. Acad. Sci..

[358] Hanemann C. O., Stoll G., D’Urso D., Fricke W., Martin J. J., Van Broeckhoven C., Mancardi G. L., Bartke I., Muller H. W. (1994). Peripheral myelin
protein-22 expression in Charcot-Marie-Tooth disease type 1a sural
nerve biopsies. J. Neurosci Res..

[359] Ohnishi A., Yamamoto T., Ikeda M. (2000). Small axons
relative
to number of myelin lamellae in Charcot-Marie-Tooth disease 1A with
peripheral myelin protein 22 gene duplication. J. UOEH.

[360] Gabreels-Festen A. A., Bolhuis P. A., Hoogendijk J. E., Valentijn L. J., Eshuis E. J., Gabreels F. J. (1995). Charcot-Marie-Tooth
disease type 1A: morphological phenotype of the 17p duplication versus
PMP22 point mutations. Acta Neuropathol.

[361] Fabrizi G. M., Simonati A., Morbin M., Cavallaro T., Taioli F., Benedetti M. D., Edomi P., Rizzuto N. (1998). Clinical and
pathological correlations in Charcot-Marie-Tooth neuropathy type 1A
with the 17p11.2p12 duplication: a cross-sectional morphometric and
immunohistochemical study in twenty cases. Muscle
Nerve.

[362] Hanemann C. O., Gabreels-Festen A. A., Stoll G., Muller H. W. (1997). Schwann
cell differentiation in Charcot-Marie-Tooth disease type 1A (CMT1A):
normal number of myelinating Schwann cells in young CMT1A patients
and neural cell adhesion molecule expression in onion bulbs. Acta Neuropathol.

[363] Erdem S., Mendell J. R., Sahenk Z. (1998). Fate of Schwann cells
in CMT1A and HNPP: evidence for apoptosis. J.
Neuropathol Exp Neurol.

[364] Verhamme C., King R. H., ten Asbroek A. L., Muddle J. R., Nourallah M., Wolterman R., Baas F., van Schaik I. N. (2011). Myelin and axon pathology in a long-term
study of PMP22-overexpressing mice. J. Neuropathol
Exp Neurol.

[365] Liehr T., Grehl H., Rautenstrauss B. (1997). Accumulation
of peripheral myelin protein 22 (PMP22) in onion bulbs of nerves biopsied
from patients with different subtypes of Charcot-Marie-Tooth disease
type 1. Acta Neuropathol.

[366] Koike H., Iijima M., Mori K., Yamamoto M., Hattori N., Katsuno M., Tanaka F., Watanabe H., Doyu M., Yoshikawa H., Sobue G. (2007). Nonmyelinating Schwann
cell involvement with well-preserved unmyelinated axons in Charcot-Marie-Tooth
disease type 1A. J. Neuropathol Exp Neurol.

[367] Fledrich R., Akkermann D., Schutza V., Abdelaal T. A., Hermes D., Schaffner E., Soto-Bernardini M. C., Gotze T., Klink A., Kusch K., Krueger M., Kungl T., Frydrychowicz C., Mobius W., Bruck W., Mueller W. C., Bechmann I., Sereda M. W., Schwab M. H., Nave K. A., Stassart R. M. (2019). NRG1 type
I dependent autoparacrine
stimulation of Schwann cells in onion bulbs of peripheral neuropathies. Nat. Commun..

[368] Huang D., Meng L., Jiang L., Wang Z., Chen L., Yuan Y. (2023). Evaluation of the median
nerve by
shear wave elastography in patients with Charcot-Marie-Tooth disease
type 1A. Med. Ultrason.

[369] Saporta M. A., Katona I., Lewis R. A., Masse S., Shy M. E., Li J. (2009). Shortened internodal
length of dermal
myelinated nerve fibres in Charcot-Marie-Tooth disease type 1A. Brain.

[370] Nobbio L., Gherardi G., Vigo T., Passalacqua M., Melloni E., Abbruzzese M., Mancardi G., Nave K. A., Schenone A. (2006). Axonal damage and demyelination in long-term dorsal
root ganglia cultures from a rat model of Charcot-Marie-Tooth type
1A disease. Eur. J. Neurosci.

[371] Erickson R. R., Dunning L. M., Holtzman J. L. (2006). The effect
of aging
on the chaperone concentrations in the hepatic, endoplasmic reticulum
of male rats: the possible role of protein misfolding due to the loss
of chaperones in the decline in physiological function seen with age. J. Gerontol A Biol. Sci. Med. Sci..

[372] Nodera H., Bostock H., Kuwabara S., Sakamoto T., Asanuma K., Jia-Ying S., Ogawara K., Hattori N., Hirayama M., Sobue G., Kaji R. (2004). Nerve excitability
properties in Charcot-Marie-Tooth disease type 1A. Brain.

[373] Ang E. T., Schafer R., Baltensperger R., Wernig A., Celio M., Oliver S. S. (2010). Motor axonal sprouting
and neuromuscular junction loss in an animal model of Charcot-Marie-Tooth
disease. J. Neuropathol Exp Neurol.

[374] Li J. (2017). Caveats in the Established Understanding
of CMT1A. Ann. Clin Transl Neurol.

[375] Djuanda D., He B., Liu X., Xu S., Zhang Y., Xu Y., Zhu Z. (2021). Comprehensive Analysis
of Age-related Changes in Lipid Metabolism and Myelin Sheath Formation
in Sciatic Nerves. J. Mol. Neurosci.

[376] Pareek S., Suter U., Snipes G. J., Welcher A. A., Shooter E. M., Murphy R. A. (1993). Detection and processing
of peripheral
myelin protein PMP22 in cultured Schwann cells. J. Biol. Chem..

[377] White J. B., Sanchez K. L., Currais A., Soriano-Castell D., Maher P., Soriano S. (2025). Ferroptosis and Charcot-Marie-Tooth
Disease 1A: Emerging Evidence for a Pathogenic Association. Antioxidants (Basel).

[378] Nazareth L., St John J., Murtaza M., Ekberg J. (2021). Phagocytosis
by Peripheral Glia: Importance for Nervous System Functions and Implications
in Injury and Disease. Front Cell Dev Biol..

[379] Kaminska J., Kochanski A. (2025). A Role of
Inflammation in Charcot-Marie-Tooth
Disorders-In a Perspective of Treatment?. Int.
J. Mol. Sci..

[380] Michailidou I., Vreijling J., Rumpf M., Loos M., Koopmans B., Vlek N., Straat N., Agaser C., Kuipers T. B., Mei H., Baas F., Fluiter K. (2023). The systemic
inhibition of the terminal complement system reduces neuroinflammation
but does not improve motor function in mouse models of CMT1A with
overexpressed PMP22. Curr. Res. Neurobiol.

[381] Gillen C., Gleichmann M., Spreyer P., Muller H. W. (1995). Differentially
expressed genes after peripheral nerve injury. J. Neurosci Res..

[382] McLean J. W., Wilson J. A., Tian T., Watson J. A., VanHart M., Bean A. J., Scherer S. S., Crossman D. K., Ubogu E., Wilson S. M. (2022). Disruption of Endosomal Sorting in
Schwann Cells Leads to Defective Myelination and Endosomal Abnormalities
Observed in Charcot-Marie-Tooth Disease. J.
Neurosci..

[383] Bai Y., Treins C., Volpi V. G., Scapin C., Ferri C., Mastrangelo R., Touvier T., Florio F., Bianchi F., Del Carro U., Baas F. F., Wang D., Miniou P., Guedat P., Shy M. E., D’Antonio M. (2022). Treatment
with IFB-088 Improves Neuropathy in CMT1A and CMT1B Mice. Mol. Neurobiol.

[384] Vigo T., Nobbio L., Hummelen P. V., Abbruzzese M., Mancardi G., Verpoorten N., Verhoeven K., Sereda M. W., Nave K. A., Timmerman V., Schenone A. (2005). Experimental Charcot-Marie-Tooth type 1A: a cDNA microarrays
analysis. Mol. Cell Neurosci.

[385] Fledrich R., Stassart R. M., Sereda M. W. (2012). Murine
therapeutic
models for Charcot-Marie-Tooth (CMT) disease. Br Med. Bull..

[386] Hanemann C. O., Gabreels-Fasten A. A., Muller H. W., Stoll G. (1996). Low affinity
NGF receptor expression in CMT1A nerve biopsies of different disease
stages. Brain.

[387] Nobbio L., Fiorese F., Vigo T., Cilli M., Gherardi G., Grandis M., Melcangi R. C., Mancardi G., Abbruzzese M., Schenone A. (2009). Impaired expression
of ciliary neurotrophic
factor in Charcot-Marie-Tooth type 1A neuropathy. J. Neuropathol Exp Neurol.

[388] Silva A., Prior R., D’Antonio M., Swinnen J. V., Van Den Bosch L. (2025). Lipid metabolism alterations in peripheral
neuropathies. Neuron.

[389] Reilly M. M., Rossor A. M. (2020). Humans: the ultimate
animal models. J. Neurol Neurosurg Psychiatry.

[390] Meyer Zu Horste G., Nave K. A. (2006). Animal models of
inherited neuropathies. Curr. Opin Neurol.

[391] Sereda M. W., Nave K. A. (2006). Animal models of
Charcot-Marie-Tooth
disease type 1A. Neuromolecular Med..

[392] Huxley C., Passage E., Manson A., Putzu G., Figarella-Branger D., Pellissier J. F., Fontes M. (1996). Construction of a mouse
model of Charcot-Marie-Tooth disease type 1A by pronuclear injection
of human YAC DNA. Hum. Mol. Genet..

[393] Robertson A. M., Huxley C., King R. H., Thomas P. K. (1999). Development
of early postnatal peripheral nerve abnormalities in Trembler-J and
PMP22 transgenic mice. J. Anat.

[394] Prior R., Verschoren S., Vints K., Jaspers T., Rossaert E., Klingl Y. E., Silva A., Hersmus N., Van Damme P., Van Den Bosch L. (2022). HDAC3 Inhibition Stimulates Myelination
in a CMT1A Mouse Model. Mol. Neurobiol.

[395] Sereda M., Griffiths I., Puhlhofer A., Stewart H., Rossner M. J., Zimmermann F., Magyar J. P., Schneider A., Hund E., Meinck H. M., Suter U., Nave K. A. (1996). A transgenic rat model of Charcot-Marie-Tooth
disease. Neuron.

[396] Robertson A. M., Perea J., McGuigan A., King R. H., Muddle J. R., Gabreels-Festen A. A., Thomas P. K., Huxley C. (2002). Comparison
of a new pmp22 transgenic mouse line with other mouse models and human
patients with CMT1A. J. Anat.

[397] Taruta A., Matsumoto S. I., Masuda Y., Hiyoshi T., Harada A., Kosugi Y., Nakashima M. (2025). Characterization
of a humanized mouse model of Charcot-Marie-Tooth type 1A for the
discovery of human PMP22-targeting drugs. Front
Neurol.

[398] Gautier B., Hajjar H., Soares S., Berthelot J., Deck M., Abbou S., Campbell G., Ceprian M., Gonzalez S., Fovet C. M., Schutza V., Jouvenel A., Rivat C., Zerah M., Francois V., Le Guiner C., Aubourg P., Fledrich R., Tricaud N. (2021). AAV2/9-mediated
silencing
of PMP22 prevents the development of pathological features in a rat
model of Charcot-Marie-Tooth disease 1 A. Nat.
Commun..

[399] Espallergues J., Cadiet J., Souab F., Choquet O., Swisser F., Bigeleisen P., Maleysson V., Sola M. L., van Hameren G., Tricaud N. (2025). Perineural delivery
of AAV2/9 in non-human primates is a safe and efficient route for
gene therapy in Charcot-Marie-Tooth diseases. Mol. Ther Methods Clin Dev.

[400] Stavrou M., Kagiava A., Sargiannidou I., Kleopa K. A. (2025). Developing a gene
therapy for Charcot-Marie-Tooth disease:
progress and challenges. Regen Med..

[401] Mariki A., Kohlmeier K. A., Mousavi S. M., Shabani M. (2025). A systematic
review of CRISPR applications in demyelinating peripheral nervous
system disorders. Regen Med..

[402] Beloribi-Djefaflia S., Attarian S. (2023). Treatment of Charcot-Marie-Tooth
neuropathies. Rev. Neurol (Paris).

[403] Kwon, H. M. ; Kim, H. S. ; Chi, S. A. ; Nam, S. H. ; Kim, H. J. ; Kim, S. B. ; Choi, B. O. An open-label single-arm phase 1/2a study to evaluate the safety and exploratory efficacy of a VM202 in patients with Charcot-Marie-Tooth disease 1A. Orphanet J. Rare Dis 2026, 21. 10.1186/s13023-026-04252-2. PMC13072572 41787447

[404] Stavrou M., Wallace L. M., Thangaraj M. P., Taylor N. K., Kagiava A., Papacharalambous R., McAllister C., Zender G., Saad N. Y., Bayazit M. B., Heslegrave A., Tryfonos C., Richter J., Zetterberg H., Price B., Salzman R., Kleopa K. A., Harper S. Q. (2026). Safety,
efficacy, and distal nerve Schwann cell biodistribution in mice and
NHPs to support translation of AAV9 RNAi therapy for CMT1A. Mol. Ther Nucleic Acids.

[405] Zhao H. T., Damle S., Ikeda-Lee K., Kuntz S., Li J., Mohan A., Kim A., Hung G., Scheideler M. A., Scherer S. S., Svaren J., Swayze E. E., Kordasiewicz H. B. (2018). PMP22 antisense
oligonucleotides
reverse Charcot-Marie-Tooth disease type 1A features in rodent models. J. Clin Invest.

[406] Stavrou, M. ; Kagiava, A. ; Choudury, S. G. ; Jennings, M. J. ; Wallace, L. M. ; Fowler, A. M. ; Heslegrave, A. ; Richter, J. ; Tryfonos, C. ; Christodoulou, C. ; Zetterberg, H. ; Horvath, R. ; Harper, S. Q. ; Kleopa, K. A. A translatable RNAi-driven gene therapy silences PMP22/Pmp22 genes and improves neuropathy in CMT1A mice, J. Clin Invest 2022, 132. 10.1172/JCI159814. PMC9246392 35579942

[407] Serfecz J., Bazick H., Al Salihi M. O., Turner P., Fields C., Cruz P., Renne R., Notterpek L. (2019). Downregulation
of the human peripheral myelin protein
22 gene by miR-29a in cellular models of Charcot-Marie-Tooth disease. Gene Ther..

[408] Boutary S., Khalaf G., Landesman Y., Madani M. E., Desmaele D., Piguet F., Alonso R., Banchi E. G., Adams D., Massaad C., Massaad-Massade L. (2025). Therapeutic
potential of siRNA PMP22-SQ nanoparticles for Charcot-Marie-Tooth
1A neuropathy in rodents and non-human primates. Int. J. Pharm..

[409] Boutary S., Caillaud M., El Madani M., Vallat J. M., Loisel-Duwattez J., Rouyer A., Richard L., Gracia C., Urbinati G., Desmaele D., Echaniz-Laguna A., Adams D., Couvreur P., Schumacher M., Massaad C., Massaad-Massade L. (2021). Squalenoyl
siRNA PMP22 nanoparticles
are effective in treating mouse models of Charcot-Marie-Tooth disease
type 1 A. Commun. Biol..

[410] Dranchak P., Moran J. J., MacArthur R., Lopez-Anido C., Inglese J., Svaren J. (2018). Genome-Edited Cell
Lines for High-Throughput Screening. Methods
Mol. Biol..

[411] Inglese J., Dranchak P., Moran J. J., Jang S. W., Srinivasan R., Santiago Y., Zhang L., Guha R., Martinez N., MacArthur R., Cost G. J., Svaren J. (2014). Genome editing-enabled
HTS assays expand drug target pathways for Charcot-Marie-tooth disease. ACS Chem. Biol..

[412] Jang S. W., Lopez-Anido C., MacArthur R., Svaren J., Inglese J. (2012). Identification
of drug modulators
targeting gene-dosage disease CMT1A. ACS Chem.
Biol..

[413] Martinez N. J., Braisted J. C., Dranchak P. K., Moran J. J., Larson H., Queme B., Pak E., Dutra A., Rai G., Cheng K. C., Svaren J., Inglese J. (2021). Genome-Edited Coincidence
and PMP22-HiBiT Fusion Reporter Cell Lines Enable an Artifact-Suppressive
Quantitative High-Throughput Screening Strategy for PMP22 Gene-Dosage
Disorder Drug Discovery. ACS Pharmacol Transl
Sci..

[414] Bacalhau M., Camargo M., Magalhaes-Ghiotto G.
A. V., Drumond S., Castelletti C. H. M., Lopes-Pacheco M. (2023). Elexacaftor-Tezacaftor-Ivacaftor:
A Life-Changing Triple Combination of CFTR Modulator Drugs for Cystic
Fibrosis. Pharmaceuticals (Basel).

[415] Agrahari A. K., C G.
P. D. (2017). A Computational Approach
to Identify
a Potential Alternative Drug With Its Positive Impact Toward PMP22. J. Cell Biochem.

[416] Nicks J., Lee S., Harris A., Falk D. J., Todd A. G., Arredondo K., Dunn W. A., Notterpek L. (2014). Rapamycin
improves peripheral nerve myelination while
it fails to benefit neuromuscular performance in neuropathic mice. Neurobiol Dis.

[417] Khajavi M., Shiga K., Wiszniewski W., He F., Shaw C. A., Yan J., Wensel T. G., Snipes G. J., Lupski J. R. (2007). Oral curcumin mitigates the clinical and neuropathologic
phenotype of the Trembler-J mouse: a potential therapy for inherited
neuropathy. Am. J. Hum. Genet..

[418] Chittoor-Vinod V. G., Bazick H., Todd A. G., Falk D., Morelli K. H., Burgess R. W., Foster T. C., Notterpek L. (2019). HSP90 Inhibitor,
NVP-AUY922, Improves Myelination in Vitro and Supports the Maintenance
of Myelinated Axons in Neuropathic Mice. ACS
Chem. Neurosci..

[419] Caillaud M., Msheik Z., Ndong-Ntoutoume G. M., Vignaud L., Richard L., Favreau F., Faye P. A., Sturtz F., Granet R., Vallat J. M., Sol V., Desmouliere A., Billet F. (2020). Curcumin-cyclodextrin/cellulose nanocrystals
improve the phenotype of Charcot-Marie-Tooth-1A transgenic rats through
the reduction of oxidative stress. Free Radic
Biol. Med..

[420] Okamoto Y., Pehlivan D., Wiszniewski W., Beck C. R., Snipes G. J., Lupski J. R., Khajavi M. (2013). Curcumin facilitates
a transitory cellular stress response in Trembler-J. mice. Hum. Mol. Genet..

[421] Lin W., Popko B. (2009). Endoplasmic reticulum stress in disorders of myelinating
cells. Nat. Neurosci.

[422] Fledrich R., Akkermann D., Schutza V., Abdelaal T. A., Hermes D., Schaffner E., Soto-Bernardini M. C., Gotze T., Klink A., Kusch K., Krueger M., Kungl T., Frydrychowicz C., Mobius W., Bruck W., Mueller W. C., Bechmann I., Sereda M. W., Schwab M. H., Nave K. A., Stassart R. M. (2019). Publisher
Correction: NRG1 type I
dependent autoparacrine stimulation of Schwann cells in onion bulbs
of peripheral neuropathies. Nat. Commun..

[423] Nicks J. R., Lee S., Kostamo K. A., Harris A. B., Sookdeo A. M., Notterpek L. (2013). Long-term
analyses of innervation
and neuromuscular integrity in the Trembler-J. mouse model of Charcot-Marie-Tooth
disease. J. Neuropathol Exp Neurol.

[424] Madorsky I., Opalach K., Waber A., Verrier J. D., Solmo C., Foster T., Dunn W. A., Notterpek L. (2009). Intermittent fasting alleviates the
neuropathic phenotype
in a mouse model of Charcot-Marie-Tooth disease. Neurobiol Dis.

[425] Zhou Y., Bazick H., Miles J. R., Fethiere A. I., Salihi M. O. A., Fazio S., Tavori H., Notterpek L. (2019). A neutral
lipid-enriched diet improves myelination and alleviates peripheral
nerve pathology in neuropathic mice. Exp. Neurol..

[426] Fledrich R., Abdelaal T., Rasch L., Bansal V., Schutza V., Brugger B., Luchtenborg C., Prukop T., Stenzel J., Rahman R. U., Hermes D., Ewers D., Mobius W., Ruhwedel T., Katona I., Weis J., Klein D., Martini R., Bruck W., Muller W. C., Bonn S., Bechmann I., Nave K. A., Stassart R. M., Sereda M. W. (2018). Targeting myelin lipid metabolism
as a potential therapeutic strategy in a model of CMT1A neuropathy. Nat. Commun..

[427] Woo Y. H., Schmidt N. E., Johansson J. O., Notterpek L. (2026). ABCA1: A Therapeutic Target for Improving Cholesterol
Homeostasis in Peripheral Neuropathies. Biomolecules.

[428] Young P., Suter U. (2001). Disease mechanisms
and potential
therapeutic strategies in Charcot-Marie-Tooth disease. Brain Res. Brain Res. Rev..

[429] Qi H., Wang X., Wu B., Chen J., Zhang G. (2025). The current
status of Charcot-Marie-Tooth disease type 1 A treatment. Acta Neurol Belg.

[430] Prukop T., Wernick S., Boussicault L., Ewers D., Jager K., Adam J., Winter L., Quintes S., Linhoff L., Barrantes-Freer A., Bartl M., Czesnik D., Zschuntzsch J., Schmidt J., Primas G., Laffaire J., Rinaudo P., Brureau A., Nabirotchkin S., Schwab M. H., Nave K. A., Hajj R., Cohen D., Sereda M. W. (2020). Synergistic PXT3003
therapy uncouples neuromuscular function from dysmyelination in male
Charcot-Marie-Tooth disease type 1A (CMT1A) rats. J. Neurosci Res..

[431] Roux K. J., Amici S. A., Notterpek L. (2004). The temporospatial
expression of peripheral myelin protein 22 at the developing blood-nerve
and blood-brain barriers. J. Comp Neurol.

[432] Liu H., Sahi J. (2025). Peripheral nerve barrier
and its implications for drug
delivery. Drug Discov Today.

[433] Goda S., Hammer J., Kobiler D., Quarles R. H. (1991). Expression
of the myelin-associated glycoprotein in cultures of immortalized
Schwann cells. J. Neurochem.

[434] Hai M., Muja N., DeVries G. H., Quarles R. H., Patel P. I. (2002). Comparative
analysis of Schwann cell lines as model systems for myelin gene transcription
studies. J. Neurosci Res..

[435] Lehmann H. C., Hoke A. (2010). Schwann cells as a
therapeutic target
for peripheral neuropathies. CNS Neurol Disord
Drug Targets.

[436] Sango K., Kawakami E., Yanagisawa H., Takaku S., Tsukamoto M., Utsunomiya K., Watabe K. (2012). Myelination in coculture of established
neuronal and
Schwann cell lines. Histochem Cell Biol..

[437] Porter S., Clark M. B., Glaser L., Bunge R. P. (1986). Schwann
cells stimulated to proliferate in the absence of neurons retain full
functional capability. J. Neurosci..

[438] Numata-Uematasu Y., Wakatsuki S., Kobayashi-Ujiie Y., Sakai K., Ichinohe N., Araki T. (2023). In vitro myelination
using explant culture of dorsal root ganglia: An efficient tool for
analyzing peripheral nerve differentiation and disease modeling. PLoS One.

[439] Ecob M. S. (1983). The application of organotypic nerve cultures to problems
in neurology with special reference to their potential use in research
into neuromuscular diseases. J. Neurol Sci..

[440] Takaku S., Yako H., Niimi N., Akamine T., Kawanami D., Utsunomiya K., Sango K. (2018). Establishment of a
myelinating co-culture system with a motor neuron-like cell line NSC-34
and an adult rat Schwann cell line IFRS1. Histochem
Cell Biol..

[441] Liu X., Ishikawa K. I., Hattori N., Akamatsu W. (2025). Establishment of an
In Vitro Disease Model of Charcot-Marie-Tooth Disease using Human
Induced Pluripotent Stem Cells. Juntendo Med.
J..

[442] Juneja M., Burns J., Saporta M. A., Timmerman V. (2019). Challenges
in modelling the Charcot-Marie-Tooth neuropathies for therapy development. J. Neurol Neurosurg Psychiatry.

[443] Agrawal M., Desai M., Ghumra S., Bhorkar Y., Sahoo P. K. (2026). Human induced pluripotent stem cell-based models for
studying neural repair. Prog. Neurobiol.

[444] Pawelec K. M., Yoon C., Giger R. J., Sakamoto J. (2019). Engineering
a platform for nerve regeneration with direct application to nerve
repair technology. Biomaterials.

[445] Khoshakhlagh P., Sivakumar A., Pace L. A., Sazer D. W., Moore M. J. (2018). Methods for fabrication
and evaluation of a 3D microengineered
model of myelinated peripheral nerve. J. Neural
Eng..

[446] Gribi S., du Bois de Dunilac S., Ghezzi D., Lacour S. P. (2018). A microfabricated
nerve-on-a-chip platform for rapid assessment of neural conduction
in explanted peripheral nerve fibers. Nat. Commun..

[447] Hara Y., Shiga T., Abe I., Tsujino A., Ichimura H., Okado N., Ochiai N. (2003). P0 mRNA expression
increases during gradual nerve elongation in adult rats. Exp. Neurol..

[448] Fledrich R., Schlotter-Weigel B., Schnizer T. J., Wichert S. P., Stassart R. M., Meyer zu Horste G., Klink A., Weiss B. G., Haag U., Walter M. C., Rautenstrauss B., Paulus W., Rossner M. J., Sereda M. W. (2012). A rat model of Charcot-Marie-Tooth
disease 1A recapitulates disease variability and supplies biomarkers
of axonal loss in patients. Brain.

[449] Chen Y., Shang T., Sun J., Ji Y., Gong L., Li A., Ding F., Shen M., Zhang Q. (2024). Characterization of sciatic nerve myelin sheath during development
in C57BL/6 mice. Eur. J. Neurosci.

[450] Zhang Y., Bekku Y., Dzhashiashvili Y., Armenti S., Meng X., Sasaki Y., Milbrandt J., Salzer J. L. (2012). Assembly and maintenance of nodes of ranvier rely on
distinct sources of proteins and targeting mechanisms. Neuron.

[451] Tao F., Beecham G. W., Rebelo A. P., Blanton S. H., Moran J. J., Lopez-Anido C., Svaren J., Abreu L., Rizzo D., Kirk C. A., Wu X., Feely S., Verhamme C., Saporta M. A., Herrmann D. N., Day J. W., Sumner C. J., Lloyd T. E., Li J., Yum S. W., Taroni F., Baas F., Choi B. O., Pareyson D., Scherer S. S., Reilly M. M., Shy M. E., Zuchner S., Inherited
Neuropathy C. (2019). Modifier Gene Candidates in Charcot-Marie-Tooth Disease
Type 1A: A Case-Only Genome-Wide Association Study. J. Neuromuscul Dis.

[452] Gonzaga-Jauregui C., Harel T., Gambin T., Kousi M., Griffin L. B., Francescatto L., Ozes B., Karaca E., Jhangiani S. N., Bainbridge M. N., Lawson K. S., Pehlivan D., Okamoto Y., Withers M., Mancias P., Slavotinek A., Reitnauer P. J., Goksungur M. T., Shy M., Crawford T. O., Koenig M., Willer J., Flores B. N., Pediaditrakis I., Us O., Wiszniewski W., Parman Y., Antonellis A., Muzny D. M., Baylor-Hopkins Center for Mendelian G., Katsanis N., Battaloglu E., Boerwinkle E., Gibbs R. A., Lupski J. R. (2015). Exome Sequence Analysis Suggests
that Genetic Burden Contributes to Phenotypic Variability and Complex
Neuropathy. Cell Rep.

[453] Li K., Crews C. M. (2022). PROTACs: past, present
and future. Chem. Soc. Rev..

[454] Li B., Li Y., Zhang J., Badama S., Zhao X., Wang L., Zhang T., Wang X., Yi X., Ding G. B., Wang X., Nie G. (2026). Lysosome-targeting
chimeras enable targeted protein degradation. Cell Chem. Biol..

